# Age-associated loss of lymphatic vessels promotes cardiac inflammation

**DOI:** 10.1038/s44161-026-00870-y

**Published:** 2026-09-15

**Authors:** Julian U. G. Wagner, Hamza Gulshan, Ibrahim Sultan, Salli Antila, Laia-Canes Esteve, David Rodriguez Morales, Mariano Ruz Jurado, David John, Emmanouil G. Solomonidis, Katja Schmitz, Josefine Panthel, Haris Kujundzic, Susanne Hille, Oliver J. Müller, Christoph Kugler, Haider Sami, Manfred Ogris, Simone-Franziska Glaser, Wesley T. Abplanalp, Tara Procida-Kowalski, Marek Bartkuhn, Evelyn Ullrich, Marion Muhly-Reinholz, Sarmad Ahmad Khan, Tarik Bozoglu, Christian Kupatt, Florian Buettner, Jochen Pöling, Thomas Braun, Kari Alitalo, Stefanie Dimmeler

**Affiliations:** 1 Institute for Cardiovascular Regeneration, Goethe-University Frankfurt, Frankfurt am Main, Germany; 2 Partner Site RhineMain, German Center for Cardiovascular Research (DZHK), Frankfurt am Main, Germany; 3 Cardiopulmonary Institute, Frankfurt am Main, Germany; 4 Translational Cancer Medicine Program, University of Helsinki, Helsinki, Finland; 5 Wihuri Research Institute, Biomedicum Helsinki, Helsinki, Finland; 6 Department of Cardiac Development and Remodelling, Max Planck Institute for Heart and Lung Research, Bad Nauheim, Germany; 7 Department of Internal Medicine V, University Hospital Schleswig-Holstein, University of Kiel, Kiel, Germany; 8 Partner Site North, German Center for Cardiovascular Research (DZHK), Kiel, Germany; 9 Faculty of Life Sciences, Department of Pharmaceutical Sciences, Laboratory of Macromolecular Cancer Therapeutics, University of Vienna, Vienna, Austria; 10 Institute for Lung Health, Justus Liebig University, Giessen, Germany; 11 Biomedical Informatics and Systems Medicine, Justus Liebig University, Giessen, Germany; 12 Experimental Immunology and Cell Therapy, Division of Pediatrics, University Hospital Frankfurt, Goethe University, Frankfurt am Main, Germany; 13 Frankfurt Cancer Institute, Goethe University, Frankfurt am Main, Germany; 14 German Cancer Consortium (DKTK), Frankfurt am Main, Germany; 15 German Cancer Research Center (DKFZ), Frankfurt am Main, Germany; 16 Goethe University, Frankfurt am Main, Germany; 17 Partner Site Munich/Munich Heart Alliance, German Center for Cardiovascular Research (DZHK), Munich, Germany; 18 Klinik und Poliklinik für Innere Medizin I, University Clinic rechts der Isar, Technical University of Munich, Munich, Germany; 19 Schüchtermann-Klinik, Bad Rothenfelde, Germany

**Keywords:** Cardiovascular biology, Ageing, Lymphatic vessels

## Abstract

Aging is a major risk factor for cardiovascular disease, but the role of the cardiac lymphatic vasculature in this process remains poorly understood. Here we show that aging reduces cardiac lymphatic vessel density in humans and mice and induces structural remodeling characterized by tighter, zipper-like endothelial junctions. These changes are associated with immune cell infiltration, fibrinogen and amyloid accumulation, and myocardial edema. Selective reduction of cardiac lymphatics in young mice, through *Flt4* (VEGFR3) depletion or soluble *Flt4* overexpression, recapitulates key features of cardiac aging, including inflammation and impaired lymphatic integrity. Mechanistically, aging induces selective upregulation of nuclear interleukin-33 (IL-33) in lymphatic endothelial cells. Unlike extracellular IL-33, nuclear IL-33 promotes lymphatic endothelial cell death and junctional remodeling. We identify VEGFC as an age-sensitive regulator that declines with aging and suppresses IL-33. Cardiac *Vegfc* overexpression or Il33 silencing restores lymphatic density and tissue homeostasis in aged hearts, identifying a potential therapeutic target for age-related cardiac dysfunction.

## Abstract (web summary)

Wagner et al. reveal that age-related impairment of lymphatic integrity is driven by increased nuclear interleukin-33 expression in lymphatic endothelial cells and can be counteracted by cardiac Vegfc overexpression.

## Main

Aging profoundly impacts various physiological processes and drives structural and functional decline in cardiac tissue. These changes include reduced left ventricular diastolic function, myocardial fibrosis, cardiac amyloidosis and microvascular dysfunction. Collectively, they increase the risk of chronic diastolic heart failure in the elderly. Age-related cardiac dysfunction arises from prolonged exposure to genomic and epigenetic damage, oxidative stress, impaired autophagy and the accumulation of waste products and of inflammatory and senescent cells. In addition, age-related deterioration in the structural and functional integrity of the microcirculation imposes further stress on the heart. While the role of the blood vasculature in cardiac aging has been extensively studied^1–4^, the effects of aging on the cardiac lymphatic system remain poorly understood, warranting further investigation into its role in age-related cardiac dysfunction.

The cardiac lymphatic system plays a crucial role in maintaining heart health by removing excess fluid, proteins and metabolic waste products from the interstitial space of the myocardium^5–7^. This network comprises lymphatic capillaries, precollectors and larger collecting vessels, distributed throughout the epicardium, myocardium and endocardium, with the highest density observed in the epicardium^5,8,9^. By facilitating the removal of interstitial fluid, cardiac lymphatics prevent edema and maintain tissue fluid homeostasis^10–12^. Furthermore, they enable the transport of immune cells and antigens to lymph nodes, supporting immune surveillance and response, while clearing metabolic by-products and cellular debris from the heart tissue. Here, we demonstrated that aging leads to an early decline in lymphatic vessels in the heart, contributing to age-associated alterations in the cardiac tissue.

## Results

### Aging leads to cardiac lymphatic vessel decline

**Fig. 1:**
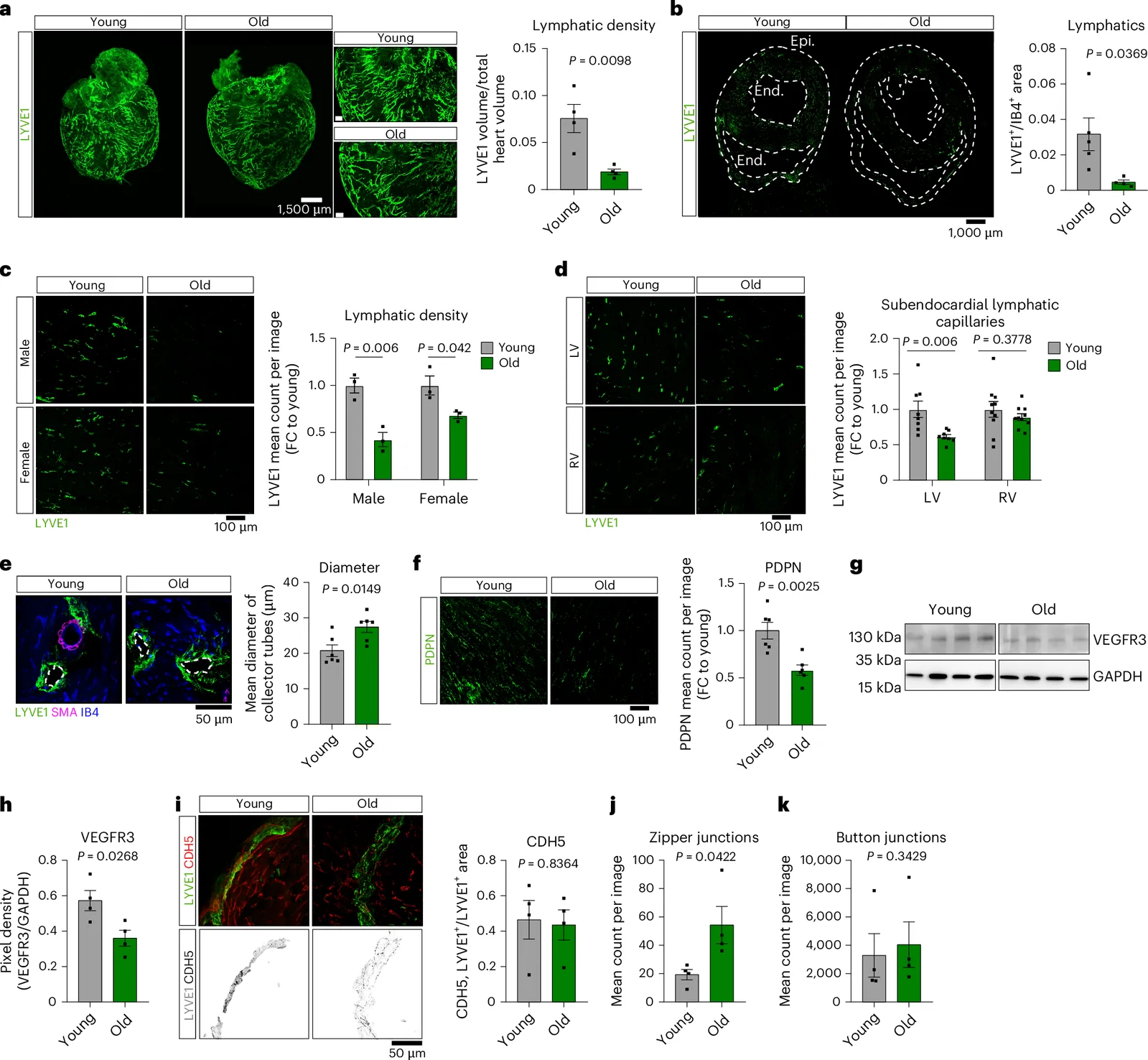
Lymphatic density is reduced in the ageing mouse heart. **a**, Whole-mount LYVE1 staining on hearts from 3-month-old (young) versus 20-month-old (old) mice (*n* = 4). **b**, Tile scan images of anti-LYVE1 staining on transversal sections from hearts of 3-month-old (young) versus 18–20-month-old (old) mice (*n* = 5 versus *n* = 4). **c**,**d**, Anti-LYVE1 staining on heart sections of 3-month-old (young) versus 18–20-month-old (old) male versus female mice (**c**) (*n* = 3) and in the left ventricular (LV; *n* = 8) and right ventricular (RV; *n* = 10) area (**d**). **e**, The mean diameter of lymphatic vessels (LYVE1) in young versus old mouse heart sections (*n* = 6). The dashed line indicates the inner surface of the vessels that were used for assessing the diameter. SMA and IB4 serve as the counterstain. **f**, Anti-PDPN staining on heart sections of 3-month-old (young) versus 18–20-month-old (old) male mice (*n* = 6). **g**,**h**, VEGFR3 expression in 3-month-old (young) versus 20-month-old (old) mouse heart lysates by WB (**g**). GAPDH served as the housekeeper control. Quantification is shown in **h** (*n* = 4). **i**–**k**, LymphEC junctions assessed by VE-cadherin (CDH5) staining (**i**) in LYVE1^+^ lymphatics (*n* = 4) in heart sections from 3-month-old (young) versus 20-month-old (old) male mice; the CDH5 area was quantified per lymphatic tube, and zipper junctions (**j**) and button junctions (**k**) were quantified. Data are expressed as mean, and error bars indicate s.e.m. After passing normality tests, *P* values were calculated using a two-sided, unpaired *t*-test. Data shown in **k** are not normally distributed, and the *P* value was calculated using an unpaired Mann–Whitney test. End., endocardium; epi., epicardium; FC, fold change. Source data

**Extended Data Fig. 1:**
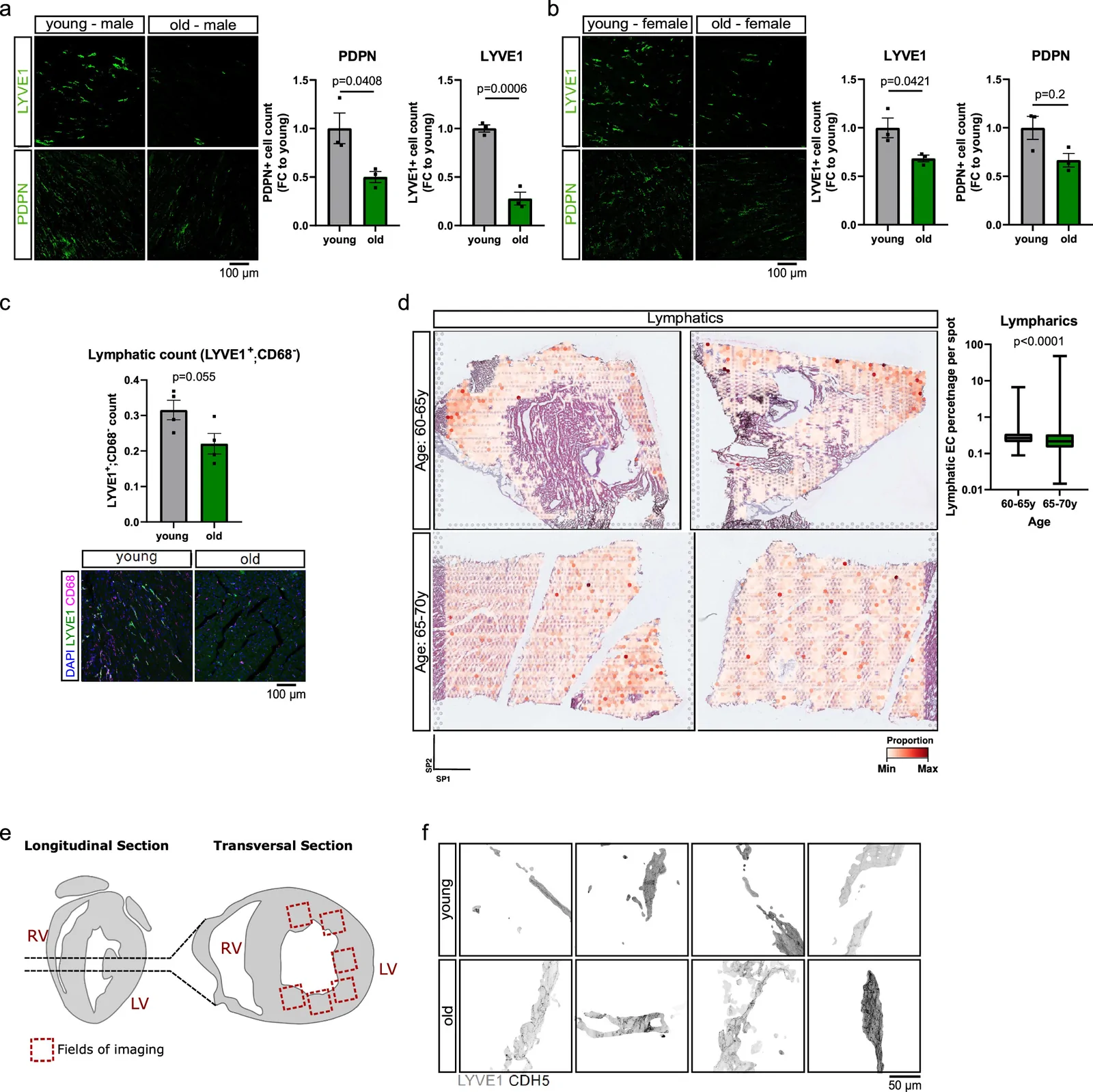
Lymphatic vessels decline in the aged left ventricle. **a-b** Lymphatic vessels were assessed using LYVE1 or PDPN staining in heart sections from young and old mice (n = 3) Panel A displays data from male animals while panel B shows data from female animals. **c** LYVE1, CD68 and DAPI staining in heart section from young versus old mice (n = 4). CD68-ngetaive but LYVE1^+^ structures were quantified versus DAPI. **d** Spatial transcriptomics of left ventricular samples from n = 2 60-65 years old subjects and form n = 3 65-70 years old subjects (data from Kanemaru et al., Nature 619, 801–810 (2023)). Spots expressing lymphatic specific marker genes are quantified. **e** Schematic representation of the imaging strategy to quantify lymphatic capillaries in the sub-endocardial area. **f** CDH5 staining in LYVE1-positive lymphatic vessels of hearts derived from young and aged mice. Shown are representative images. Data are expressed as mean and error bars indicate standard error of the mean. After passing normality tests, p-values were calculated using two-sided, unpaired T-test (a-c). The PDPN count in female hearts (b) is not normally distributed and p-value was calculated with an unpaired Mann-Whitney test. Source data

We determined the impact of aging on cardiac lymphatics in 18–20-month-old C57Bl/6 mice compared with 3-month-old young mice. Whole-mount staining of cardiac lymphatics with LYVE-1 revealed a substantial decline in lymphatic vasculature coverage in old mouse hearts (Fig. 1a). Consistently, cross-sectional LYVE-1 immunostainings showed a significant reduction in lymphatic vessels in both female and male aged mice (Fig. 1b,c and Extended Data Fig. 1a,b), in agreement with a very recent study^13^. As tissue-resident macrophages may express LYVE1 as well, we further included CD68 as a macrophage marker. To exclude LYVE1^+^ macrophages, we quantified CD68-negative LYVE1^+^ cells, which were less abundant in the aged heart section, thus confirming our findings (Extended Data Fig. 1c). Analysis of spatial transcriptomic data^14^ confirmed the reduction in lymphatic vessel density by aging in humans (Extended Data Fig. 1d). Lymphatic density declined predominantly in the subendocardial region of the left ventricle, whereas right ventricular lymphatics were preserved in old mice (Fig. 1d and Extended Data Fig. 1e). Epicardial lymphatic vessels additionally showed an enlarged lumen (Fig. 1e), as also has been reported for mesenteric lymphatics in aged rats^15^. Since LYVE-1 is also expressed by immune cell subpopulations, which may have confounded our analysis, we additionally used podoplanin (PDPN) as a second lymphatic marker. We found that PDPN^+^ and LYVE-1^+^ vessels also declined in hearts of 20-month-old mice (Fig. 1f and Extended Data Fig. 1a,b). Moreover, protein expression of the lymphatic vessel-enriched VEGF receptor 3 (VEGFR3; encoded by *Flt4*) was significantly declined in 20-month-old versus 3-month-old mice (Fig. 1g,h).

While lymphatic capillary endothelial cells (ECs) are interconnected via loose ‘button-like junctions’ to allow fluid and immune cell drainage, ECs of precollector and collector tubes are tightly interconnected via a tight ‘zipper-like junction’ to prevent vessel leakage^16^. Given these distinct junctional architectures, we additionally assessed junction morphology of the lymphatic vessels in the epicardial regions of the hearts (Fig. 1i). Analysis of CDH5^+^ junctions in LYVE-1^+^ vessels revealed that aging induces a relative increase in zipper-like structures, but the remaining lymphatics have a similar number of button-like structures (Fig. 1j,k and Extended Data Fig. 1f). Together, these data demonstrate a decline in lymphatic density associated with changes in the junctional architecture in the left ventricle of old mice.

### Reduced lymphatic density is associated with age-related defects in the heart

**Fig. 2:**
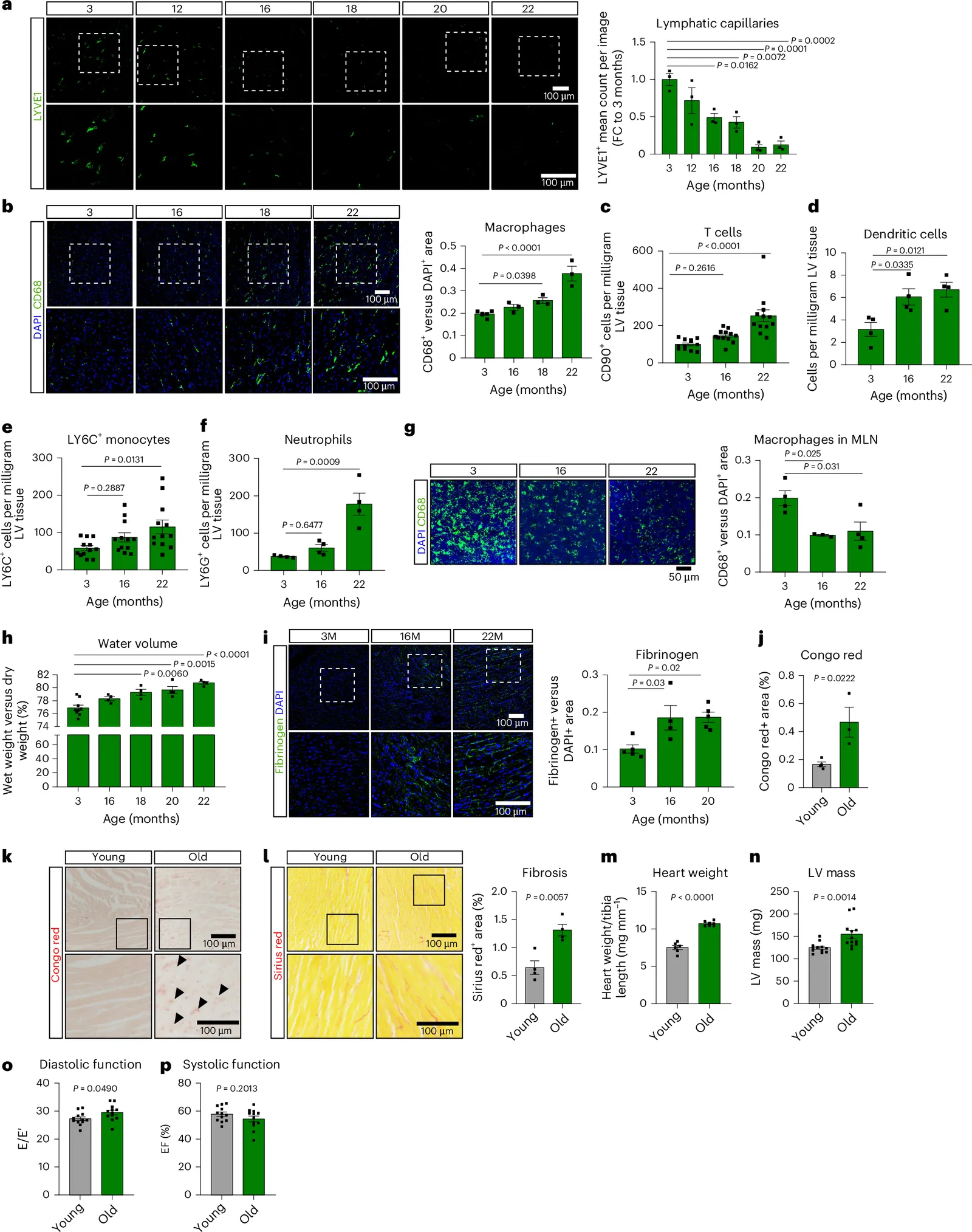
Lymphatic density is associated with cardiac draining in aged mice. **a**, Lymphatic capillary density (LYVE1) in hearts of 3-, 12-, 16-, 18-, 20- and 22-month-old male mice (*n* = 3 per group). **b**, CD68^+^ macrophages versus DAPI in heart sections from 3-, 16-, 18- and 22-month-old mice (3 months: *n* = 5 versus 16, 18 and 22 months: *n* = 3). **c**–**f**, Flow cytometry data of CD90 T cells (**c**) (*n* = 12), dendritic cells (**d**) (*n* = 4), LY6C^+^ monocytes (**e**) (*n* = 12) and LY6G^+^ neutrophils (**f**) (*n* = 4) in the left ventricles of 3-, 16- and 22-month-old mice. **g**, CD68^+^ macrophages versus DAPI in mediastinal lymph nodes (MLN) of 3-, 16- and 22-month-old mice (*n* = 4). **h**, Water content (wet weight versus dry weight (%)) of hearts of 3-, 16-, 18-, 20- and 22-month-old male mice (3 months: *n* = 8 versus 16, 18 and 22 months: *n* = 4). **i**, Fibrinogen staining versus DAPI in hearts of 3-, 16- and 22-month-old mice (3 months and 22 months: *n* = 5 versus 16 months: *n* = 4). **j**,**k**, Quantification (**j**) and Congo red staining (**k**) in heart sections from young and old mouse hearts (*n* = 4 versus *n* = 3). **l**, Sirius red staining in heart sections from young and old male and female mouse hearts (*n* = 4). **m**, Heart weight versus tibia length (mg mm^−1^) ratio in 3- versus 18-month-old male mice (*n* = 6 versus *n* = 7). **n**–**p**, Echocardiography of young (3 months) versus old (18 months) male mice (*n* = 12), with graphs showing LV mass (**n**), diastolic function (**o**) (E/E′) and systolic function (**p**) (EF). Data are expressed as mean, and error bars indicate s.e.m. After passing normality tests, *P* values were calculated using a two-sided, unpaired *t*-test (**j**–**m** and **o**–**p**), unpaired, two-sided Mann–Whitney test (**n**), or ordinary one-way ANOVA with Tukey’s post hoc test (**a**–**i**). Source data

To assess whether reduced lymphatic density in the aged heart may have functional consequences, we determined pathophysiological processes linked to lymphatic dysfunction, including inflammation, edema formation and waste product accumulation. Time course analysis revealed that the decline in lymphatic vessel density, which started at 16 months of age (Fig. 2a), is followed by an accumulation of CD68^+^ macrophages and CD90^+^ T cells (pan *T* cell population), which reached statistical significance at 18 and 22 months of age, respectively (Fig. 2b,c). Dendritic cells also increased at 16 months (Fig. 2d), and LY6C^+^ monocytes and LY6G^+^ neutrophils showed progressive accumulation over time (Fig. 2e,f). To assess whether the accumulation of macrophages might indeed be a consequence of declined lymphatic function, we additionally measured macrophages in the draining lymph nodes. Indeed, mediastinal lymph nodes of aged mice (16 and 22 months) exhibited significantly reduced numbers of CD68^+^ macrophages, consistent with impaired macrophage clearance during aging (Fig. 2g).

Aging was further associated with increased edema formation beginning at 18 months (Fig. 2h), as well as the accumulation of plasma proteins such as fibrinogen starting at 16 months of age (Fig. 2i). We additionally observed an increase in amyloid deposition as assessed by Congo red staining (Fig. 2j,k). All these changes coincided or were present before the onset of cardiomyocyte hypertrophy, which was observed at 18 months, and fibrosis, which became substantial evident at 20 months (Fig. 2l,m), as previously reported^17^. Echocardiography further confirmed left ventricular hypertrophy and age-related diastolic dysfunction as indicated by an elevated E/E′ ratio (the ratio of early diastolic mitral inflow velocity to early diastolic mitral annular velocity, used as a surrogate for left ventricular filling pressure) (Fig. 2n,o). Systolic function was not changed, as determined by ejection fraction (EF) (Fig. 2p).

### Reducing lymphatic vessels induces cardiac inflammation and diastolic dysfunction

**Fig. 3:**
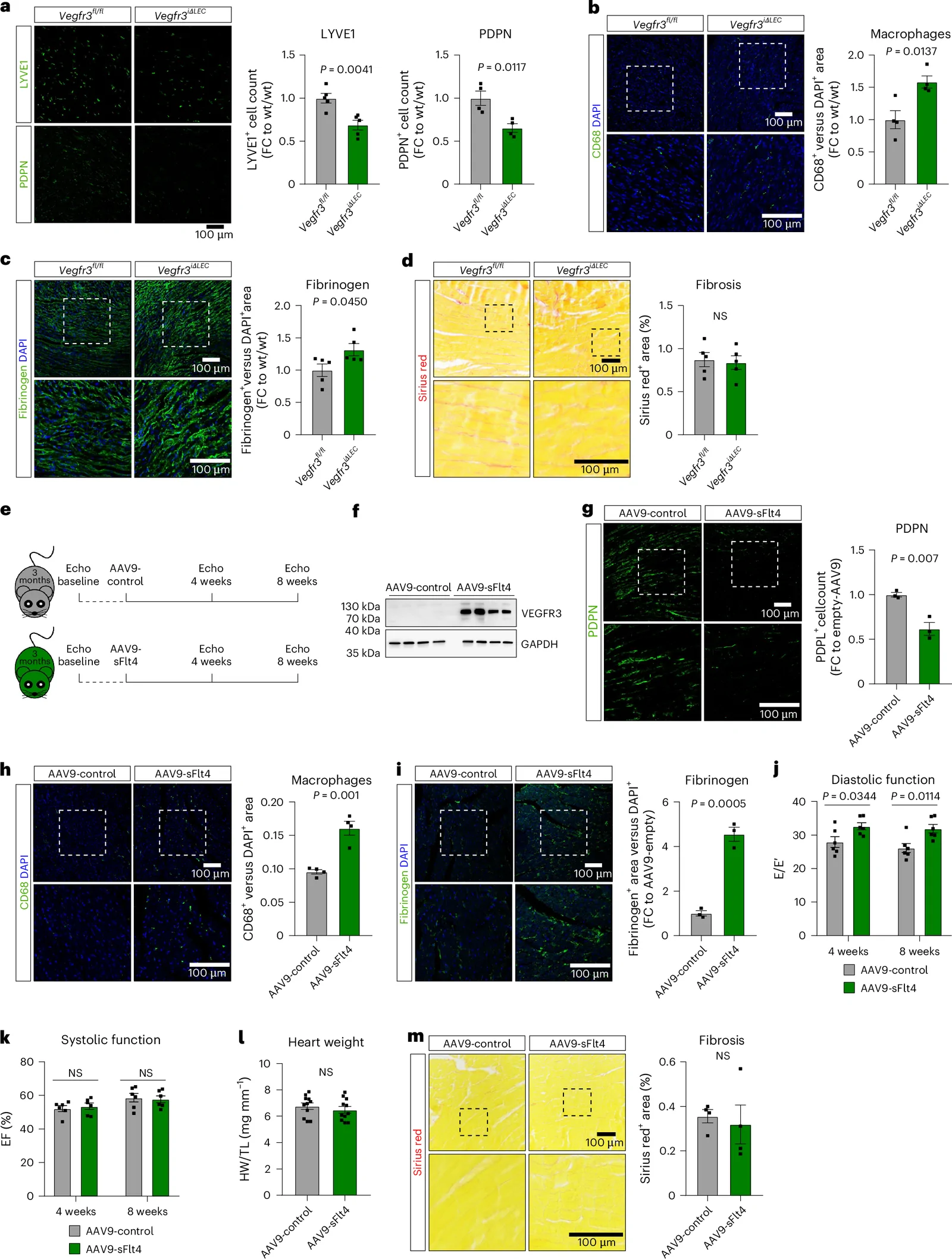
Disruption of cardiac lymphatics leads to macrophage and fibrinogen accumulation in young mouse hearts. **a**, Abundance of LYVE1^+^ (*n* = 5) and PDPN^+^ (*n* = 4) lymphatics in hearts of young adult *Vegfr3*^*fl/fl*^ versus *Vegfr3*^*iΔLEC*^ female mice, 4 weeks after tamoxifen administration. **b**, CD68^+^ macrophages versus DAPI in hearts of young adult *Vegfr3*^*fl/fl*^ versus *Vegfr3*^*iΔLEC*^ mice, 4 weeks after tamoxifen administration (*n* = 4). **c**, Fibrinogen versus DAPI in hearts of young adult *Vegfr3*^*fl/fl*^ versus *Vegfr3*^*iΔLEC*^ female mice, 4 weeks after tamoxifen administration (*n* = 5). **d**, Sirius red staining in hearts of young adult *Vegfr3*^*fl/fl*^ versus *Vegfr3*^*iΔLEC*^ mice, 4 weeks after tamoxifen administration (*n* = 5). **e**, A schematic illustration of 3-month-old young male mice receiving AAV9-sFlt4 or AAV9-control injection. Heart function was monitored before and 4 and 8 weeks after AAV9 injection. **f**, A representative WB of VEGFR3 and GAPDH in heart lysates of AAV9-control and AAV9-sFlt4-treated mice (*n* = 4). **g**, Abundance of PDPN^+^ lymphatics in hearts of AAV9-control and AAV9-sFlt4-treated mice, 8 weeks after AAV9 administration (*n* = 3). **h**, CD68^+^ macrophages versus DAPI in hearts of AAV9-control and AAV9-sFlt4-treated mice, 8 weeks after AAV9 administration (*n* = 4). **i**, Fibrinogen versus DAPI in hearts of AAV9-control and AAV9-sFlt4-treated mice, 8 weeks after AAV9 administration (*n* = 3). **j**,**k**, Echocardiography monitoring of AAV9-control and AAV9-sFlt4-treated mice 4 and 8 weeks after AAV9 injection (*n* = 6), with the graphs showing diastolic function (**j**) (E/E′) and systolic function (**k**) as EF (%). **l**, Heart weight (heart weight (HW) versus tibia length (TL)) in AAV9-control and AAV9-sFlt4-treated mice (*n* = 11) **m**, The mean cardiomyocyte size in the left ventricle of AAV9-control and AAV9-sFlt4-treated mice as determined by WGA staining (*n* = 4). **n**, Sirius red staining in hearts of AAV9-control and AAV9-sFlt4-treated mice, 8 weeks after tamoxifen administration (*n* = 4). Data are expressed as mean, and error bars indicate s.e.m. After passing normality tests, *P* values were calculated using a two-sided, unpaired *t*-test (**a**–**d** and **g**–**m**). NS, not significant; wt/wt, weight/weight. Source data

**Extended Data Fig. 2:**
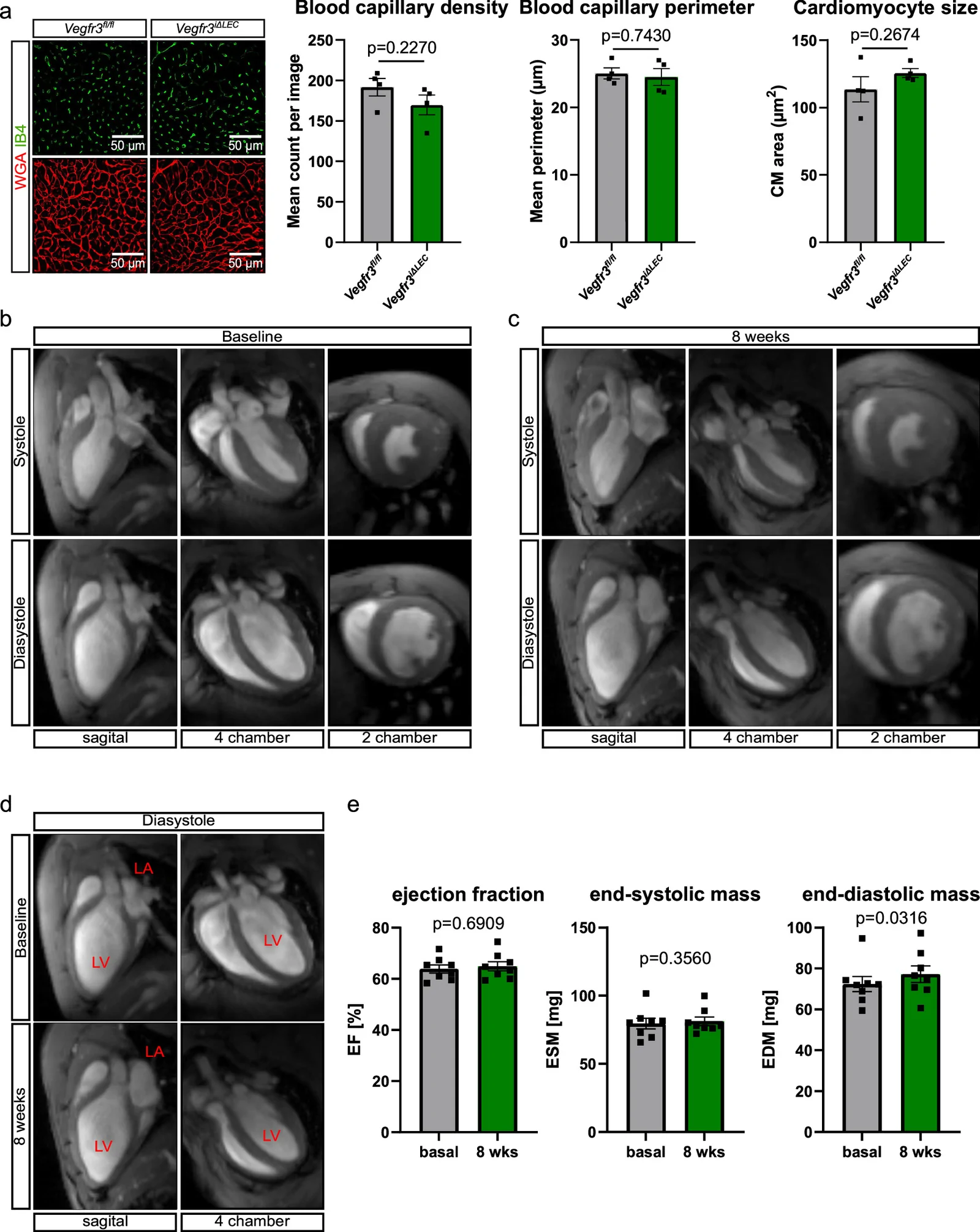
Cardiac hypertrophy is not affected by *Vegfr3* deletion. **a** Cardiac hypertrophy was assessed by WGA staining and capillary density was assessed by IB4 staining in heart sections from Vegfr3^iΔLEC^ versus Cre-negative (Vegfr3^fl/fl^) litter mates (n = 4). **b-e** Magnet resonance imaging (MRI) of Vegfr3^iΔLEC^ mice before (b) and eight weeks after tamoxifen injection (c). Shown are scans at systole and diastole in sagittal, 4-chamber and 2-chamber view. Comparison between baseline and eight weeks post tamoxifen are shown in (d) while e shows the quantification of ejection fraction, end-systolic and end-diastolic mass (n = 8). All mice were female. Data are expressed as mean and error bars indicate standard error of the mean. After passing normality tests, p-values were calculated using two-sided, unpaired T-test (a, e). Source data

**Extended Data Fig. 3:**
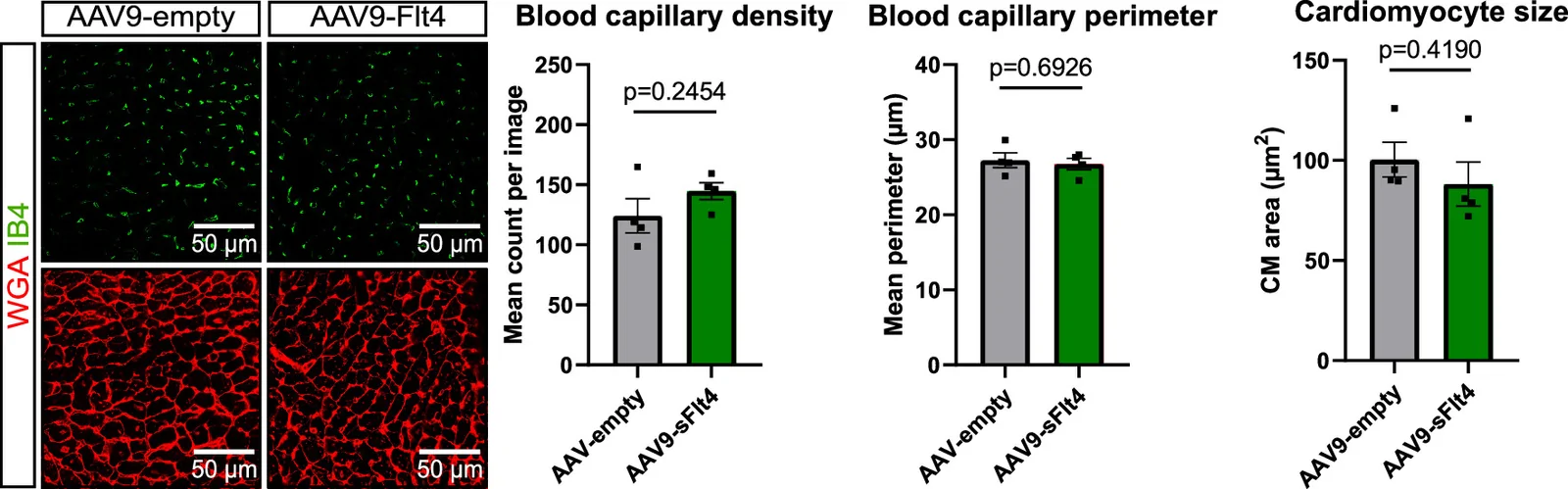
Cardiac hypertrophy is not affected by soluble VEGFR3 induction. Cardiac hypertrophy was assessed by WGA (red) staining and capillary density was assessed by IB4 (green) staining in heart sections from young mice eight weeks after soluble VEGFR3 overexpression (AAV9-sFlt4) (n = 4). All mice were male. Data are expressed as mean and error bars indicate standard error of the mean. After passing normality tests, p-values were calculated using two-sided, unpaired T-test. Source data

To assess whether a direct reduction in lymphatic vessels can induce age-related cardiac phenotypes, we conditionally deleted *Flt4*, which encodes the essential lymphatic receptor VEGFR3, in lymphatic ECs (LymphECs) of young mice (referred to as *Vegfr3*^iΔLEC^). Cre-negative littermates served as controls (referred to as *Vegfr3*^*fl/fl*^). At 1 month after deletion, we observed a significant decline of LYVE-1^+^ and PDPN^+^ lymphatic vessel density in the heart (Fig. 3a), which was associated with an increase of CD68^+^ macrophages (Fig. 3b) and fibrinogen (Fig. 3c). However, we did not observe increased fibrosis at this time point (Fig. 3d). Blood capillary density, perimeter and cardiomyocyte size were also not affected (Extended Data Fig. 2a). Functional assessments by magnet resonance imaging suggests diastolic dysfunction as shown by elevated end-diastolic mass and enlarged left atrial size, while systolic function was preserved (Extended Data Fig. 2b–e). Similar findings were observed when overexpressing the soluble form of *Flt4* (sFlt4), which acts as a trap for the VEGFR3 ligands VEGFC and VEGFD and inhibits their signaling, with adeno-associated virus serotype 9 (AAV9-sFlt4) (Fig. 3e,f). AAV9-sFlt4-treated mice showed a similar decline in lymphatic vessel density (Fig. 3g), which was accompanied by increased macrophage abundance (Fig. 3h) and fibrinogen accumulation (Fig. 3i). Echocardiographic analysis further revealed a significant increase in diastolic dysfunction (Fig. 3j). Systolic heart function, heart weight and cardiomyocyte size, fibrosis, and blood vessel capillaries were, however, not altered (Fig. 3k–m and Extended Data Fig. 3). Together, these data demonstrate that lymphatic vessel reduction exacerbates accumulation of inflammatory cells, promotes fibrinogen accumulation and induces diastolic dysfunction.

### Aging induces expression of nuclear interleukin-33

**Fig. 4:**
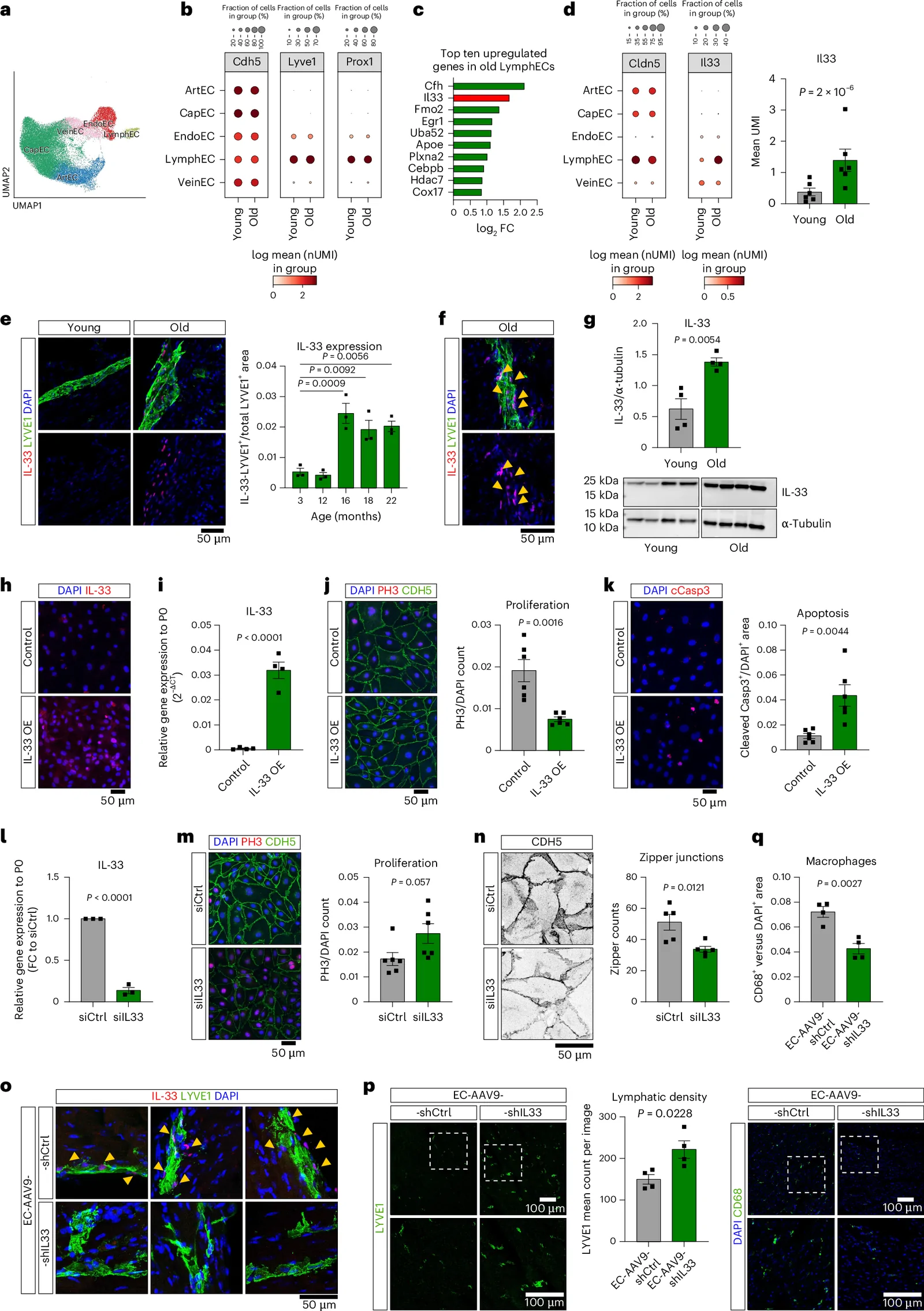
Age-related IL-33 expressions underlies cardiac lymphatic decline. **a**,**b**, The endothelial clusters (**a**) with lymphatic-specific marker expression (**b**). **c**, The top ten upregulated genes in aged lymphatics. **d**, The expression of Cldn5 and Il33 in the respective EC cluster and the mean unique molecular identifier count per sample in the lymphatic cluster (*n* = 6). The diagrams in **a**–**d** show scRNA-seq data of isolated ECs from young versus old male mouse hearts. Data were integrated into published data from young and old mouse hearts^18,19^ to increase the statistical power (total sample size: *n* = 6 per group). **e**,**f**, IL-33 expression in 3-, 12-, 16-, 18- and 22-month-old male mouse hearts (*n* = 3) with LYVE1 and DAPI as the counterstain (**e**); panel **f** shows the nuclear localization of IL-33 (arrowheads). **g**, A WB of whole heart lysates of young versus old male mouse hearts (*n* = 4) against IL-33 and alpha-tubulin. **h**,**i**, Lentiviral overexpression of IL-33 in HDLECs is shown: IL-33 expression by immunofluorescence staining (IL-33 and DAPI) (**h**) and quantitative polymerase chain reaction with reverse transcription (RT–qPCR) (**i**) (*n* = 4 per group; each *n* represents an independent experimental replicate). **j**,**k**, Overexpression of IL-33 in HDLECs, with proliferation shown via PH3 staining (**j**) with CDH5 and DAPI serving as counterstaining (*n* = 6 per group; each data point represents the mean PH3 signal of an independent experimental replicate) and apoptosis (**k**) as assessed by cleaved caspase-3 (cCasp3) staining with DAPI serving as counterstaining (*n* = 6). **l**–**n**, siRNA-mediated IL-33 knockdown in HDLECs as shown by RT–qPCR (**l**) (*n* = 3; each data point represents an independent experimental replicate), PH3 staining (**m**) in HDLECs with CDH5 and DAPI serving as counterstaining (*n* = 6; each data point represents the mean PH3 signal of an independent experimental replicate) and the quantification of zipper junctions (**n**) in these cells (*n* = 5; each data point represents an independent experimental replicate). **o**, Representative images of IL-33 staining in cardiac lymphatics. DAPI was used as the counterstain. **p**, LYVE1 staining to quantify lymphatic density. **q**, Macrophage density by CD68 staining versus DAPI. In **o**–**q**, modified AAV9 were intravenously injected into 18-month-old male mice to induce the expression of shRNA targeting *Il33* (EC-AAV9-shIL33). A nontargeting shRNA served as the control (EC-AAV9-shCtrl). At 4 weeks after injection, hearts were assessed histologically (*n* = 4). Data are expressed as mean, and error bars indicate s.e.m. After passing normality tests, *P* values were calculated using a two-sided, unpaired *t*-test (**g**–**q**) or ordinary one-way ANOVA with Tukey’s post hoc test (**e**). The *P* value shown in **d** was calculated using the MAST test. ArtEC, arterial ECs; CapEC, capillary ECs; EndoEC, endocardial ECs; nUMI, number of unique molecular identifiers; OE, overexpression; VeinEC, venous ECs; siCtrl, non-targeting control siRNA; siIL33, siRNA targeting *IL33*-mRNA; shCtrl, non-targeting shRNA; shIL33, shRNA targeting *Il33*-mRNA. Source data

**Extended Data Fig. 4:**
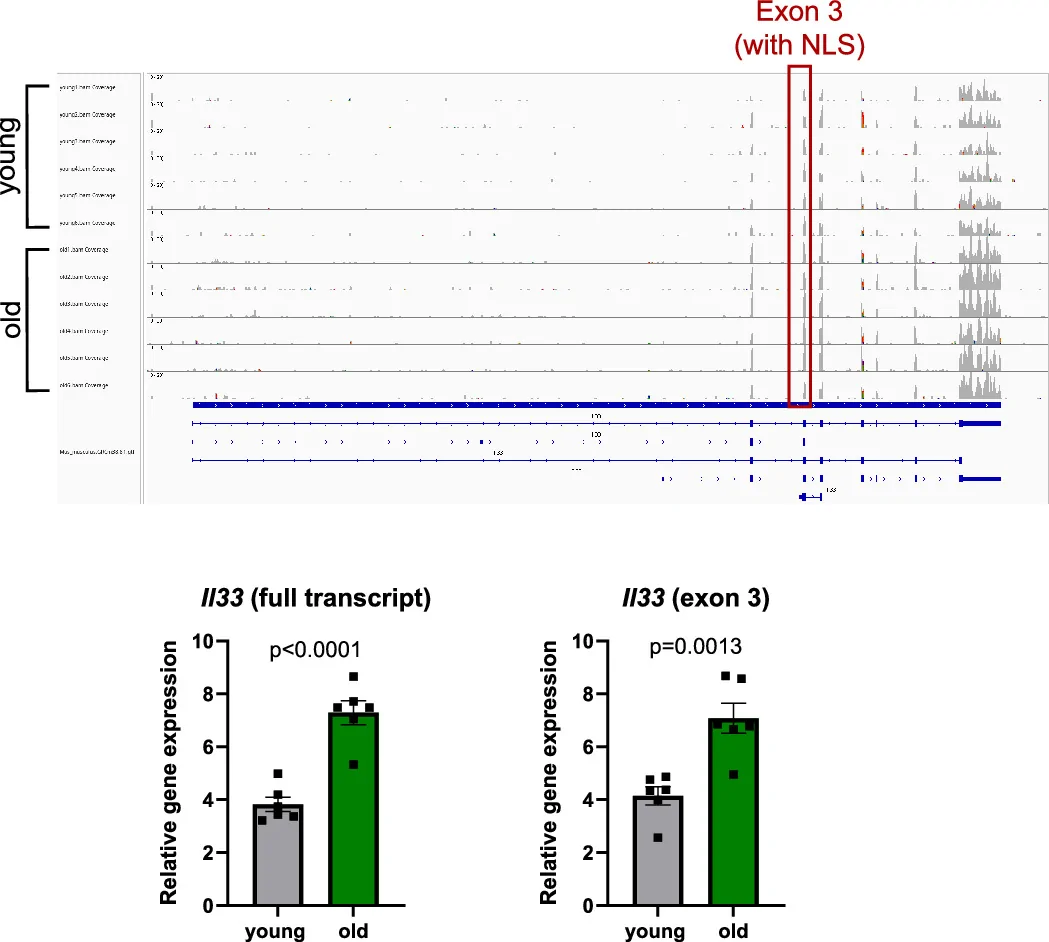
IL33 regulation in aged hearts. Expression of *Il33* (full transcript expression versus exon 3 expression) by bulk RNA sequencing in isolated endothelial cells from young and old mouse hearts (n = 6, data taken from Wagner et al., Science ^2^). Source data

To determine what drives lymphatic decline in aging, we performed single-cell RNA sequencing (scRNA-seq) on isolated Cdh5^+^ ECs from young and aged mouse hearts. To enrich for lymphatic vessels, we integrated this dataset to previously published scRNA-seq data from young and old hearts^18,19^ (Fig. 4a). The endothelial clusters were subclustered, and the LymphEC cluster showed the characteristic expression of lymphatic markers *Cdh5*, *Lyve1* and *Prox1* (Fig. 4b). Overall, 49 genes were significantly upregulated, while 2 were downregulated in aged LymphECs (Fig. 4c and Extended Data Table 1). The downregulated genes include *Cldn5*, which is mainly known for its role in maintaining the vascular barrier (Fig. 4d). Among the top upregulated genes, we found complement factor H (CFH), which is a key regulator of the alternative pathway of the complement system, and the cytokine interleukin-33 (IL-33). *Il33* expression was specifically enriched in aged LymphECs (Fig. 4d). Immunostaining confirmed the upregulation of IL-33 protein levels in LymphECs at 16 months (Fig. 4e), the time point at which lymphatic vessel density begins to decline during aging (Fig. 2a). IL-33 is a cytokine belonging to the IL-1 family, which regulates fibrosis^20^, inflammation^21,22^ and angiogenesis^23^. In addition, IL-33 has a dual function: it acts both as a classic extracellular cytokine and as a higher-weight nuclear factor. This higher-molecular-weight isoform, often referred to as nuclear IL-33 (nIL-33), was shown to interfere with NF-kB and act as chromatin regulator^23^. Interestingly, RNA sequencing (RNA-seq) analysis of isolated cardiac ECs^2^ revealed elevated expression of the nuclear *Il33* isoform, which contains the nuclear localization sequence encoded by exon 3 in samples from aged mouse hearts (Extended Data Fig. 4). Consistently, immunostainings predominantly showed an accumulation of nIL-33 in lymphatics of the aging heart (Fig. 4f). Western blots (WBs) confirmed the upregulation of the higher-molecular-weight nuclear isoform in the aging heart (Fig. 4g).

**Extended Data Fig. 5:**
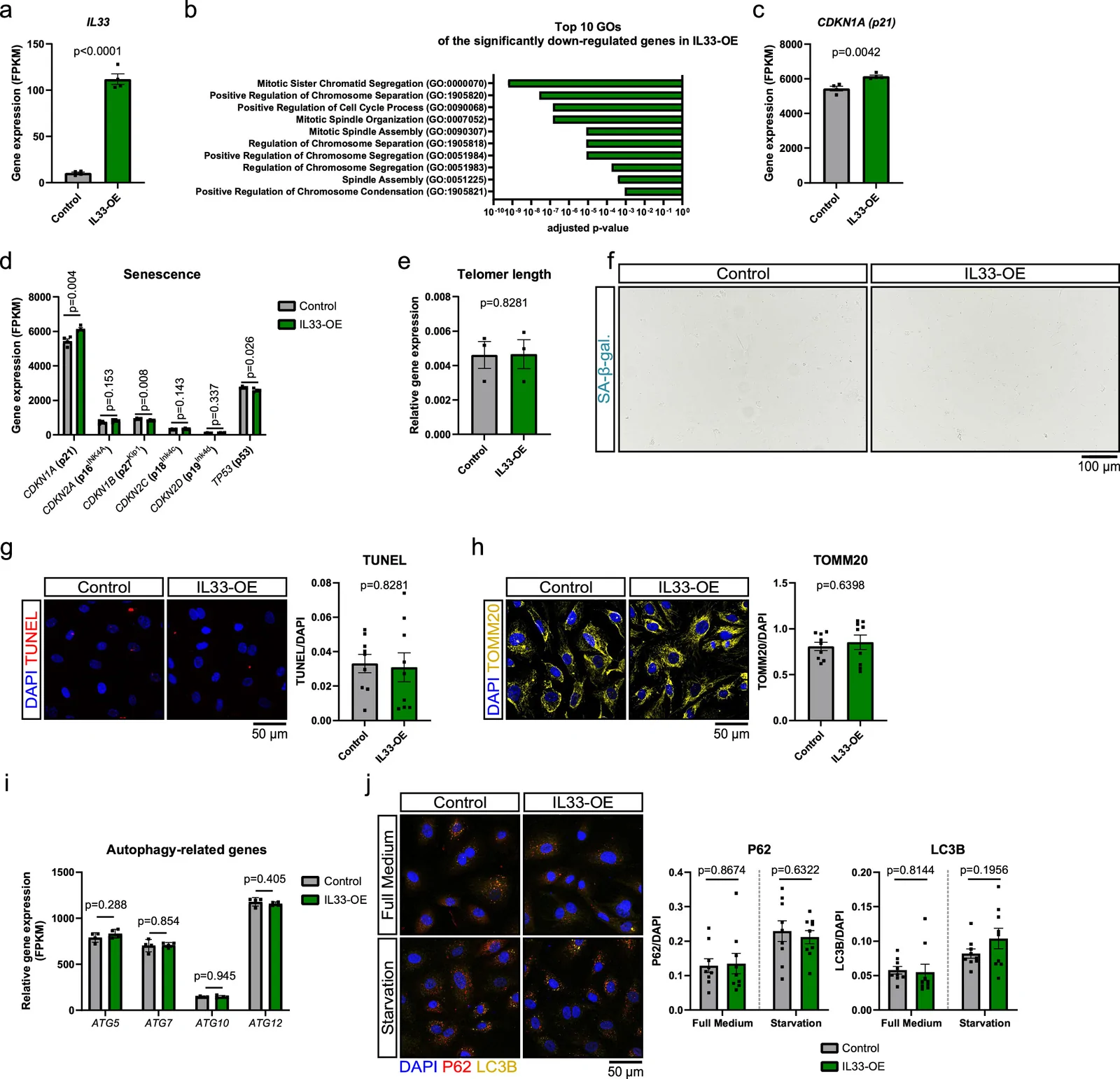
Assessing hallmarks of ageing in IL33-OE HDLECs. **a-c** *IL33* was overexpressed via lentiviruses in HDLECs. A control virus served as control (n = 4 per group). RNA was extracted and mRNA sequencing was performed **a** *IL33* expression in control and IL33 overexpressing HDLECs (n = 4). **b** Gene ontology analyses of genes that are significantly down-regulated upon *IL33* overexpression. **c** *CDKN1A* expression in control and IL33 overexpressing HDLECs (n = 4). **d** Bulk RNA sequencing of IL33-overexpressing (OE) vs. control HDLECs (n = 4) showing the expression of senescence marker genes. **e** Quantification of telomer length by quantitative PCR in IL33-overexpressing (OE) vs. control HDLECs (n = 3). **f** Representative images of senescence-associated β-galactosidase staining in control vs. IL33-OE HDLECs (n = 3). **g** TUNEL staining in IL33-overexpressing (OE) vs. control HDLECs (n = 9). TUNEL area was quantified and normalized to DAPI area. **h** TOMM20 staining in IL33-overexpressing (OE) vs. control HDLECs (n = 9). TOMM20 area was quantified and normalized to DAPI area. **i** Bulk RNA sequencing of IL33-overexpressing (OE) vs. control HDLECs (n = 4) showing the expression of autophagy-related genes. **j** P62 and LC3B staining in IL33-overexpressing (OE) vs. control HDLECs in fully supplemented medium and starvation medium (n = 9). P62 and LC3B area was quantified and normalized to DAPI area respectively. Data normally distributed and p-values were calculated using an unpaired, two-sided T-test (a, c, d, e, g-j). Source data

While the secreted form of IL-33 was shown to induce lymphangiogenesis^23^, the function of nIL-33 in lymphatics has not been assessed. Therefore, we overexpressed nIL-33 in human dermal LymphECs (HDLECs) in vitro (Fig. 4h,i). Overexpression of nIL-33 reduced proliferation and induced apoptosis of HDLECs (Fig. 4j,k), whereas silencing of *Il33* tended to increase cell number (Fig. 4l,m) and reduced zipper formation (Fig. 4n). In line with this, RNA-seq of *IL33*-overexpressing HDLECs revealed the downregulation of mitosis-related genes such as *CDKN1A* after *IL33* induction (Extended Data Fig. 5a–c). Further hallmarks of cellular aging, including senescence, DNA damage, mitochondrial abundance and autophagy were not changed upon *IL33* overexpression (Extended Data Fig. 5d–j). Silencing IL-33 in lymphatic vessels of aged mouse hearts by using AAV9 encoding for *Il33*-targeting short hairpin RNAs (shRNAs) rescued lymphatic density and reduced macrophage abundance (Fig. 4o–q). Together, these data suggest that age-induced augmentation of nIL-33 may contribute to the decline and structural alterations of the lymphatic vasculature during aging.

### Interferon gamma induces IL-33 expression

**Fig. 5:**
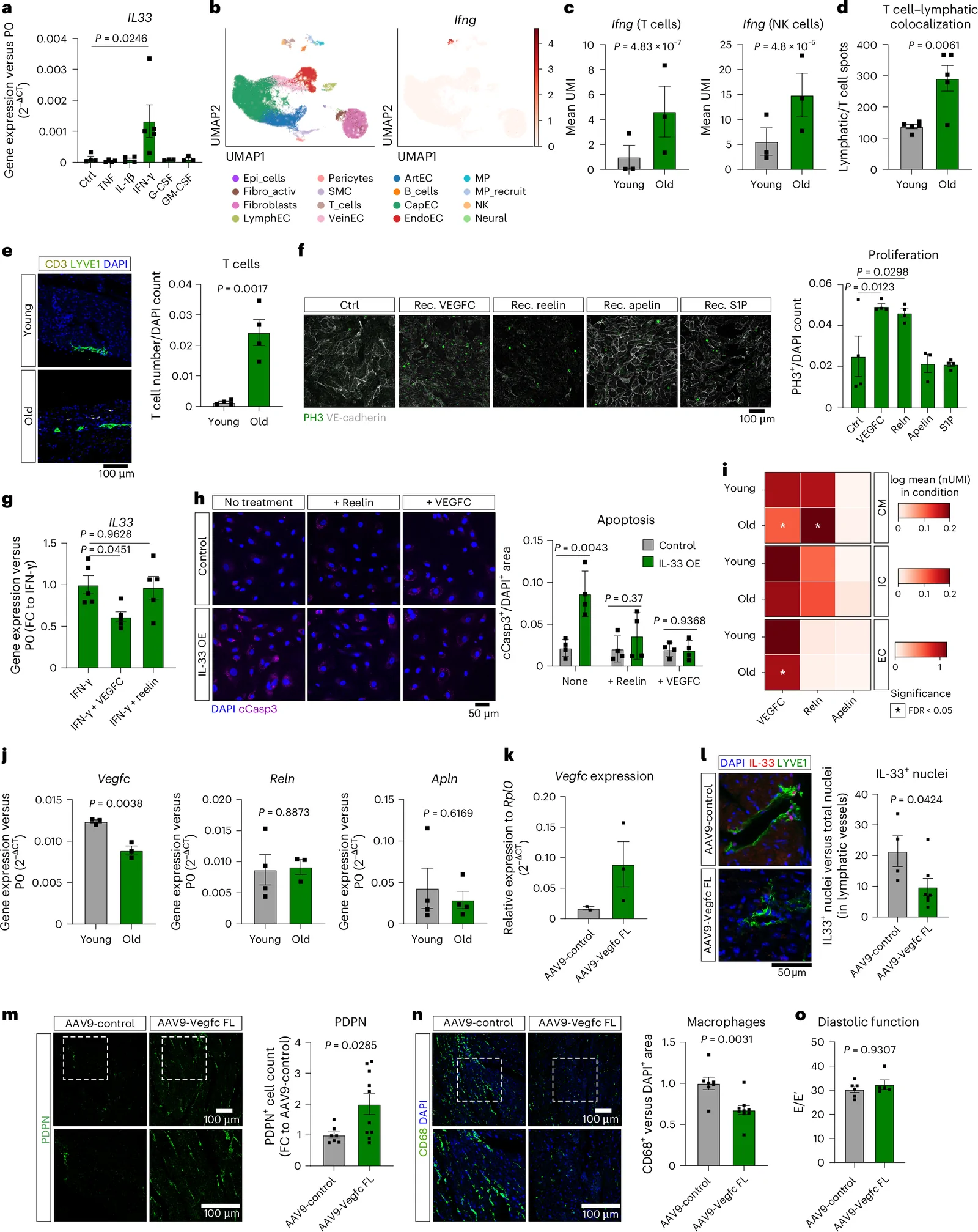
VEGFC overexpression restores cardiac lymphatic density and reduces macrophages in aged hearts. **a**, HDLECs were treated with recombinant IFN-γ (30 ng ml^−1^, *n* = 5), TNF (10 ng ml^−1^, *n* = 4), IL-1β (100 ng ml^−1^, *n* = 4), G-CSF (100 ng ml^−1^, *n* = 3) and GM-CSF (50 ng ml^−1^, *n* = 3) for 24 h. IL-33 expression was determined by RT–qPCR. Each data point represents an independent experimental replicate. **b**, The expression of *Ifng*. **c**, The expression of *Ifng* in the T cell and NK cell cluster. In **b** and **c**, scRNA-seq data of isolated ECs from young versus old male mouse hearts are shown. Data were integrated into published data from young and old mouse hearts^18,19^ to increase the statistical power (total sample size: *n* = 6 per group). **d**, T cell and lymphatic colocalization in spatial transcriptomics data from young versus old female mouse hearts (*n* = 5 per group). Data were obtained from Rodriguez Morales et al.^24^. **e**, CD3 staining versus LYVE1 and DAPI in heart sections from young (3 months) and old (20 months) male mice (*n* = 4). **f**, HDLECs incubated with recombinant (rec.) VEGFC (100 ng ml^−1^, *n* = 4), reelin (100 ng ml^−1^, *n* = 4), apelin (100 ng ml^−1^, *n* = 3) or S1P (100 ng ml^−1^, *n* = 4) for 48 h. BSA treatment served as the control condition (*n* = 4). Proliferation was determined by PH3 staining. CDH served as the counterstain. Each data point represents the mean PH3 signal of an independent experimental replicate. **g**, HDLECs were incubated either with recombinant IFN-γ (30 ng ml^−1^) or with a combination of IFN-γ and recombinant VEGFC or reelin (100 ng ml^−1^ each) for 24 h (*n* = 5; each data point represents an independent experimental replicate). IL-33 expression was quantified by RT–qPCR. **h**, HDLECs after lentiviral overexpression of *IL33* or a nontargeting sequence (control) were treated with recombinant VEGFC or reelin (100 ng ml^−1^ each) for 24 h. Apoptosis was determined by cCasp3 staining versus DAPI. The use of no recombinant protein (none) served as the negative control (*n* = 4; each data point represents the mean cCasp3 signal of an independent experimental replicate). **i**, Expression of *Vegfc*, *Reln* and *Apln* in ECs, immune cells (IC) and cardiomyocytes (CM) in young and old mouse hearts. Single-nuclei RNA data were obtained from Vidal et al.^53^. **j**, Expression of *Vegfc* (*n* = 3), *Reln* (*n* = 4 versus *n* = 3) and *Apln* (*n* = 4) in whole hearts from young (3 months) and old (20 months) mice. Gene expression was determined by RT–qPCR versus *Rpl0*. **k**, *Vegfc* mRNA expression in hearts from AAV9-control and AAV9-Vegfc full-length (FL)-treated mice (*n* = 2 versus *n* = 3). **l**, IL-33^+^ nuclei were quantified (%) per lymphatic vessel (*n* = 4 versus *n* = 7). **m**, Abundance of PDPN^+^ lymphatics in hearts of AAV9-control and AAV9-Vegfc FL-treated mice, 8 weeks after AAV9 administration (*n* = 3). **n**, CD68^+^ macrophages versus DAPI in hearts of AAV9-control and AAV9-Vegfc FL-treated mice, 8 weeks after AAV9 administration (*n* = 7 versus *n* = 9). In **k**–**n**, AAV9-Vegfc FL or AAV9-control were i.p. injected into 18-month-old male mice. At 8 weeks after injection, hearts were transcriptomically and histologically assessed. **o**, Diastolic function (E/E′) assessed by echocardiography in aged male AAV9-control and AAV9-Vegfc FL-treated mice, 8 weeks after AAV9 administration (*n* = 6 versus *n* = 5). Data are expressed as mean, and error bars indicate s.e.m. After passing normality tests, *P* values were calculated using a two-sided, unpaired *t*-test (**d**, **e**, **h**, **j** and **m**–**o**) or ordinary one-way ANOVA with a post hoc Dunnett’s test (**a**, **f** and **g**). Data shown in **o** were not normally distributed and were assessed using a two-sided, unpaired Mann–Whitney test (**l**). *P* values shown in **c** were calculated using the MAST test. FDR, false discovery rate. Source data

Next, we sought to identify the upstream mechanisms driving age-related *IL33* expression in cardiac lymphatics. Given that cardiac aging is characterized by persistent low-grade inflammation, commonly termed ‘inflammaging’, we examined whether proinflammatory cytokines—tumor-necrosis factor (TNF), interleukin-1β (IL-1β), interferon gamma (IFN-γ), granulocyte colony-stimulating factor (G-CSF) and granulocyte–macrophage colony-stimulating factor (GM-CSF)—could induce *IL33* expression in HDLECs in vitro. Among the tested cytokines, only IFN-γ significantly upregulated *IL33* in HDLECs after 24 h of treatment (Fig. 5a). Supporting a role for IFN-γ in driving IL-33 upregulation in vivo, *Ifng* gene expression was elevated in aged hearts and predominantly localized to T cells and natural killer (NK) cells (Fig. 5b,c).

**Extended Data Fig. 6:**
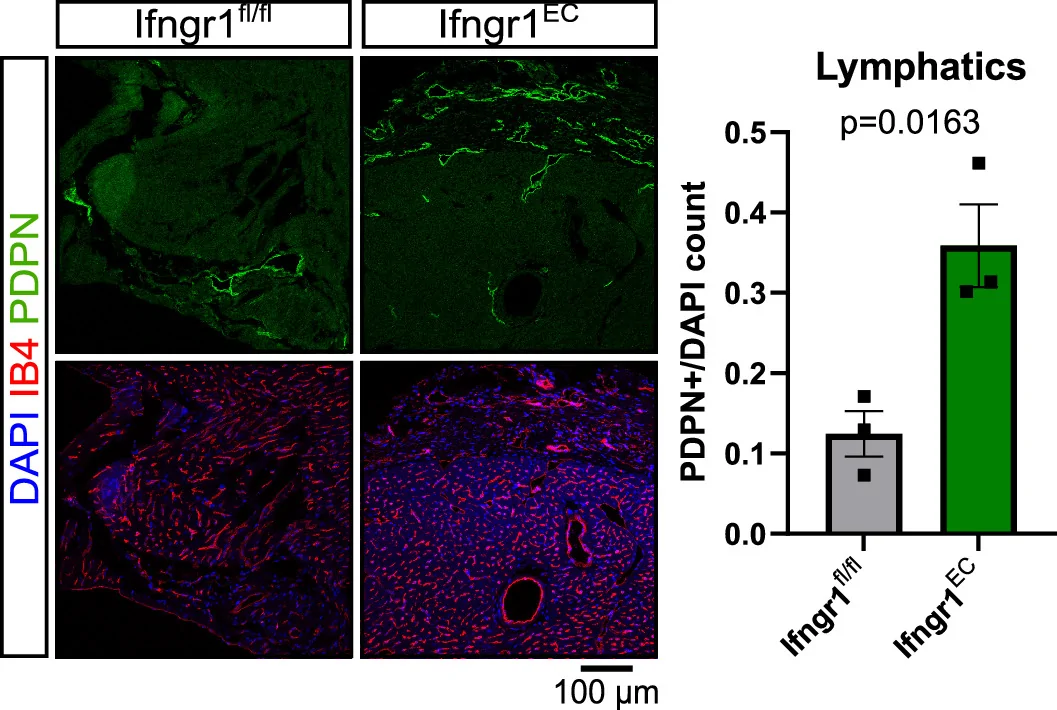
Interferon receptor deletion rescues lymphatic in failing hearts. Podoplanin staining in male mouse hearts 28 days after permanent LAD Ligation versus sham (n = 3). IB4 and DAPI served as counterstain. Sections are taken from previously published data (Tombor et al., Circulation research 2025). Data are expressed as mean and error bars indicate standard error of the mean. After passing normality tests, p-values were calculated using two-sided, unpaired T-test. Source data

To determine whether T cells are positioned in proximity to lymphatic vessels and may thus engage in crosstalk with lymphatic endothelium, we analyzed spatial transcriptomics datasets from young and old mouse hearts^24^. These data revealed a close spatial association between T cells and lymphatic vessels in aged hearts (Fig. 5d). Immunofluorescence analysis corroborated the age-related accumulation of T cells (Figs. 2c and 5e) and their close spatial alignment with lymphatics (Fig. 5e). Collectively, these observations are consistent with a model in which cardiac T cells increasingly produce IFN-γ with age, thereby potentially promoting IL-33 expression and contributing to lymphatic dysfunction. Notably, EC-specific deletion of IFN-γ receptor 1 increased lymphatic density in a non-aging heart failure model (Extended Data Fig. 6), supporting a broader role of IFN-γ signaling in limiting cardiac lymphatic growth.

**Extended Data Fig. 7:**
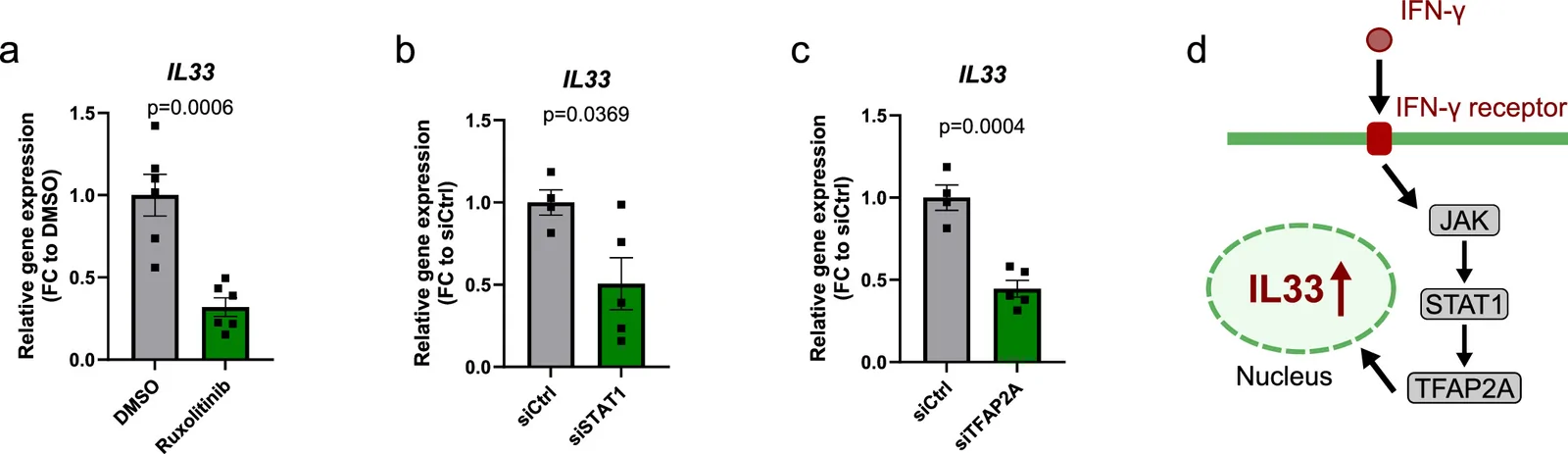
Interferon induces IL33 via JAK-STAT1-TFAP2A. **a** HDLECs were treated with 5 µm ruxolitinib and 100 nM recombinant IFN-γ for 24 h. *IL33* expression was determined by RT-qPCR versus RPL0 as housekeeper (n = 6). **b** *STAT1* was silenced by siRNA transfection in HDLECs. Two days after transfection, cells were treated for 24 h with recombinant IFN-γ (100 nM). *IL33* expression was determined by RT-qPCR versus RPL0 as housekeeper (n = 4 vs. n = 5). **c** *TFAP2A* was silenced by siRNA transfection in HDLECs. Two days after transfection, cells were treated for 24 h with recombinant IFN-γ (100 nM). *IL33* expression was determined by RT-qPCR versus RPL0 as housekeeper (n = 4 vs. n = 5). **d** Schematic representation of the putative pathway. P-value was calculated using the unpaired, student’s T-test (a-c). Source data

To further assess the molecular mechanism underlying IFN-γ signaling in LymphECs, we focused on the canonical Janus kinase (JAK)/STAT1 pathway, which was shown to induce IL-33 expression in epithelial cells^25^. To determine whether this pathway also regulates IL-33 expression in LymphECs, we inhibited JAK by the inhibitor ruxolitinib. JAK inhibition suppressed IFN-γ-induced *IL33* expression (Extended Data Fig. 7a). Likewise, small interfering RNA (siRNA)-mediated *STAT1* silencing reduced IFN-γ-induced *IL33* expression in HDLECs (Extended Data Fig. 7b).

To elucidate how IFN-γ increases the splicing of nIL-33, we further assessed the contribution of transcription factor AP-2 alpha (TFAP2A). TFAP2A acts downstream of STAT1^26^, which was shown to indirectly regulate *IL33* splicing, resulting in the inclusion of exon 3, which contains the nuclear localization signal^27^. Indeed, siRNA-mediated *TFAP2A* silencing decreased *IL33* expression in IFN-γ-treated HDLECs (Extended Data Fig. 7c).

Collectively, these data indicate that IFN-γ activates the JAK/STAT1 pathway to promote IL-33 expression in LymphECs. STAT1 may further induce TFAP2A, thereby promoting the inclusion of exon 3 (nIL-33) in IL-33 mRNA (Extended Data Fig. 7d).

### VEGFC reduces macrophage accumulation but does not preserve cardiac diastolic function

Finally, we aimed to modulate *Il33* expression in lymphatic vessels of aged mice. To this end, we screened lymphangiogenic growth factors, including VEGFC^28^, reelin^29^, apelin^30^ and sphingosine-1-phosphate (S1P)^30^, hypothesizing that some may not only promote lymphatic vessel growth but also suppress *Il33* expression. HDLECs were treated with recombinant VEGFC, reelin, apelin or S1P, and proliferation was assessed by phospho-histone-3 (PH3) staining. Among the tested factors, only recombinant VEGFC and reelin significantly stimulated HDLEC proliferation (Fig. 5f).

Next, we investigated whether VEGFC or reelin could counteract IFN-γ-induced IL-33 expression. Notably, only recombinant VEGFC attenuated IFN-γ-driven *IL33* expression (Fig. 5g). Moreover, VEGFC more effectively reduced apoptosis in HDLECs following lentiviral IL-33 overexpression compared with reelin (Fig. 5h). Interestingly, of all tested growth factors, only *Vegfc* expression was significantly decreased in cardiomyocytes and endothelial clusters and tended to be reduced in immune cells in old mouse hearts, while *Apln* and *Reln* expression were not reduced (Fig. 5i). RT–qPCR confirmed age-related *Vegfc* decline in total heart samples from young and aged mice (Fig. 5j). Together, these findings suggest that VEGFC deficiency may contribute to age-related lymphatic dysfunction and that restoring VEGFC signaling may mitigate IL-33-mediated impairment of cardiac lymphatics.

**Extended Data Fig. 8:**
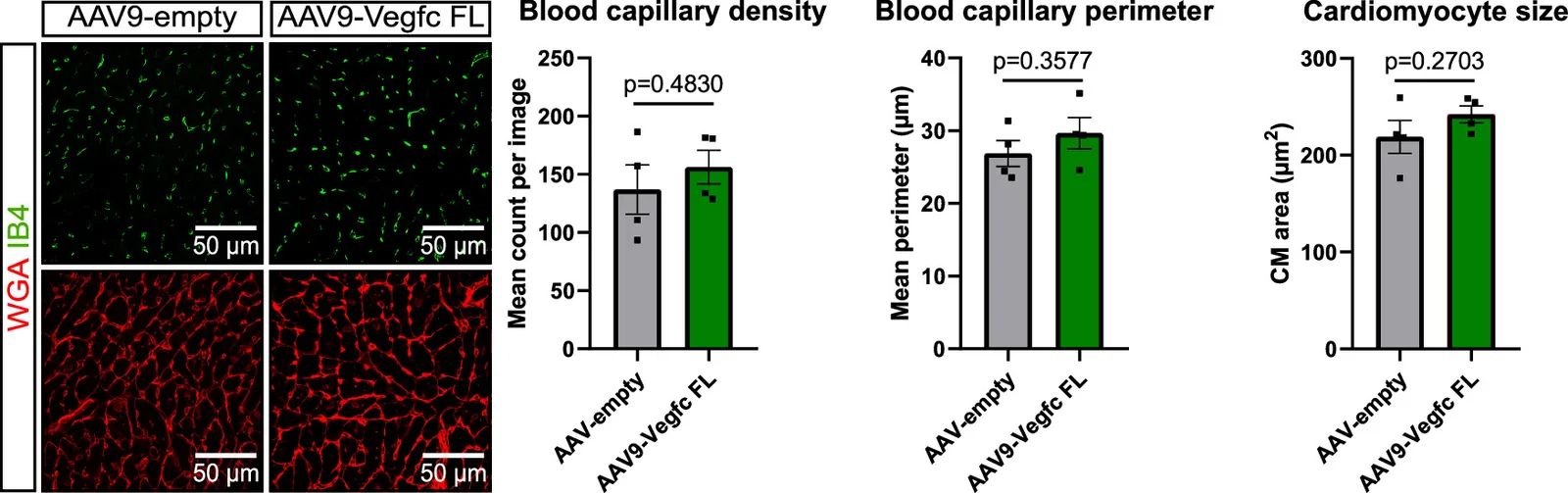
Cardiac hypertrophy is not affected by *Vegfc* induction. Cardiac hypertrophy was assessed by WGA staining and capillary density was assessed by IB4 staining in heart sections from old mice eight weeks after overexpression of VEGFC (AAV9-Vegfc FL) (n = 4). All mice were male. Data are expressed as mean and error bars indicate standard error of the mean. After passing normality tests, p-values were calculated using two-sided, unpaired T-test. Graphical summary. Advanced age is associated with reduced lymphatic density in the left ventricle and increased abundance of zipper-like junctions. The age-related reduction in lymphatic density is associated with impaired cardiac draining and increased abundance of immune cells, plasma proteins such as fibrinogen and interstitial fluid. Interferon-gamma (IFN-γ) was found to induce nuclear interleukin-33 (IL33) in lymphatic endothelial cells via the JAK-STAT1-TFAP2 pathway. Overexpressing IL33 in lymphatic endothelial cells induced apoptosis and reduced lymphatic endothelial proliferation. Source data

To test this hypothesis in vivo, we overexpressed the full-length *Vegfc* isoform in 18-month-old mice and assessed its effects on cardiac lymphatic structure and function (Fig. 5k). Indeed, overexpression of full-length *Vegfc* (AAV9-Vegfc FL) decreased IL-33 expression in aged cardiac lymphatics and increased lymphatic vessel density in the aging heart (Fig. 5l,m). Moreover, AAV9-Vegfc FL treatment reduced age-associated accumulation of macrophages in the heart (Fig. 5n) but did not alter blood capillary density or cardiomyocyte size (Extended Data Fig. 8). Age-related diastolic dysfunction was not attenuated either (Fig. 5o). These data suggest that VEGFC rescues the age-associated decline in lymphatic vessel density by repressing *Il33* expression and rescues the accumulation of inflammatory cells.

## Discussion

**Extended Data Fig. 9:**
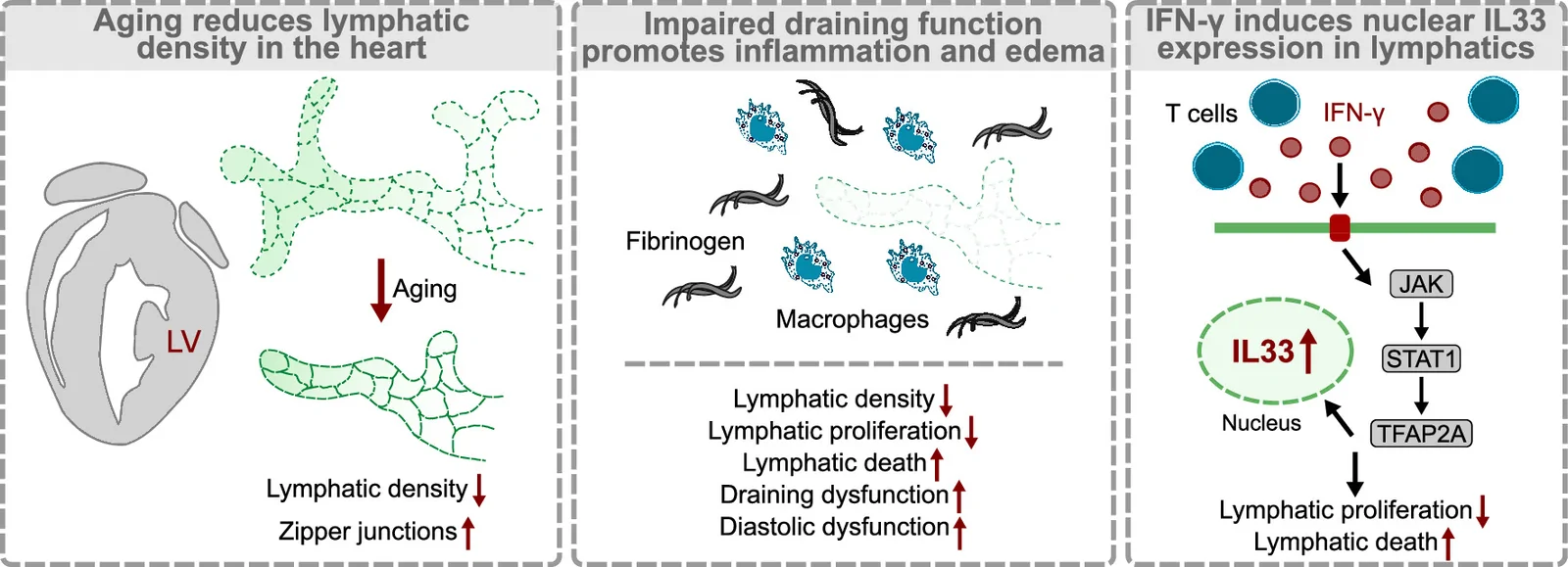
Graphical summary. Advanced age is associated with reduced lymphatic density in the left ventricle and increased abundance of zipper-like junctions. The age-related reduction in lymphatic density is associated with impaired cardiac draining and increased abundance of immune cells, plasma proteins such as fibrinogen and interstitial fluid. Interferon-gamma (IFN-γ) was found to induce nuclear interleukin-33 (IL33) in lymphatic endothelial cells via the JAK-STAT1-TFAP2 pathway. Overexpressing IL33 in lymphatic endothelial cells induced apoptosis and reduced lymphatic endothelial proliferation.

Together, our data suggest that aging leads to a marked reduction in cardiac lymphatic vessel density, accompanied by distinct morphological transformations of the vessels, including the emergence of zipper-like endothelial junctions. These structural changes are paralleled by immune cell infiltration, fibrinogen accumulation, amyloid deposition and myocardial edema—hallmarks of cardiac aging and tissue dysfunction (Extended Data Fig. 9). Experimental depletion of lymphatic vessels via *Flt4* deletion or overexpression of a soluble *Flt4* recapitulates these phenotypes, underscoring a causal relationship. Particularly, we showed that the transfer of immune cells to the lymph nodes was reduced, leading to increased macrophage accumulation in the cardiac tissue. After 2 months of overexpressing soluble *Flt4*, which reduces lymphatic density by about 50%, we observed development of diastolic dysfunction. However, we did not observe changes in fibrosis or cardiac hypertrophy—typical hallmarks of the aging heart. This indicates that a more prolonged period of lymphatic impairment may be required to drive inflammation-dependent cardiac remodeling. Indeed, while reductions in lymphatic vessel density occur as early as 16 months of age in our mouse models, fibrosis and hypertrophy typically appear later, at >18 months.

Notably, our data indicate that lymphatic rarefaction can contribute to diastolic dysfunction through mechanisms independent of fibrosis or hypertrophy. One plausible explanation is increased myocardial edema, which raises interstitial pressure and impairs cardiomyocyte relaxation. Of note, acute ligation of the right and left epicardial lymphatics in dogs has been shown to impair active contraction and relaxation without inducing fibrosis^31^; however, although chronic depletion of lymphatic vessels and lymphatic-derived factors does not appear to affect systolic function^32,33^, its impact on cardiomyocyte relaxation properties has not been explored. Our findings additionally demonstrated increased lipid accumulation in aged hearts, suggesting a potential contribution of lymphatic dysfunction in the promotion of cardiac amyloidosis, a disease with increasing prevalence over the last two decades^34,35^. However, the relevance in the heart is not well studied. Of note, one recent report suggested that although regions with amyloid deposits typically lack lymphatic capillaries, the overall density of lymphatic capillaries was not significantly reduced in this cohort of patients with cardiac amyloidosis^36^. Further studies are needed to elucidate the role of lymphatics in fibrosis and amyloidosis, particularly in the aging heart.

At a molecular level, we identify elevated nIL-33 expression in LymphECs of aged hearts. The full-length IL-33 isoform induced LymphEC death and junctional remodeling, thus mirroring the changes that we observed in aging hearts. IL-33 has complex context and cell-type-specific functions, with not only beneficial but also proinflammatory and profibrotic effects reported in the heart^20–22^. IL-33 protein expression is controlled by splicing, leading to isoforms, which differ in their cellular localization and biological activity. For example, the cytosolic, secreted IL-33 splice variant lacking exons 3 and 4 but not the nuclear form is associated with airway type 2 inflammation^25^. Our findings suggest that, specifically, the full-length, high-molecular-weight nuclear isoform of Il33 impairs lymphatic vessels. The upregulation of this isoform reduced lymphatic cell survival, which could indirectly affect the clearance of the tissue, thereby supporting inflammaging and accumulation of lipids and fluids. This may explain some of the conflicting results regarding the effect of Il33 depletion in mouse models. On the basis of our data, the nuclear Il33 is expected to indirectly enhance inflammation.

To investigate the upstream regulation of IL-33, we exposed HDLECs to a panel of age-associated cytokines. Among those tested, only IFN-γ increased IL-33 expression. Consistent with this, previous studies have shown that IFN-γ induces both the cytokine and nuclear forms of IL-33 in epithelial cells^25,37^. Interferons have also been reported to inhibit lymphangiogenesis and promote apoptosis in LymphECs^38^. These findings align with our observation that IL-33 overexpression triggers HDLEC apoptosis and suppresses proliferation, suggesting that interferons may impair the cardiac lymphatic system through IL-33 induction. The molecular mechanisms underlying IFN-γ-induced IL-33 expression in LymphECs, however, remain unclear. In epithelial cells, IFN-γ activates IL-33 transcription via the p38 mitogen-activated protein kinase (MAPK) and JAK/STAT1 pathways^25^. In line with this, our data demonstrate that JAK/STAT1 and TFAP2A are involved in the control of lymphatics as well (Extended Data Fig. 9). Further defining of the mechanisms responsible for augmenting nuclear Il33 in LymphECs could reveal potential therapeutic targets to counteract age-related lymphatic dysfunction in the heart.

Collectively, our findings position cardiac lymphatic vessels as an early contributor to inflammation and potentially to functional decline in the aging heart. An important unanswered question is whether restoring lymphatic density can prevent or reverse age-associated cardiac pathologies. Recent studies suggest that enhanced lymphangiogenesis contributes to the cardioprotective effects of exercise^13^. Consistently, our data demonstrate that overexpression of *Vegfc* can partially restore lymphatic vessel density and attenuate cardiac inflammaging. Furthermore, selective inhibition of nIL-33 may represent a promising therapeutic strategy for age-related cardiovascular diseases. However, given the chronic and progressive nature of cardiac aging, the potential long-term side effects of any therapeutic intervention must be carefully evaluated. Ultimately, strategies enabling timely, precise and cell-type-specific modulation of lymphatic function may offer the most effective and safest avenues for intervention.

## Methods

### Laboratory animals

Aged male and female C57Bl/6J wild-type mice were purchased from Janvier and from Charles River. Homozygosity of these inbred mice was controlled by Janvier and Charles River using exome sequencing. The mice were kept on a 12-h day–night cycle. All animal experiments were executed in accordance with the guidelines from Directive 2010/63/EU of the European Parliament on the protection of animals used for scientific purposes and were approved by the local authorities by the state of Hesse (Regierungspräsidium Darmstadt, approval nos. FU/2096 and FU/2114) or by the authorities in Helsinki (Eläinkoelautakunta, external approval nos. ESAVI/22042/2019 and ESAVI/30720/2022 and internal approval number KEK22-019).

### Adeno-associated viral vector serotype 9 delivery

To overexpress full-length VEGFC in aged mouse hearts, we administered adeno-associated viral vector serotype 9 (AAV9) vectors carrying the *Vegfc* coding sequence under the control of the CAG promoter. To overexpress soluble VEGFR3 (*sFlt4*) in young mice, we administered AAV9 vectors encoding soluble *Flt4*. AAV9 particles without payload served as a negative control. The viral particles were injected intraperitoneally (i.p.) at a concentration of 2.2 × 10^11^ viral particles per animal.

We silenced *Il33* expression in cardiac lymphatic vessels of aged mice using AAV9 vectors encoding a shRNA targeting *Il33* under the control of the endoglin promoter. Viral particles were coated with a G2-PAMAM linked to an endothelial-targeted peptide (PAMAM-G2^CNN^), which were generated according to Bozoglu et al.^39^ and referred to as EC-AAV9-shIL33. Viral particles were diluted in Opti-MEM GlutaMAXTM (51985026, Gibco) and coated with PAMAM G2CNN (1.8 µg of PAMAM-G2CNN to coat 2 × 10^12^ viral particles) for 30 min at room temperature (RT), before injection. A total of 2 × 10^12^ EC-AAV9-shIL33 were intravenously injected into 18-month-old C57Bl/6J mice. AAV9 encoding for a control shRNA served as a negative control (EC-AAV9-shCtrl). The animal experiment was conducted as approved by the state of Hesse.

### VEGFR3 deletion in LymphECs in vivo

To delete VEGFR3 in LymphECs, the *Vegfr3*-flox line (referred to as *Vegfr3*^*fl/fl*^) was cross-bred with the Prox1-Cre^ERT2^ line (referred to as *Vegfr3*^*iΔLEC*^), as previously described^40^. Tamoxifen was injected i.p. to adult mice on five consecutive days to induce Cre-recombinase expression. Before and 8 weeks after the first injection, heart function was determined using magnet resonance tomography.

### Echocardiography

To assess heart function via echocardiography, mice were anesthetized with 2–2.5% of isoflurane and monitored using the Vevo 3100 echocardiography system with the Vevo LAB software (Fujifilm VisualSonics). Echocardiography was performed before and at 4 and 8 weeks after injection.

### Magnet resonance tomography

Cardiac imaging was performed on a 7.0-T Bruker Pharmascan scanner (Bruker), equipped with a 760 mT m^−1^ gradient system, using a cryogenically cooled four-channel phased-array element 1H receiver-coil (CryoProbe) and a 72-mm RT volume resonator for transmission and the IntraGate self-gating tool. Measurements and analyses were carried out as previously described^41^.

### Edema quantification

To quantify cardiac edemas, the hearts were stained for fibrinogen accumulations (see below) or water content was determined. To quantify cardiac water content, the mice were euthanized, and their hearts were collected without perfusion. Heart weight was determined (wet weight), followed by incubation for 48 h at 65 °C. Heart weight was determined again (dry weight), and the ratio of dry to wet weight was calculated.

### Euthanasia

Mice were anesthetized with 2–3.5% of isoflurane and euthanized via cervical dislocation.

### WB

Protein expression in mouse hearts was analyzed by immunoblotting. In brief, hearts were homogenized in 1 ml of RIPA buffer (89900, Thermo Scientific) containing ¼-inch ceramic spheres two times for 20 s each at 4 m s^−1^. After centrifugation (20,000*g*, 4 °C for 20 min), the supernatants were collected. For immunoblotting, 30 µg of protein lysate was combined with Laemmli buffer, treated for 5 min at 95 °C and separated by SDS–polyacrylamide gel electrophoresis. Proteins that were in gel were then transferred to a nitrocellulose membrane that was blocked using Tris-buffered saline with Tween 20 (TBST) (1061.1, Carl Roth GmbH & Co. KG) containing 5% skimmed milk (sc-281695, Santa Cruz). After blocking, the membranes were incubated in primary antibodies overnight at 4 °C. The membranes were washed three times in TBST for 5 min each and incubated in blocking solution containing the Rb anti-IgG antibody conjugated to horseradish peroxidase (1:5,000; NA934V, Cytiva) for 1 h at RT. After washing three times for 5 min each with TBST, the signal was visualized using the chemiluminescence HRP substrate for enhanced chemiluminescence (WBKLS0500, Merck-Milipore).

The following primary antibodies were used: rabbit anti-GAPDH (1:1,000 for WB; 2118, CST), goat anti-VEGFR3 (1:1,000 for WB; AF743, RnD Systems) and rat anti-IL33 (1:1,000 for WB; MAB5010, RnD Systems).

### Immunofluorescence staining of paraffin sections

Paraffin sections were deparaffinized at 60 °C for 30 min and rehydrated using RotiHistol (two times for 10 min) followed by a descending ethanol series (100%, 95%, 80%, 70% and 50% ethanol; 5 min each). Sections were washed with water for 5 min and incubated in boiling 0.01 M citrate buffer (pH 6.0) for 90–120 s. The slides were blocked with PBS containing 0.1% Triton X-100, 3% BSA (A7030-10G, Merck) and 5% donkey serum (ab7475, Abcam) and incubated with primary antibodies in the blocking solution overnight at 4 °C. Afterward, sections were washed three times in PBS and once in PBS containing 0.1% triton (5 min each) and incubated with the corresponding secondary antibodies and DAPI (6335.1, Carl Roth GmbH & Co. KG), diluted in PBS containing 5% BSA, for 1 h at RT. The sections were washed with PBS two times for 5 min each and mounted with Fluoromount-G (00-4958-02, Invitrogen). Images were taken using the Leica DMi8 Stellaris confocal microscope and LASX software.

### Immunofluorescence of cryopreserved sections

After perfusing mice with PBS, the hearts were taken and fixed overnight at 4 °C in 4% paraformaldehyde (PFA) in PBS (28908, Thermo Fisher Scientific). To assess zipper and button junctions, the hearts were fixed in ice-cold methanol for 4 h. The fixed hearts were washed with PBS three times, 10 min each wash. They were then subjected to three consecutive overnight incubations at 4 °C with increasing concentrations of sucrose (10%, 20% and 30%; S0389, Sigma-Aldrich) in PBS, and the tissues were embedded in 15% sucrose with 8% gelatin (G1890, Sigma-Aldrich), and 1% polyvinylpyrrolidone (P5288, Sigma-Aldrich) in PBS. The embedded tissues were allowed to solidify and were stored at −80 °C. They were then sectioned using the Leica cryostat CM3050 S into 50-µm sections, mounted on adhesive glass slides (10149870, Thermo Fischer Scientific), dried overnight at RT and stored at −20 °C.

For immunofluorescence staining, sections were allowed to thaw at RT for 5 min. They were washed two times with PBS (5 min each) and three times with PBS containing 0.3% Triton X-100 (10 min each) and blocked with 0.1% Triton X-100, 3% BSA (A7030-10G, Merck) and 5% donkey serum (ab7475, Abcam) in PBS for 1 h at RT. After blocking, the sections were incubated with primary antibodies in blocking solution, overnight at 4 °C. The slides were washed with PBS three times (5 min each), and, after one wash with 0.1% Triton X-100 in PBS, incubated with corresponding secondary antibody in PBS containing 5% BSA (A7030-10G, Merck) for 1 h at RT. The slides were then washed two times with PBS for 5 min and mounted with Fluoromount-G (00-4958-02, Invitrogen). Images were captured using the Leica DMi8 confocal Stellaris microscope and LASX software.

### Immunofluorescence staining of cultured cells

The cells were fixed with 4% PFA for 10 min at RT, washed with PBS containing 0.1% Triton X for 10 min and blocked using 2% donkey serum (ab7475, Abcam), 1% BSA (A7030-10G, Merck) and 0.1% Triton X in PBS for 1 h, then incubated with the primary antibodies overnight at 4 °C. The cells were washed with PBS, three times for 5 min, and incubated with the corresponding secondary antibody for 1 h at RT, followed by PBS washes for three times, 5 min each, mounted with Fluoromount-G (00-4958-02, Invitrogen) and imaged with Leica DMi8 confocal Stellaris microscope and the LASX software.

### Whole heart light sheet microscopy

After euthanizing the mice, the hearts were perfused with 4% PFA (28908, Thermo Fisher Scientific) in PBS, isolated and fixed with 3% PFA for 2 h at RT. The PFA was washed with PBS overnight at 4 °C. The hearts were washed with PBS two times for 1 h at RT before incubating in combined blocking and permeabilization solution (1% BSA, 0.1% Triton X-100 and 0.02% sodium azide in PBS) overnight at 4 °C. The samples were then incubated with permeabilization solution containing 2.3% glycine, 20% DMSO, 0.02% sodium azide in PBS–Triton X solution (1% BSA and 0.2% Triton X-100 in PBS) for 2 days at 4 °C and blocking solution containing 10% DMSO, 6% FBS and 0.02% sodium azide in PBS–Triton X solution for again 2 days at 4 °C. The hearts were transferred to primary antibody solution containing the anti-LYVE1 antibody (R&D systems AF2125) in an antibody buffer (5% DMSO and 3% FBS in PTwH solution (0.2% Tween, 0.1% heparin and 0.02% sodium azide in PBS)) for 5 days at 4 °C. They were washed in PTwH solution for 2 days by resubstituting with fresh solution two to three times every day. The hearts were transferred to secondary antibody solution containing donkey anti-goat IgG (H + L) cross-adsorbed secondary antibody, Alexa Fluor 647 (Invitrogen 2175459), for 4 days at 4 °C. PTwH was used again to wash the samples for 2 days and after with PBS overnight. The samples were then subjected to the clearing protocol using ascending concentrations of cold ethanol for 4 h in each concentration, followed by methanol with 5% DMSO for 1 h at 4 °C, methanol with 5% DMSO and 5% hydrogen peroxide for 4 h at 4 °C and finally in 100% ethanol for 1 h at 4 °C. The samples were maintained at RT in 100% ethanol before the transfer to ethyl cinnamate.

### Antibodies used for immunofluorescence

The following primary antibodies were used: rat anti-LYVE1 (RnD Systems, MAB2125), goat anti-PDPN (1:100; RnD Systems, AF3244), rabbit anti-fibrinogen (1:100; Agilent, A008002-2), rabbit anti-COL1A1 (1:100; CST, 72026S), rabbit anti-CD68 (1:100; CST, 97778), rabbit anti-TOMM20 (1:100; Abcam, ab186734), rabbit anti-LC3B (1:400; Abcam, ab192890), rat anti-CD45 (1:100; Millipore, 05-1416), rabbit anti-PH3 (1:100; Sigma-Aldrich, 06-570), rabbit anti-cleaved Casp3 (1:100; CST, 9664), mouse anti-SQSTM1/P62 (1:400; Abcam, ab56416) and mouse anti-α-smooth muscle actin (α-SMA)–Cy3 (1:200; Sigma-Aldrich, C6198-2ML).

The conjugates and secondary antibodies were: streptavidin Alexa Fluor 488 conjugate (1:100; Invitrogen, S32354), donkey anti-rabbit antibody Alexa Fluor 488 (1:200; Invitrogen, A-21206), donkey anti-rabbit secondary antibody Alexa Fluor 555 (1:200; Invitrogen, A-31572) and donkey anti-chicken Alexa Fluor 488 (1:200; Dianova, 703-545-155).

Otherwise, GSL 1–Isolectin B4 (IB4) (1:25; Biozol, VEC-B-1205), wheat germ agglutinin (WGA) and Alexa Fluor 555 conjugate (1:100; W32464, Invitrogen) were used. WGA was diluted together with the secondary antibodies as sera would block WGA binding.

### Assessment of apoptotic cell death by TUNEL assay

The In Situ Cell Death Detection Kit, TMR Red (Roche, 12156792910), was used to detect DNA fragmentation by TUNEL staining. The kit was used according to the manufacturer’s protocol. In brief, lentiviral-transduced HDLECs were seeded in eight-well chamber Ibidi slides (80826, Ibidi; 40,000 cells per well). After 24 h, the cells were fixed (4% PFA in PBS for 10 min at RT), permeabilized and incubated with the TMR‑labeled nucleotide and TdT enzyme mix for 1 h at 37 °C in a humidified atmosphere, resulting in red fluorescence at free 3′‑OH DNA ends, which was visualized by using the Leica Stellaris SP8 confocal microscope.

### Senescence-associated β-galactosidase staining

To quantify cellular senescence in HDLECs, the Senescence β-Galactosidase Staining Kit number 9860 (CST) was used according to the manufacturer’s protocol.

### Analysis of immunofluorescence images

The images were analyzed using Volocity 7 software by Quorum Technologies, and the quantified signals were normalized to the DAPI area.

To visualize button and zipper junctions, CDH5^+^ areas within LYVE1^+^ tubes were displayed as an 8-bit image using Fiji. To quantify button and zipper junctions, a Python-based image analysis pipeline was developed to quantify junctional morphologies in LymphECs using processed immunofluorescence images. The Z-stack images were converted into two-dimensional maximum intensity projections to retain fine junctional structures for the purposes of analysis. Binary masks were generated for each protein channel using empirically determined intensity thresholds, selected by visual inspection of multiple representative images to best separate signal from background. The cadherin signal was isolated through the implementation of a binary mask, which retained only pixels above the upper threshold level and below the lower threshold level. The elimination of blood vessel-associated signals was achieved by masking out the IB4 channel, thus excluding regions that were positive for IB4. The resulting image was then further restricted to lymphatic or mitotic regions by intersecting with either the Lyve1 or PH3 mask, depending on the experimental context.

Contiguous clusters of nonzero pixels were identified using an 8-connectivity approach^42^, where a pixel is considered connected to its horizontal, vertical and diagonal neighbors. Clusters smaller than three pixels were discarded as noise, resulting in the removal of approximately 60–80% of the background signal. The remaining clusters were classified into button (<4.72 μm), intermediate (4.72–7.85 μm) or zipper (>7.85 μm) junctions, using length thresholds adapted from previously published criteria^43^. These measurements enabled the quantification of specific junction types to assess junctional remodeling within lymphatic regions.

### Picrosirius red staining

Heart sections were deparaffinized using wash with RotiHistol, twice for 10 min each, and rehydrated using an ethanol gradient series (100%, 95%, 80%, 70% and 50% ethanol, 5 min each). After 5 min in water, the sections were incubated with 0.1% picrosirius red solution (0.5 g sirius red in 500 ml of saturated aqueous picric acid) for 1 h, then washed twice with acidified water. The stained slides were dehydrated using 100% ethanol, cleared with xylene and mounted using pertex mounting medium (R0080, Histoline).

### Congo red staining

To visualize amyloid depositions, Congo red staining was applied on paraffin sections as described by Wilcock et al.^44^. In brief, paraffin sections were processed as described for picrosirius red staining. Deparaffinized and rehydrated heart sections were incubated with 0.4% Congo red in saturated NaCl solution^44^ (C6767-100g, Sigma-Aldrich) for 1 h at RT. The sections were washed and mounted as described above.

### Flow cytometry analysis

To quantify immune cells in mouse hearts, flow cytometry was used on digested left ventricles. To digest heart tissues, left ventricular tissues were minced and incubated in digestion buffer (RPMI 1640 containing collagenase I (450 U ml^−1^), collagenase XI (125 U ml^−1^), DNAse I (60 U ml^−1^) and hyaluronidase (60 U ml^−1^)) for 30 min at 37 °C. Tissue pieces were transferred together with the digestion buffer to C-tubes (130-096-334, Miltenyi) and were homogenized using the gentle MACS dissociator (130-093-235, Miltenyi) with the preset program ‘mHeart2’. The homogenate was strained over a 40-µm strainer (43-50040-51, pluriSelect) and centrifuged at 500*g* for 10 min. The pellets were resuspended in cell staining buffer (420201, BioLegend) and stained with the Zombie NIR viability dye (423105, BioLegend) and the following antibodies from BioLegend: BV711 CD45 (clone 30-F11), BV605 Ly6C (clone HK1.4), PeCy7 F4/80 (clone BM8), APC CD11b (clone M1/70), APC/Cy7 Ly6G (clone RB6-8C5), PE CD90.2 (clone 30-H12), AF700 CD11c (clone N418) and AF-488 MHC-II (M5 114.15.2).

Cardiac immune cells were defined as: Ly6C^+^ monocytes (CD45^high^, CD11b^high^, Ly6G^low^, F4/80^low^ and Ly6C^high^), macrophages (CD45^high^, CD11b^high^, Ly6G^low^, F4/80^high^ and Ly6C^low^), neutrophils (CD45^high^, CD11b^high^ and Ly6G^high^), T cells (CD45^high^, CD11b^low^ and CD90.2^high^) and dendritic cells (CD45^high^, CD11b^high^, Ly6G^low^, F4/80^low^, Ly6C^low^, CD11c^high^ and MHC-II^high^).

Flow cytometry measurements were obtained using an LSRFortessa X-20 (BD) with the following setup: UV laser (355 nm) with 379/28, 525/50 filters, violet laser (405 nm) with 450/50, 525/50, 610/20, 670/80, 710/50 and 780/60 filters, blue laser (488 nm) with 530/30 and 710/50 filters, yellow/green laser (561 nm) with 586/15, 610/20, 670/30, 710/50 and 780/60 filters, and red (640 nm) laser with 670/30, 730/45 and 780/60 filters. To determine absolute cell numbers, cell suspensions were transferred to BD Trucount tubes (340334, BD Biosciences) before flow cytometric analysis. These tubes contain a predefined number of fluorescent beads, enabling calculation of absolute cell counts per sample and normalization to tissue weight (cells per milligram of tissue). Data analysis was performed with Flowjo version 10.

### RNA isolation

To isolate total RNA from cardiac tissue, the hearts were combined with 700 µl of Qiazol and ¼-inch ceramic spheres and homogenized three times for 20 s at 4 min s^−1^. The miRNeasy Mini kit (217004, Qiagen) was used for further steps of RNA isolation. The RNA concentration was determined by measuring absorption at 260 nm and 280 nm in a NanoDrop ND 2000 spectrophotometer (PeqLab).

### cDNA synthesis and quantitative PCR

The reverse transcription was carried out using reverse transcriptase M-MLV (28025013, Thermo Fisher Scientific) and 500 ng of RNA and assessed using the SYBR Green PCR Master Mix (4385617, Applied Biosystems). The primers were designed and purchased from Merck:

Mouse Vegfc fw (GCTGATGTCTGTCCTGTACCC)

Mouse *Vegfc* rev (AGAAGGTGTTTGTGGCTGCT)

Mouse *Reln* fw (GTCATTCCTGGAGCCACAGATC)

Mouse *Reln* rev (GGACATCATCCAAAGCCCACTG)

Mouse *Apln* fw (AGGCATAGCGTCCTCACCTCTT)

Mouse *Apln* rev (GGTGCAGAAACGACAAAGACGG)

Mouse *Rplp0* fw (GCGTCCTGGCATTGTCTGT)

Mouse *Rplp0* rev (GAAGGCCTTGACCTTTTCAGTAAG)

*Human IL33 fw I* (CAGAGCCTAGATGAGACACCG)

*Human IL33 rev I* (CTGGAAGCCAGGGTCAGAAG)

*Human IL33 fw II* (GAACACAGCAAGCAAAGCCT)

*Human IL33 rev II* (ACATGGGGCAAACTTCTTTGG)

Human *Rplp0* fw (TCGACAATGGCAGCATCTAC)

Human *Rplp0* rev (ATCCGTCTCCACAGACAAGG)

### Assessment of telomere length

To determine telomere length of HDLECs, DNA was extracted using the QIAGEN DNeasy Blood and Tissue Kit (69504, Qiagen). The following primer sequences were used:

Human *TEL1B* fw (GGTTTTTGAGGGTGAGGGTGAGGGTGAGGGTGAGGGT)

Human *TEL1B* rev (TCCCGACTATCCCTATCCCTATCCCTATCCCTATCCCTA)

Human *36B4D* fw (CCCATTCTATCATCAACGGGTAC)

Human *36B4D* rev (CAGCAAGTGGGAAGGTGTAATCC)

### scRNA-seq and data analysis

scRNA libraries were generated using the Gem-X kit (1000686, 10X Genomics), using standard GEM construction, amplification, fragmentation and indexing procedures.

Libraries were sequenced on the Illumina NovaSeq 6000 platform using a S4 flowcell kit (PE100, depth of 50,000 reads/nucleus) with the following specifications: Read 1: 28 base pairs (bp), Read 2: 90 bp, Index 1: 10 bp and Index 2: 10 bp.

Data processing was performed using Scanpy (version 1.10.1)^45^ operated under Python 3.11.8. The FASTQ files were mapped to the mouse reference genome (GRCm38) using the CellRanger (version 7.0.0) software from 10X. The count matrices were processed individually and then combined into one AnnData object. Genes expressed at low levels (<3 cells) and cells with a low number of genes (<800) and high mitochondrial content (>7.5%) were excluded from the analysis. After quality control, gene expression values were normalized and logarithmized using a scaling factor of 10,000. The processed data were integrated with the ECs from a public dataset from young and old mice to increase the statistical power^18,19^. The integration was performed using BBKNN^46^, and the Leiden algorithm was used to cluster the data. Annotation was performed using a curated list of markers^47,48^ and the uniform manifold approximation and projection (UMAP) embeddings were computed. Differential expression analysis was performed using the Wilcoxon rank-sum test and MAST (Model-based Analysis of Single-cell Transcriptomics) with Benjamini–Hochhberh correction for multiple testing.

### Spatial transcriptomics

The processed spatial transcriptomics from the left and the right ventricle were obtained from Kanemaru et al.^14^ and used to estimate the abundance of LymphECs across the different time points for each region (left and right ventricle). The predicted proportions of LymphEC per spots were previously estimated with cell2location as described in the original work^14^.

### Bulk RNA-seq

For genome-wide analysis of gene expression, RNA-seq libraries from isolated mRNA were generated and sequenced by the Institute for Lung Health-Genomics and Bioinformatics, at Justus Liebig University Giessen (Germany). A total amount of 400 ng of RNA per sample was used to enrich for polyadenylated mRNA followed by cDNA sequencing library preparation utilizing the Illumina Stranded mRNA Prep Kit (Illumina) according to the manufacturer’s instructions. After library quality control by capillary electrophoresis (4200 TapeStation, Agilent), cDNA libraries were sequenced on the Illumina NextSeq 2000 platform generating 50-bp paired-end reads.

Mapping was performed utilizing the STAR aligner with the reference genome GRCh38 (version 2024-A) for human gene annotations. The subsequently mapped reads were then used for differential gene regulation analysis on HDLECs with and without IL-33 overexpression.

Differentially expressed genes were calculated in R (version 4.5.1) utilizing the ‘DESeq’ function specified with the Wald test and default parameters implemented in the DESeq2 package^49^. *P* values were adjusted (*P*_adj_) for multiple testing using the procedure of Benjamini and Hochberg and only genes with *P*_adj_ < 0.05 were considered as differentially expressed genes.

Pathway analysis was performed using the webtool EnrichR^50–52^ and the Gene Ontology database. The terms were calculated for positive and negative DEGs separately and sorted according to the Benjamini–Hochberg adjusted *P* value.

### Cell culture

HDLECs were purchased from PromoCell (C-12216) and cultured on fibronectin in PromoCell MV2 medium (C-22221) supplemented with the provided supplement mix (C-39221). The cells were cultured for 72 h at 37 °C and 5% CO_2_ in a humidified atmosphere.

HDLECs were treated with recombinant IFN-γ (30 ng ml^−1^; 285-IF-100, R&D Systems), TNF (10 ng ml^−1^; 300-01 A, Peprotech), IL-1β (100 ng ml^−1^; 201-LB-025, R&D Systems), G-CSF (100 ng ml^−1^; AF-300-23-50UG, Gibco), GM-CSF (50 ng ml^−1^; 300-03-20UG, Gibco), VEGFC (100 ng ml^−1^; 9199-VC-025/CF, R&D Systems), reelin (100 ng ml^−1^; 8546-MR-050, R&D Systems), apelin (100 ng ml^−1^; ab152927, Abcam) and S1P (100 ng ml^−1^; 73914-1MG, Merck) for 24 h or with recombinant VEGFC (100 ng ml^−1^), reelin (100 ng ml^−1^), apelin (100 ng ml^−1^) and S1P (100 ng ml^−1^) for 48 h.

To assess autophagy, HDLECs were cultured in fully supplemented medium or Opti-MEM (51985026, Gibco) to starve the cells. The cells were treated with 10 µM chloroquine for 2 h before fixation.

For JAK inhibition, HDLECs were cultured with 5 µM ruxolitinib (83405S, CST) for 24 h.

### Lentiviral overexpression

Lentiviral particles were generated by transfecting HEK293T cells (CRL-11268, ATCC) with the packing plasmids pCMV∆R8.91 and pMD2.G, as well as the plasmids harboring the IL-33 sequence (VB241001-1197hwt, VectorBuilder) or a stuffer sequence (VB241001-1200pax, VectorBuilder).

IL-33-overexpressing HDLECs were treated with recombinant VEGFC (200 ng ml^−1^), reelin (200 ng ml^−1^) and S1P (200 ng ml^−1^) for 24 h.

### siRNA-mediated gene silencing

To knockdown *IL33* in HDLECs, SMART pool On-Target siRNA pools were used, which were purchased from HORIZON (L-015122-01-0005). To silence STAT1, the SMART pool On-Target siRNA pool L-003543-00-0005 was used. To silence TFAP2A, the SMART pool On-Target siRNA pool L-006348-02-0005 was used. A nontargeting pool served as the negative control (D-001810-10-05). siRNAs (5 nM) were transfected using Lipofectamin RNAiMAX (13778075, Invitrogen), which was diluted in Opti-MEM GlutaMAX (51985034, Gibco).

### Statistical analysis

Data are represented as mean, and error bars indicate standard error of the mean (s.e.m.). The Shapiro–Wilk test was carried out for normality distribution analysis. An unpaired two-sided *t*-test and an ordinary one-way ANOVA with Tukey’s or Dunnett’s post hoc test were used for comparison of two groups and more than two groups, respectively. Not normally distributed data were assessed using a Mann–Whitney test (GraphPad Prism 10.4.2). For scRNA-seq analyses, differential expression analysis was performed using the Wilcoxon rank-sum test and MAST with Benjamini–Hochhberh correction for multiple testing. No statistical method was used to predetermine sample size. The investigators were blinded during experiments and outcome assessment.

### Reporting summary

Further information on research design is available in the Nature Portfolio Reporting Summary linked to this article.

## Supplementary information

Supplementary Information

Reporting Summary

Peer Review File

## Source data

Source Data Fig. 1 Statistical source data.

Source Data Fig. 1 Uncropped images.

Source Data Fig. 2 Statistical source data.

Source Data Fig. 2 Gating strategy and uncropped images.

Source Data Fig. 3 Statistical source data.

Source Data Fig. 3 Uncropped images.

Source Data Fig. 4 Statistical source data.

Source Data Fig. 4 Uncropped images.

Source Data Fig. 5 Statistical source data.

Source Data Fig. 5 Uncropped images.

Source Data Extended Data Fig. 1 Statistical source data.

Source Data Extended Data Fig. 2 Statistical source data.

Source Data Extended Data Fig. 3 Statistical source data.

Source Data Extended Data Fig. 4 Statistical source data.

Source Data Extended Data Fig. 5 Statistical source data.

Source Data Extended Data Fig. 6 Statistical source data.

Source Data Extended Data Fig. 7 Statistical source data.

Source Data Extended Data Fig. 8 Statistical source data.

## Figures and Tables

**Extended Data Table 1:**
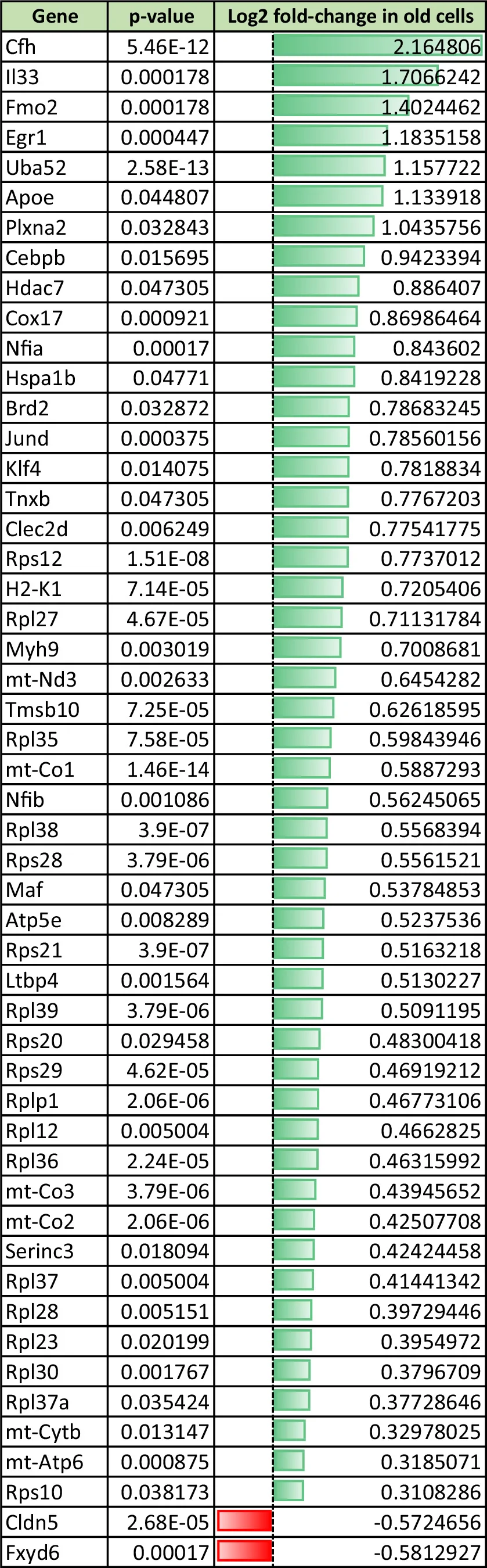
List of differentially expressed genes in the lymphatic endothelial cell cluster of young vs. old mouse hearts

## Data Availability

The RNA-seq datasets that were generated during the present study are available at the ArrayExpress data repository with the accession nos. E-MTAB-17510 and E-MTAB-17485. Supplementary data can be assessed through the online Supplementary Information or upon request.

## Code availability

The full code used during the current study is available via GitHub at https://github.com/davidrm-bio/Wagner_et_al_NatureCardiovascularResearch_2026.
