# Supplementary material for: Age-associated loss of lymphatic vessels promotes cardiac inflammation
Source: Nat Cardiovasc Res. 2026 Sep 15;5(10):961–76. doi: 10.1038/s44161-026-00870-y (PMC13658025; doi:10.1038/s44161-026-00870-y)
Supplement: Supplementary file 3 [file 44161_2026_870_MOESM1_ESM.pdf]

nature cardiovascular research

Supplementary information

https://doi.org/10.1038/s44161-026-00870-y

Age-associated loss of lymphatic vessels promotes cardiac inflammation

In the format provided by the authors and unedited

g

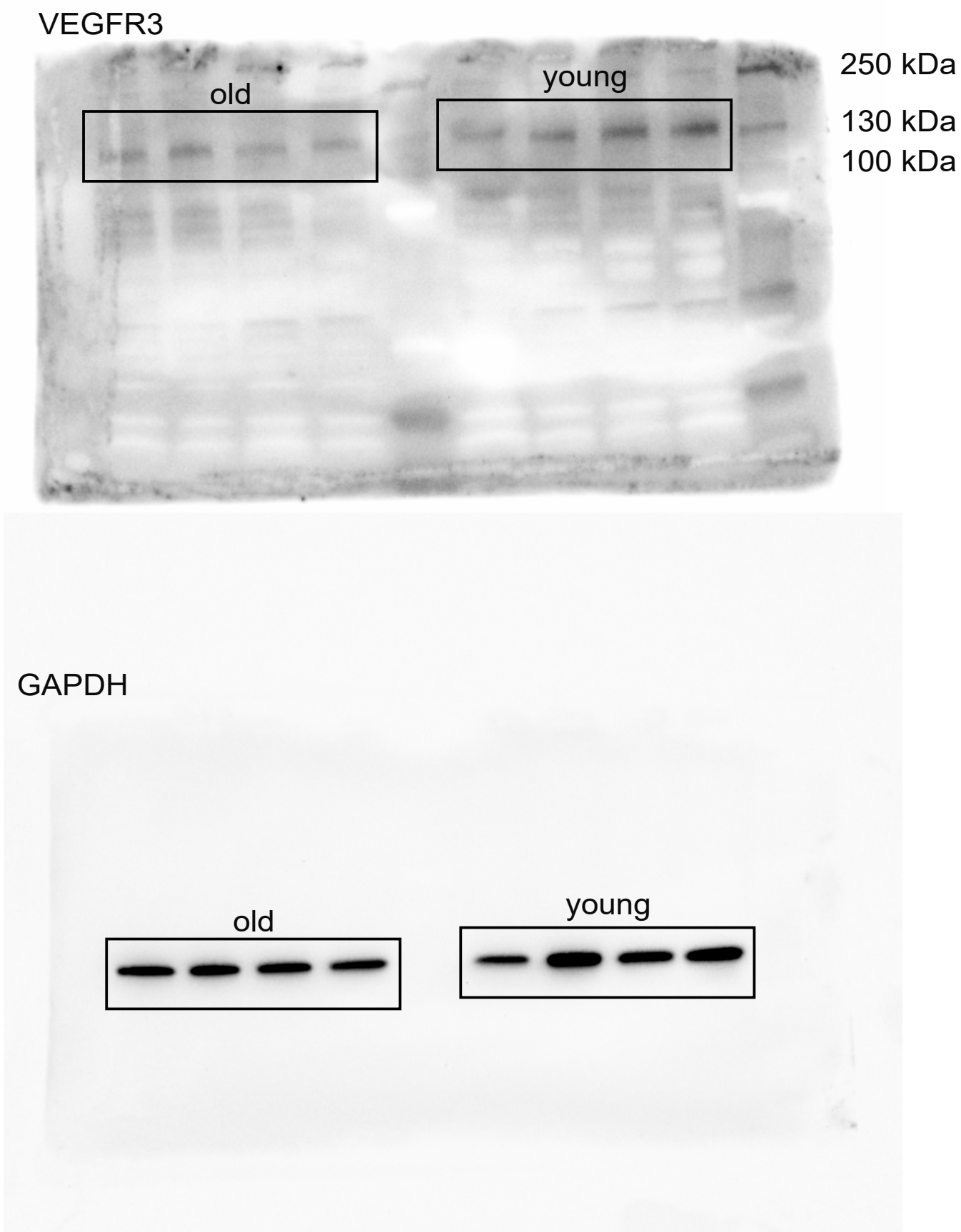

Supplementary Figure 1: Raw uncropped Western Blot to Figure 1g.

c-f

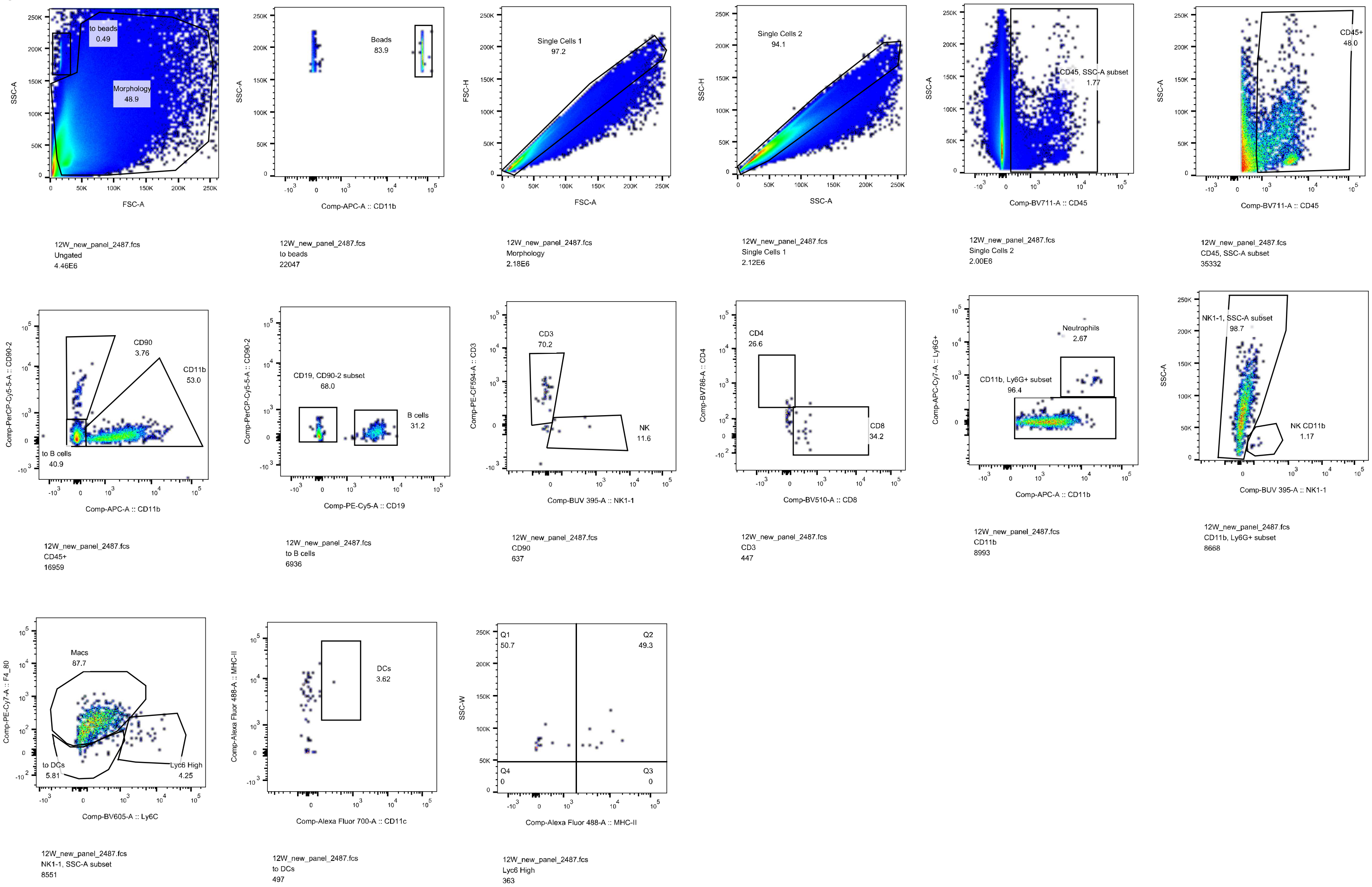

k

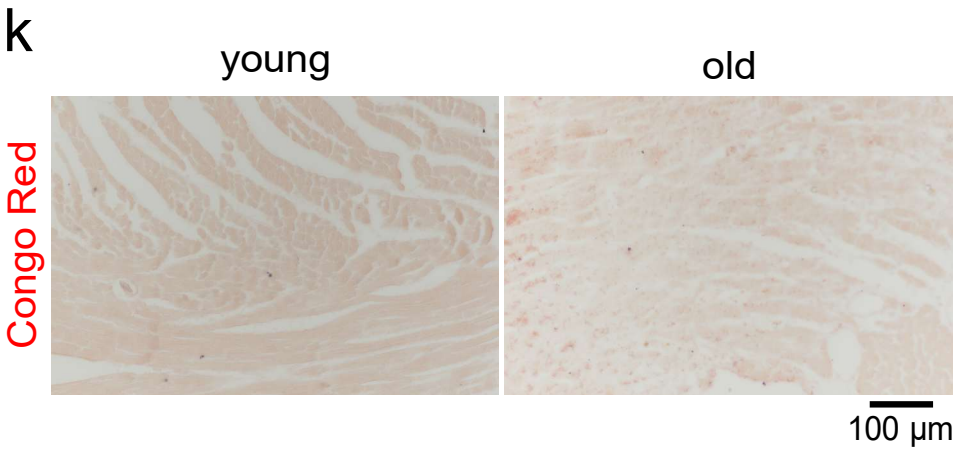

l

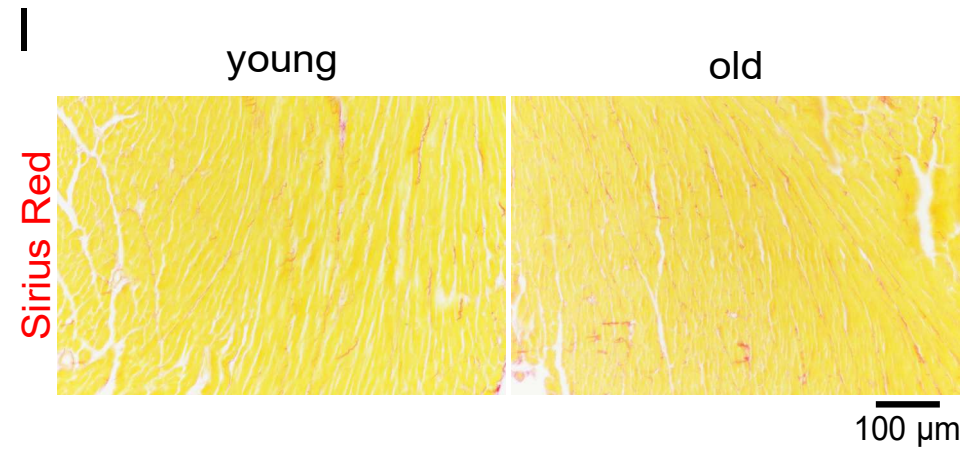

Supplementary Figure 2: Gating strategy to Figure 2c-f and raw uncropped images to Figure 2k, l.

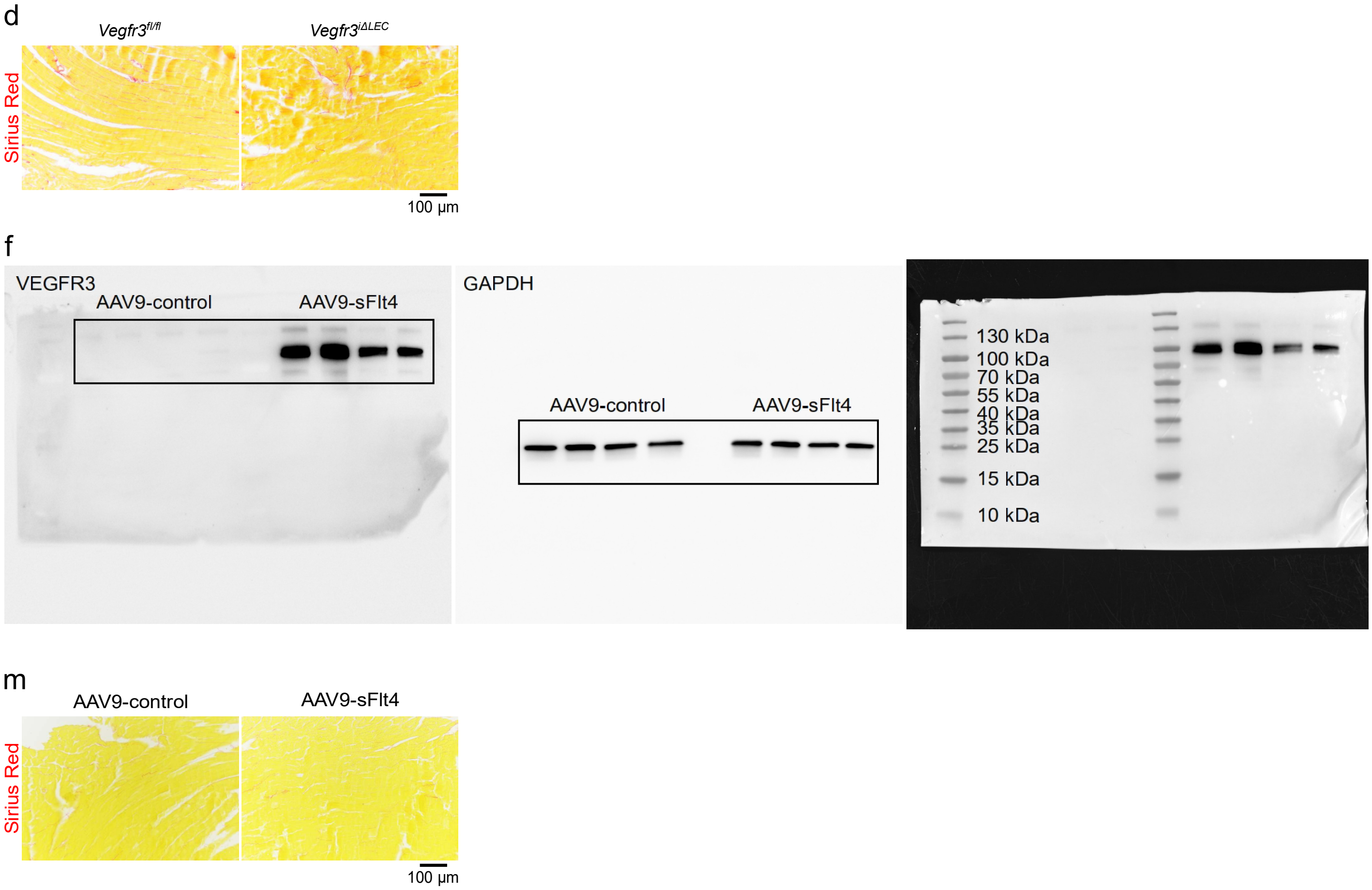

Supplementary Figure 3: Raw uncropped images from Figure 3

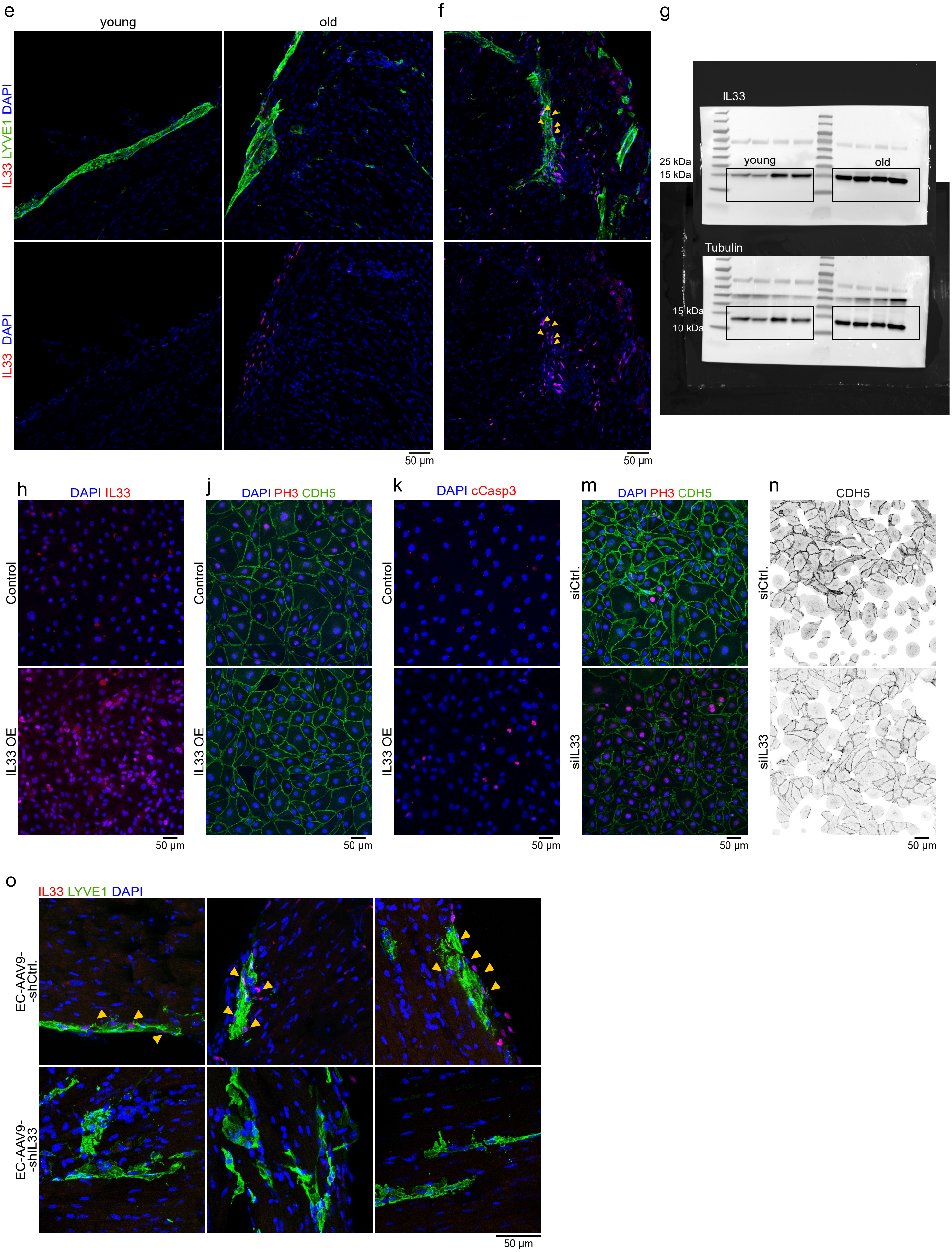

Supplementary Figure 4: Raw uncropped images from Figure 4

h

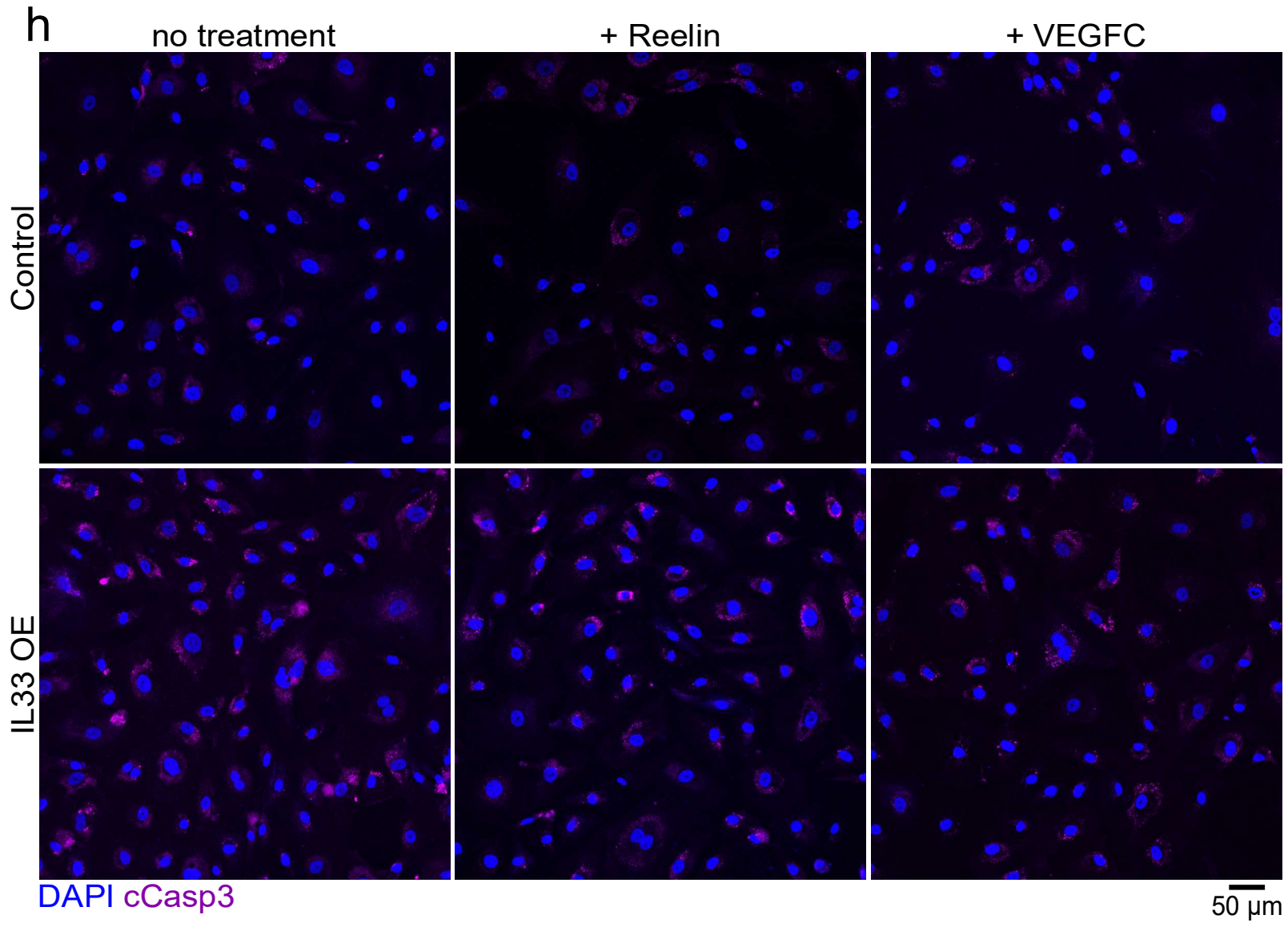

i

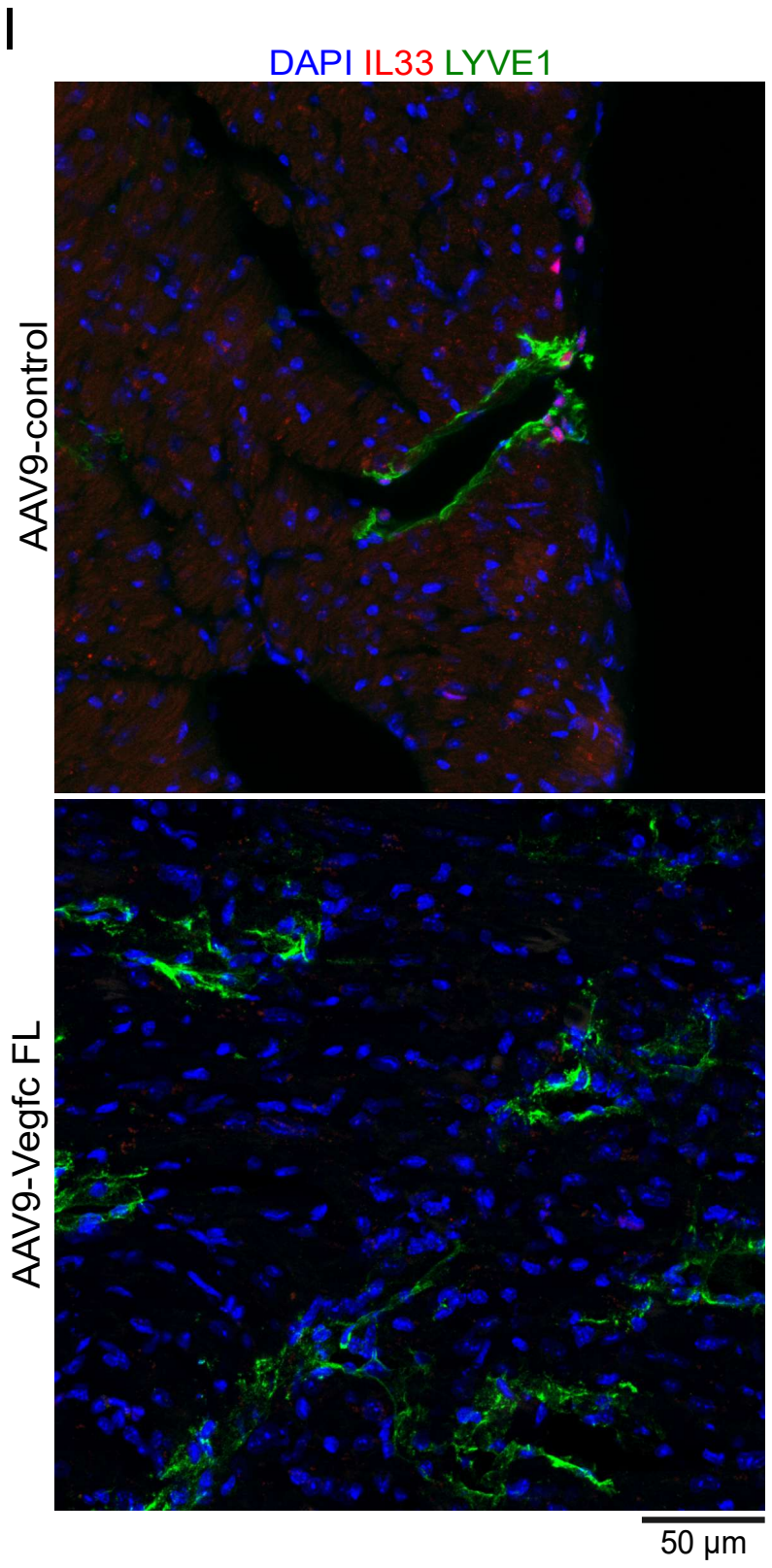

Supplementary Figure 5: Raw uncropped images from Figure 5
