# Supplementary material for: Age-associated loss of lymphatic vessels promotes cardiac inflammation
Source: Nat Cardiovasc Res. 2026 Sep 15;5(10):961–76. doi: 10.1038/s44161-026-00870-y (PMC13658025; doi:10.1038/s44161-026-00870-y)
Supplement: Supplementary file 4 [file 44161_2026_870_MOESM2_ESM.pdf]

nature portfolio

Corresponding author(s): Dr. Julian U.G. Wagner

Last updated by author(s): Aug 10, 2026

Reporting Summary

Nature Portfolio wishes to improve the reproducibility of the work that we publish. This form provides structure for consistency and transparency in reporting. For further information on Nature Portfolio policies, see our Editorial Policies and the Editorial Policy Checklist.

Statistics

For all statistical analyses, confirm that the following items are present in the figure legend, table legend, main text, or Methods section.

n/a	Confirmed	
☐	☒	The exact sample size (*n*) for each experimental group/condition, given as a discrete number and unit of measurement
☐	☒	A statement on whether measurements were taken from distinct samples or whether the same sample was measured repeatedly
☐	☒	The statistical test(s) used AND whether they are one- or two-sided<br>*Only common tests should be described solely by name; describe more complex techniques in the Methods section.*
☐	☒	A description of all covariates tested
☐	☒	A description of any assumptions or corrections, such as tests of normality and adjustment for multiple comparisons
☐	☒	A full description of the statistical parameters including central tendency (e.g. means) or other basic estimates (e.g. regression coefficient) AND variation (e.g. standard deviation) or associated estimates of uncertainty (e.g. confidence intervals)
☐	☒	For null hypothesis testing, the test statistic (e.g. *F*, *t*, *r*) with confidence intervals, effect sizes, degrees of freedom and *P* value noted<br>*Give P values as exact values whenever suitable.*
☒	☐	For Bayesian analysis, information on the choice of priors and Markov chain Monte Carlo settings
☒	☐	For hierarchical and complex designs, identification of the appropriate level for tests and full reporting of outcomes
☒	☐	Estimates of effect sizes (e.g. Cohen's *d*, Pearson's *r*), indicating how they were calculated

Our web collection on statistics for biologists contains articles on many of the points above.

Software and code

Policy information about availability of computer code

Data collection	Open source data were used:<br>https://www.nature.com/articles/s41586-023-06311-1<br>https://www.heartcellatlas.org/<br>https://insight.jci.org/articles/view/131092<br>https://doi.org/10.1161/CIRCRESAHA.125.3270<br>https://www.nature.com/articles/s41467-021-20905-1<br>https://europepmc.org/article/MED/32130914
Data analysis	To analyse scRNA seq data Scanpy and R-studios was used.<br>For statistical assessment of the wet lab data GraphPad Prism 10.4.2 was used.<br>To analyse microscopy images Volocity 7 by Quorum and ImageJ / FIJI were used.<br>Inkscape was used to illustrate schematic images and the graphical summary.

For manuscripts utilizing custom algorithms or software that are central to the research but not yet described in published literature, software must be made available to editors and reviewers. We strongly encourage code deposition in a community repository (e.g. GitHub). See the Nature Portfolio guidelines for submitting code & software for further information.

Data

Policy information about availability of data

All manuscripts must include a data availability statement. This statement should provide the following information, where applicable:

- Accession codes, unique identifiers, or web links for publicly available datasets
- A description of any restrictions on data availability
- For clinical datasets or third party data, please ensure that the statement adheres to our policy

The RNA sequencing data sets that were generated during the present study are available at the ArrayExpress data repository with the accession number E-MTAB-17510 and E-MTAB-17485.

Research involving human participants, their data, or biological material

Policy information about studies with human participants or human data. See also policy information about sex, gender (identity/presentation), and sexual orientation and race, ethnicity and racism.

Reporting on sex and gender	To assess the cardiac lymphatic system in mice, we used both sexes: males and females.<br>To manipulate lymphatics in aged mice, we used male mice only because availability of this sex. For genetic manipulation, female mice were used, too.<br><br>The respective sex has been stated in the figure legends.
Reporting on race, ethnicity, or other socially relevant groupings	Does not apply.
Population characteristics	Does not apply.
Recruitment	Does not apply.
Ethics oversight	Does not apply.

Note that full information on the approval of the study protocol must also be provided in the manuscript.

Field-specific reporting

Please select the one below that is the best fit for your research. If you are not sure, read the appropriate sections before making your selection.

☒ Life sciences ☐ Behavioural & social sciences ☐ Ecological, evolutionary & environmental sciences

For a reference copy of the document with all sections, see nature.com/documents/nr-reporting-summary-flat.pdf

Life sciences study design

All studies must disclose on these points even when the disclosure is negative.

Sample size	No sample size was calculated. Sample size was chosen according to previous experiences.
Data exclusions	Data points were excluded only when identified as significant outliers, as determined using GraphPad Prism version 10.4.2.
Replication	Multiple independent experiments were used such as lymphatic manipulation using AAV9 or genetic deletion of Flt4 were used to ensure reproducibility.
Randomization	Mice were randomly chosen for AAV9 injections.
Blinding	Data / images / echocardiography data were analysed by blinded technicians.

Reporting for specific materials, systems and methods

We require information from authors about some types of materials, experimental systems and methods used in many studies. Here, indicate whether each material, system or method listed is relevant to your study. If you are not sure if a list item applies to your research, read the appropriate section before selecting a response.

Materials & experimental systems

n/a	Involved in the study	
☐	☒	Antibodies
☐	☒	Eukaryotic cell lines
☒	☐	Palaeontology and archaeology
☐	☒	Animals and other organisms
☒	☐	Clinical data
☒	☐	Dual use research of concern
☒	☐	Plants

Methods

n/a	Involved in the study	
☒	☐	ChIP-seq
☐	☒	Flow cytometry
☒	☐	MRI-based neuroimaging

Antibodies

Antibodies used

The following primary antibodies were used: Rat anti-LYVE1 (RnD Systems, MAB2125); Goat anti-PDPN (1:100, RnD Systems, AF3244); Rabbit anti-fibrinogen (1:100, Agilent, A008002-2); Rabbit anti-COL1A1 (1:100, CST, 72026S); Rabbit anti-CD68 (1:100, CST, 97778), Rabbit anti-TOMM20 (1:100, Abcam, ab186734), Rabbit anti-LC3B (1:400, Abcam, ab192890), Rat anti-CD45 (1:100, Millipore, 05-1416), Rabbit anti-PH3 (1:100, Sigma-Aldrich, 06-570), Rabbit anti-cleaved Casp3 (1:100, CST, 9664), Mouse anti-SQSTM1/P62 (1:400, Abcam, ab56416), and Mouse anti-α-smooth muscle actin - Cy3™ (αSMA, 1:200, Sigma-Aldrich, C6198-2ML).
For flowcytometry, antibodies were purchased from BioLegend: BV711 CD45 (clone 30-F11), BV605 Ly6C (clone HK1.4), PeCy7 F4/80 (clone BM8), APC CD11b (clone M1/70), APC/Cy7 Ly6G (clone RB6-8C5), PE CD90.2 (clone 30-H12), AF700 CD11c (clone N418), AF-488 MHC-II (M5 114.15.2)

Validation

All antibodies have been validated by the respective manufacturer:
Rat anti-LYVE1 (RnD Systems, MAB2125 - https://www.rndsystems.com/products/mouse-lyve-1-antibody-223322_mab2125
Goat anti-PDPN (1:100, RnD Systems, AF3244) - https://www.rndsystems.com/products/mouse-podoplanin-antibody_af3244
Rabbit anti-fibrinogen (1:100, Agilent, A008002-2) - https://www.agilent.com/store/de_DE/Prod-A008002-2/A008002-2?srsltid=AfmBOor8ygZF6e8pv4J4CvXe2qsjrxU8q6laKXPVBGG-VffaWzuS5gwE
Rabbit anti-COL1A1 (1:100, CST, 72026S) - https://www.cellsignal.com/products/primary-antibodies/col1a1-e8f4l-rabbit-monoclonal-antibody/72026
Rabbit anti-CD68 (1:100, CST, 97778) - https://www.cellsignal.com/products/primary-antibodies/cd68-e3o7v-rabbit-monoclonal-antibody/97778
Rabbit anti-TOMM20 (1:100, Abcam, ab186734) - https://www.abcam.com/en-us/products/primary-antibodies/tomm20-antibody-epr15581-39-mitochondrial-marker-ab186734
Rabbit anti-LC3B (1:400, Abcam, ab192890) - https://www.abcam.com/en-us/products/primary-antibodies/lc3b-antibody-epr18709-autophagosome-marker-ab192890
Rat anti-CD45 (1:100, Millipore, 05-1416) - https://www.merckmillipore.com/DE/de/product/Anti-CD45-Antibody-clone-IBL-5-25,MM_NF-05-1416
Rabbit anti-PH3 (1:100, Sigma-Aldrich, 06-570) - https://www.sigmaaldrich.com/DE/de/product/mm/06570?srsltid=AfmBOorKsSnF3422BNUpO-UeM5x7r8jGcl87ujfeReYc8D0pbZqGj_gt
Rabbit anti-cleaved Casp3 (1:100, CST, 9664) - https://www.cellsignal.com/products/primary-antibodies/cleaved-caspase-3-asp175-5a1e-rabbit-monoclonal-antibody/9664
Mouse anti-SQSTM1/P62 (1:400, Abcam, ab56416) - https://www.abcam.com/en-us/products/primary-antibodies/sqstm1-p62-antibody-2c11-bsa-and-azide-free-ab56416
Mouse anti-α-smooth muscle actin - Cy3™ (αSMA, 1:200, Sigma-Aldrich, C6198-2ML) - https://www.sigmaaldrich.com/DE/de/product/sigma/c6198
BV711 CD45 (clone 30-F11) - https://www.biolegend.com/de-de/products/brilliant-violet-711-anti-mouse-cd45-antibody-10439
BV605 Ly6C (clone HK1.4) - https://www.biolegend.com/de-de/products/brilliant-violet-605-anti-mouse-ly-6c-antibody-8727
PeCy7 F4/80 (clone BM8) - https://www.biolegend.com/en-gb/products/pe-cyanine7-anti-mouse-f4-80-antibody-4070?GroupID=BLG5319
APC CD11b (clone M1/70) - https://www.biolegend.com/de-de/products/apc-anti-mouse-human-cd11b-antibody-345
APC/Cy7 Ly6G (clone RB6-8C5) - https://www.biolegend.com/de-de/products/apc-cyanine7-anti-mouse-ly-6gly-6c-gr-1-antibody-3935
PE CD90.2 (clone 30-H12) - https://www.biolegend.com/de-de/products/pe-anti-mouse-cd90-2-thy1-2-antibody-106
AF700 CD11c (clone N418) - https://www.biolegend.com/de-de/products/alexa-fluor-700-anti-mouse-cd11c-antibody-3429
AF-488 MHC-II (M5 114.15.2) - https://www.biolegend.com/de-de/products/alexa-fluor-488-anti-mouse-i-a-i-e-antibody-3134

Eukaryotic cell lines

Policy information about cell lines and Sex and Gender in Research

Cell line source(s)

Human Dermal Lymphatic endothelial cells were purchased from PromoCell (C-12216).
HEK293T cells (CRL-11268) were purchased from ATCC.

Authentication

HDLECs were freshly purchased from the manufacturer and not longer used as passage 7. Staining for PROX1 was used to verify lymphatic lineage.
HEK293T cells were not authenticated by the authors.

Mycoplasma contamination

Tested negatively by the manufacturer.

Commonly misidentified lines
(See ICLAC register)

Does not apply.

Animals and other research organisms

Policy information about studies involving animals; ARRIVE guidelines recommended for reporting animal research, and Sex and Gender in Research

Laboratory animals	Wildtype C57Bl\6J (Janvier, France), Vegfr3;Prox1-CreERT2 (Lab of Kari Alitalo, Finland)
Wild animals	Wild animals were not used in this study.
Reporting on sex	The respective sex has been stated in the respective figure legend. Both sexes were used.
Field-collected samples	No field-collected samples were used.
Ethics oversight	All animal experiments have been executed in accordance to the guidelines from Directive 2010/63/EU of the European Parliament on the protection of animals used for scientific purposes and were approved by the local authorities by the state of Hesse (Regierungspräsidium Darmstadt, approval numbers FU/2096 and FU/2114) or by the authorities in Helsinki.

Note that full information on the approval of the study protocol must also be provided in the manuscript.

Plants

Seed stocks	N.A.
Novel plant genotypes	N.A.
Authentication	N.A.

Flow Cytometry

Plots

Confirm that:

☒ The axis labels state the marker and fluorochrome used (e.g. CD4-FITC).

☒ The axis scales are clearly visible. Include numbers along axes only for bottom left plot of group (a 'group' is an analysis of identical markers).

☒ All plots are contour plots with outliers or pseudocolor plots.

☒ A numerical value for number of cells or percentage (with statistics) is provided.

Methodology

Sample preparation	To quantify immune cells in mouse hearts, flow cytometry was used on digested left ventricles. To digest heart tissues, left ventricular tissues were minced and incubated in digestion buffer (RPMI 1640 containing collagenase I (450 U/mL), collagenase XI (125 U/mL), DNAse I (60 U/mL) and hyaluronidase (60 U/mL)) for 30 minutes at 37°C. Tissue pieces were transferred together with the digestion buffer to C-tubes (130-096-334, Miltenyi) and were homogenized using the gentle MACS dissociator (130-093-235, Miltenyi) with the pre-set program “mHeart2”. The homogenate was strained over a 40 µm strainer (43-50040-51, pluriSelect) and centrifuged at 500 g for 10 minutes.
Instrument	Flow cytometry measurements were obtained using an LSRFortessaTM X-20 (BD) with the following setup: UV laser (355 nm) with 379/28, 525 /50 filters; Violet laser (405nm) with 450/50, 525/50, 610/20, 670/80, 710/50, 780/60 filters; Blue laser (488 nm) with 530/30, 710/50 filters; Yellow/green laser (561 nm) with 586/15, 610/20, 670/30, 710/50, 780/60 filters; Red (640 nm) laser with 670/30, 730/45, 780/60 filters.
Software	Flowjo v10
Cell population abundance	CD45+ cells are highly abundant cells in the murine heart. CD45+ subpopulations were quantified and compared between young and aged mice. Cells were not sorted for subsequent culture.
Gating strategy	Cardiac immune cells were defined as: Ly6C+ monocytes (CD45high, CD11bhigh, Ly6Glow, F4/80low, Ly6Chigh), macrophages (CD45high, CD11bhigh, Ly6Glow, F4/80high, Ly6Clow), neutrophils (CD45high, CD11bhigh, Ly6Ghigh), T-cells

(CD45high, CD11blow, CD90.2high), dendritic cells (CD45high, CD11bhigh, Ly6Glow, F4/80low, Ly6Clow, CD11chigh, MHC-IIhigh).

☒ Tick this box to confirm that a figure exemplifying the gating strategy is provided in the Supplementary Information.
