# Supplementary material for: Age-associated loss of lymphatic vessels promotes cardiac inflammation
Source: Nat Cardiovasc Res. 2026 Sep 15;5(10):961–76. doi: 10.1038/s44161-026-00870-y (PMC13658025; doi:10.1038/s44161-026-00870-y)
Supplement: Supplementary file 5 [file 44161_2026_870_MOESM3_ESM.pdf]

nature portfolio

# Peer Review File

# Age-Associated Loss of Lymphatic Vessels Promotes Cardiac Inflammation

Corresponding Author: Dr Julian Wagner

**This file contains all reviewer reports in order by version, followed by all author rebuttals in order by version.**

Version 0:

Reviewer comments:

Reviewer #1

(Remarks to the Author)
This study demonstrates that a loss of lymphatic vessels can be observed during aging in mice, associated also with changes in lymphatic vessel architecture. These findings correlated with an increase in immune cells, including T cells and macrophages in the heart, an increase in water retention, fibrinogen accumulation, and fibrosis. Deletion of VEGFR3 in LECS in young mice reduced lymphatic vessel counts, and was again associated with an increase in macrophages and fibrinogen, and similar findings were observed upon overexpression of sFLT4, confirming that VEGFR3 plays a critical role in lymphatic vessel formation and in the heart has a role in clearing inflammation and edema. The authors then identify upregulation of IL-33 in aged LECs, and in vitro show that IL-33 regulates LEC proliferation and apoptosis, and can be induced by IFNg, expressed by T cells in the heart. VEGFC treatment was shown to reduce IL-33 expression. Finally, the AAV-induced VEGFC overexpression increased lymphatic vessel counts and reduced macrophage accumulation in aged mice.
The loss of lymphatic vessels, which may contribute to age-associated deterioration in cardiac function, and findings related to IL-33, are novel and interesting.

In sections through the heart (Fig. 1b-d, 2a), LYVE1 staining may have also comprised Lyve1+ macrophages. Co-staining of macrophages should be performed to exclude macrophage “contamination” of lymphatic analyses. It is not entirely clear how sections of the sub-endocardial area were generated; also, the exact location of sections in the heart in Figure 1c is unclear. Zipper and Button-junctions were counted per image; it may be more meaningful to assess these relative to the lymphatic vessel area.

In general, it has previously been shown that only LYVE-1 + precollector-like vessels (> 20 μm in diameter) express podoplanin. Lymphatic podoplanin expression is less frequent in midmyocardial areas, and the vast majority of LYVE-1+ lymphatic capillaries (>80%) are small (diameter <5 μm) and negative for podoplanin. The location of the lymphatics measured in this study and their co-expression with PDPN require clarification, in particular, as sometimes vessels are only quantified using LYVE1 or PDPN
Also, the topography of the other changes described needs to be described in more detail. Eg. for quantification of macrophages, which region of the heart has been analyzed.

Macrophages are represented relative to the Dapi+ area, whereas T cells and DC were quantified per mg LV tissue. Please clarify.

Representative images of Fibrinogen should be shown (Fig. 2i). Cardiac hypertrophy is mentioned in the text but not shown. It would be informative to also include evaluations of the heart function of mice during aging and in Vegfr3LEC mice.

The increase in diastolic function in hearts after sFlt4 overexpression is interesting; what could be the underlying reason for this finding?

Data on cardiomyocyte size in sFlt4 overexpression is duplicated (Fig. 3m, and Supp. Fig. 3).

It should be demonstrated that the nuclear isoform of IL33 protein is induced in lymphatics in vitro upon OE and siIL33 treatment. Are levels of IL-33 altered in mice treated with AAV9-Vegfc FL?

Effects of IL-33 have been studied in vitro using human dermal lymphatic endothelial cells; whether lymphatic vessel rarefaction in vivo is connected to IL-33 remains unclear. This is of particular relevance, as VEGF-C levels seem to decrease in aging hearts, and overexpression of this cytokine increases lymphatic vessel counts also in old mice, a response expected from previous studies, but possibly unrelated to IL-33.

Is the decline in macrophages in the mediastinal lymph node specific, or are similar shifts observed in other lymph nodes, as well?

The number of mice is mostly very low.

Reviewer #2

(Remarks to the Author)

Wagner et al. have addressed an important and clinically-relevant question of whether and how aging influences cardiac lymphatic rarefaction and immune cells interactions. Using cohorts of young and old mice, the authors show reduced lymphatic density with aging which is accompanied by increased tissue immune cell infiltration and myocardial edema. The most novel finding of the study is a selective upregulation of nuclear IL33 which they show using in vitro cell culture studies, stimulates lymphatic endothelial cell death and junctional remodeling. As expected, VegfC overexpression in animal models drives cardiac lymphatic growth, thereby restoring the aging effects on cardiac lymphatics, but the ultimate effects on nIL33 or cardiac function are not investigated. Overall, this is a timely study with compelling preliminary findings and a plausible mechanistic linking of cardiac lymphatic rarefaction with inflammation and aging. Addressing the following comments would improve the impact of the study.
1. An overall comment and area for improvement is related to poor image quality. Many of the images rely on visualizing a dim green signal on a black background. Even with optical zoom, the staining is barely visible.
2. The finding of increased lymphatic diameter, Fig 1e, is difficult to interpret and based on the limited data presented an over-interpretation. In the representative “old” image, what do the authors define as the lumen and wall of the labeled structures? Do the data points in the graph represent a single vessel measurement, in 3X different animals? It appears that there are only 2X data points for the “young” analysis, which is too low to make rigorous conclusions. In addition, the finding of 15% increased diameters in old hearts is not evident across other images and data.
3. The data in Figure 1h-k on button versus zipper junctions is unconvincing. The representative image of a young lymphatic, which is presumably one of the better selected images, is uninterpretable.
4. Fig 1.c,d shows sex differences in aging phenotypes, with males losing more Lyve1+ cells. Unless I missed it, the sex of the animals for analyses in the remainder of the study are not defined. This would be particularly relevant for analyses and methods that rely on Lyve1 positivity, like Figs. 2 and 3. Were sexes combined?
5. The Congo red data, Fig. 2j,k are not convincing. The staining image does not appear to reflect the quantified data. Please provide representative images and images for Sirius red.
6. A confounding and technical limitation of the study is the isolation and analysis of Cdh5+ endothelial cells to perform gene expression analysis (Fig. 4), from which much of the mechanistic data is derived. The authors computationally enriched for lymphatic clusters based on prior datasets, but cardiac LECs should be isolated separately to provide more rigorous gene expression analysis of this population of cells. As currently written, the authors cannot claim these data or identified genes are derived from lymphatic endothelial cells.
7. The mechanistic significance of nIL33 in aged cardiac lymphatics is suggested but not validated in vivo. In this context, the authors should use a lymphatic endothelial cell–specific IL–33 knockout model to directly demonstrate the role of IL 33 in aged cardiac lymphatics.
7a. The authors should include some assessments of recognized cellular aging mechanisms, such as telomere shortening, genomic instability, mitochondrial dysfunction, oxidative damage, autophagy, or epigenetic alterations, to strengthen the rigor of their conclusions.
8. Please provide images to support conclusion of Fig. 4n.
9. Lines 217-220: The authors should discuss why the lymphatic-secreted factor reelin was not decreased in aged hearts when the main finding of the study is reduced cardiac lymphatic density.
10. In vivo studies in Fig. 5k-n are underpowered.
11. What were the in vivo levels of IL33 and nIL33 in the VegfC overexpression model?
12. Line 206 refers to AAV9-Vegfc treatment effects on cardiac diastolic function. Where is the cardiac functional data?
13. A graphical abstract or mechanism figure may be helpful for readers.
14. Line 255-257: “….however, the impact of a chronic reduction of lymphatics and lymphatic cell-derived factors on cardiomyocyte contraction and relaxation properties has not been explored.” See PMID: 34789016, PMID: 34403369

Reviewer #3

(Remarks to the Author)
This study by Wagner et al. investigated age-associated changes in cardiac lymphatic vessels. The authors report that cardiac lymphatics are reduced with aging, which is associated with increased inflammation and fibrosis. Using lymphatic-specific Vegfr3 knockout mice or AAV9-mediated overexpression of soluble Flt4 in young mice, they show that reduction of cardiac lymphatics leads to cardiac inflammation and dysfunction, but not fibrosis. They further identify nuclear IL-33 as a potential mechanism underlying age-related loss of cardiac lymphatics and cardiac dysfunction. Specifically, nuclear IL-33 is upregulated in lymphatic endothelial cells in aged hearts. Its expression is increased by IFN-γ but reduced by VEGF-C. The use of in vivo genetic and pharmacological interventions combined with single-cell RNA sequencing and spatial

transcriptomics is impressive. Overall, the findings support the concept that enhanced lymphangiogenesis is cardioprotective, particularly in the aged heart. However, several concerns need to be addressed:

1. Experimental design of the Vegfr3 deletion model: The experimental design of the lymphatic-specific Vegfr3 deletion model is not clearly described. Were the mice injected with tamoxifen at day of birth (P0)? The Methods section states that mice were "injected at day 0 and 1… and cardiac function was determined before and 4 and 8 weeks after the first injection," which is confusing. In addition, cardiac functional data from these mice are not shown. Does Vegfr3 deletion also lead to diastolic dysfunction, similar to the AAV9-sFlt4 treatment? In addition, were there any changes in body weight following Vegfr3 deletion or AAV-sFlt4 treatment?

2. Identification of nuclear IL-33 as a key mechanism is intriguing. However, the expression of nuclear IL-33 is primarily based on single-cell RNA sequencing of isolated endothelial cells. Although IL-33 is among the top differentially expressed genes in lymphatic endothelial cells, nuclear IL-33 is also known to be expressed in other cardiac cell types, such as fibroblasts. Does nuclear IL-33 expression also change in other cardiac cell populations during aging?

3. Functional studies of nuclear IL-33 are largely performed in vitro. Given that nuclear IL-33 increases beginning at ~16 months of age, does inhibition of nuclear IL-33 in vivo affect cardiac inflammation and function in aged mice? While the in vitro data clearly show that nuclear IL-33 overexpression inhibits lymphangiogenesis, are there insights into the molecular mechanisms by which nuclear IL-33a exerts this effect?

4. The data indicate that IFN-γ induces, whereas VEGF-C suppresses, nuclear IL-33 expression. Are there mechanistic insights into how these signaling pathways regulate nuclear IL-33 expression?

5. It is somewhat surprising that overexpression of full-length VEGF-C did not alter blood capillary density, given that the mutant form VEGF-C C156S is often used to avoid angiogenic effects on blood vessels. Could the authors comment on this observation?

6. The in vitro data show that VEGF-C downregulates nuclear IL-33. In the in vivo VEGF-C overexpression studies, does VEGF-C improve cardiac function in aged mice? Furthermore, is the enhancement of lymphangiogenesis by VEGF-C associated with a reduction in nuclear IL-33 expression in vivo?

Version 1:

Reviewer comments:

Reviewer #1

(Remarks to the Author)
The authors have addressed my previous concerns.

Reviewer #2

(Remarks to the Author)
The authors have completed an extensive series of experiments to address most of the suggestions of reviewers. The new data to support a mechanistic pathway for nuclear IL-33 regulation is very valuable, as are expanded references. The in vivo silencing of IL33 to rescue lymphatic density in aged hearts is compelling, convincing and a key data element in support of the overall findings.

Reviewer #3

(Remarks to the Author)
The authors have addressed the comments by providing substantial new data.

Some data were generated solely to address the reviewers' comments and were not incorporated into the revised manuscript. Including some of these key findings in the final version would be informative. For example, the data shown in Figure for Reviewers 1.1 (LYVE1 and CD68 staining) and Figure for Reviewers 1.2 (the strategy for quantifying lymphatic capillaries) would be informative additions.

**Open Access** This Peer Review File is licensed under a Creative Commons Attribution 4.0 International License, which permits use, sharing, adaptation, distribution and reproduction in any medium or format, as long as you give appropriate credit to the original author(s) and the source, provide a link to the Creative Commons license, and indicate if changes were made.
In cases where reviewers are anonymous, credit should be given to 'Anonymous Referee' and the source.
The images or other third party material in this Peer Review File are included in the article's Creative Commons license, unless indicated otherwise in a credit line to the material. If material is not included in the article's Creative Commons license and your intended use is not permitted by statutory regulation or exceeds the permitted use, you will need to obtain permission directly from the copyright holder.
To view a copy of this license, visit https://creativecommons.org/licenses/by/4.0/

## First round of revision (Comments from January 19th 2026)

### Response to editors' comments:

1. Please provide in vivo functional evidence demonstrating the role of IL-33 in aged cardiac lymphatics, including the impact of IL-33 inhibition on cardiac inflammation and cardiac function in aged mice.

**Answer:** To support the hypothesis that IL33-mediates age-related lymphatic regression, we now demonstrate that VEGFC FL overexpression in aged mice reduce the IL33 expression in cardiac lymphatics (**main Figure 5l of the revised manuscript**). We further generated an endothelial-targeted AAV9 system to silence IL33 expression by overexpressed shRNAs under the endoglin promotor targeting IL33. We demonstrate that IL33 expression is reduced in cardiac lymphatics of aged mice (**Figure 1a for the editors**). Repressing IL33 resulted in elevated lymphatic capillary density and reduces macrophage abundance (**Figure 1b, c for the editors**).

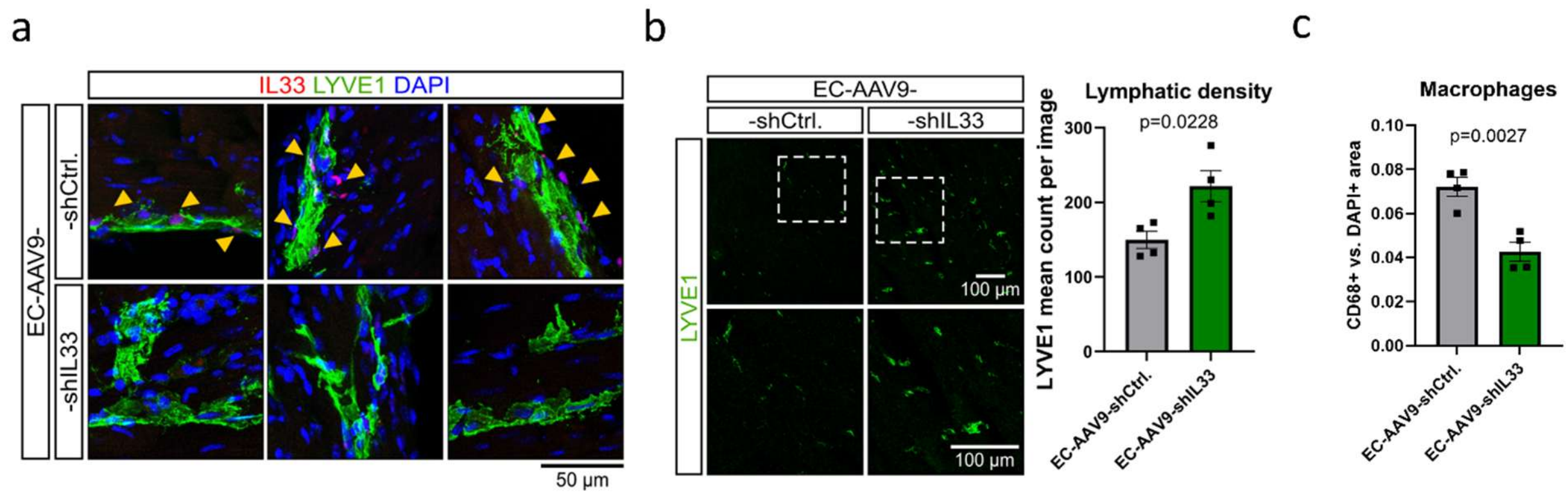


**Figure for the editors 1:** Repressing IL33 rescues lymphatic density in aged hearts. Modified AAV9 were i.v. injected to 18-month-old male mice to induce the expression of shRNA targeting Il33 (EC-AAV9-shIL33). A non-targeting shRNA served as control (EC-AAV9-shCtrl.). Four weeks after injection, hearts were assessed histologically (n=4). Panel a shows representative images of IL33 staining in cardiac lymphatics. DAPI was used as counter stain. Panel b shows LYVE1 staining to quantify lymphatic density. Panel c shows the quantification of macrophages in the heart (n=4). P-value was calculated using the unpaired, student's t-test.

2. Please provide a clear in vivo validation of the mechanistic significance of nuclear IL-33 in aged cardiac lymphatics in a lymphatic endothelial cell–specific IL–33 knockout model.

**Answer:** As the generation of an aged IL33-deletion line would cover more than two years, we decided to repress IL33 in wildtype aged mice using shRNAs delivered by AAV9 as demonstrated above.

2a. Please provide mechanistic insights into how nuclear IL 33 regulates lymphangiogenesis and how its nuclear expression is controlled.

**Answer:** According to the literature, IFN-γ induces *IL33* expression via the canonical JAK/STAT1 pathway in epithelial cells (PMID: 26986625). To determine whether this pathway also regulates *IL33* expression in lymphatic endothelial cells, we treated human dermal lymphatic endothelial cells (HDLECs) with the JAK inhibitor ruxolitinib (5 µM). JAK inhibition suppressed IFN-γ-induced *IL33* expression (**Figure 2.1a for the editors**). Likewise, siRNA-mediated *STAT1* silencing reduced IFN-γ-induced *IL33* expression in HDLECs (**Figure 2.1b for the editors**).

To elucidate how IFN-γ promotes nuclear IL33 expression, we focused on TFAP2A (Transcription Factor AP-2 Alpha). TFAP2A acts downstream of STAT1 (PMID: 16502414) and has been shown to indirectly regulate *IL33* splicing, resulting in the inclusion of exon 3, which contains the nuclear localization signal (PMID: 38456508). Indeed, siRNA-mediated *TFAP2A* silencing decreased *IL33* expression in IFN-γ-treated HDLECs (**Figure 2.1c for the editors**).

Collectively, these data indicate that IFN-γ activates the JAK/STAT1 pathway to promote *IL33* expression in lymphatic endothelial cells. STAT1 may further induce TFAP2A, thereby promoting the inclusion of exon 3 (nuclear IL33) in the *IL33* mRNA (**Figure 2.1d for the editors**).

While JAK/STAT1 and TFAP2A induce nuclear IL33 expression, we additionally included data demonstrating that overexpressing VEGFC represses nuclear IL33 in aged cardiac lymphatics (**Figure 5l of the revised manuscript**).

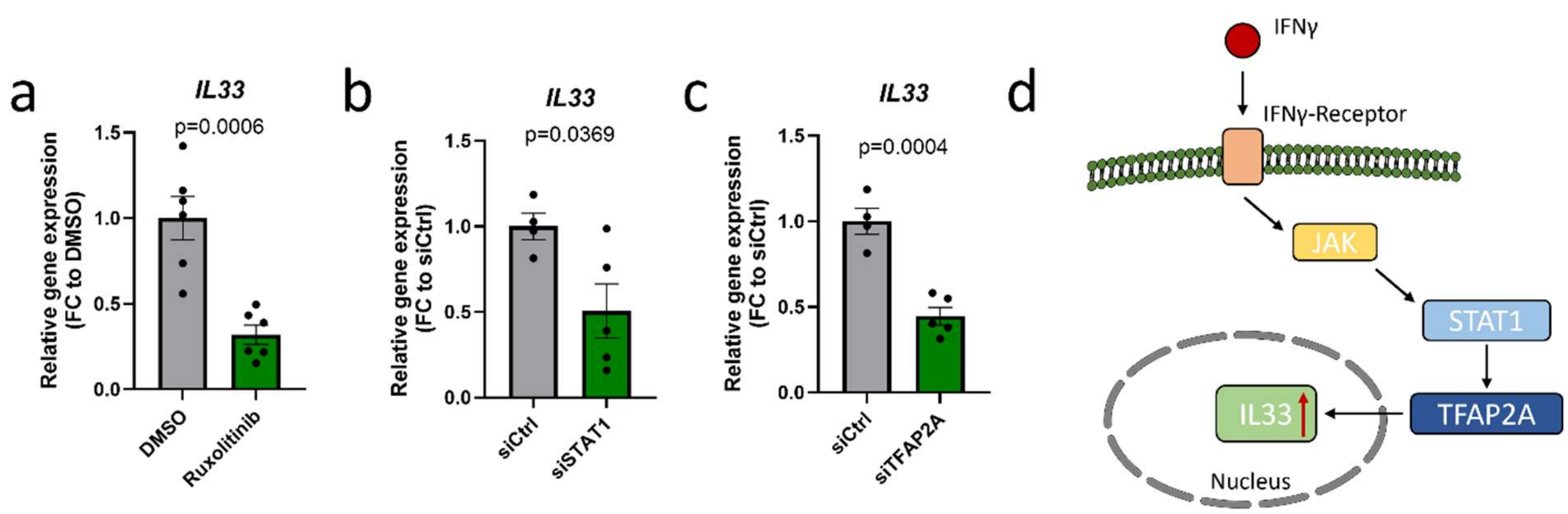


**Figure for the editors 2.1:** IFN-γ induces IL33 expression via JAK/STAT1/TFAP2A. **a.** HDLECs were treated with 5 µm ruxolitinib and 100 nM recombinant IFN-γ for 24h. *IL33* expression was determined by RT-qPCR versus RPL0 as housekeeper (n=6). **b.** *STAT1* was silenced by siRNA transfection in HDLECs. Two days after transfection, cells were treated for 24h with recombinant IFN-γ (100 nM). *IL33* expression was determined by RT-qPCR versus RPL0 as housekeeper (n=4 vs. n=5). **c.** *TFAP2A* was silenced by siRNA transfection in HDLECs. Two days after transfection, cells were treated for 24h with recombinant IFN-γ (100 nM). *IL33* expression was determined by RT-qPCR versus RPL0 as housekeeper (n=4 vs. n=5). **d.** Schematic representation of the putative pathway. P-value was calculated using the unpaired, student's t-test.

Regarding the comment on how nuclear IL33 may regulate lymphatic angiogenesis, we believe that our data are consistent with the literature showing that nuclear IL-33 is down-regulated during angiogenic activation (PMID: 18787100). In this context, nuclear IL-33 is proposed to function as a chromatin-associated transcriptional regulator that helps maintain a non-proliferative endothelial state, likely suppressing gene programs required for lymphatic proliferation and sprouting. This should be distinguished from extracellular IL-33, which can promote angiogenesis and lymphangiogenesis via ST2-dependent signaling. We therefore interpret our in vitro findings as reflecting the dominant nuclear function of IL33 in HDLECs (PMID: 19661270, PMID: 28878285, PMID: 18787100).

Importantly, our data also show that nuclear IL-33 is associated with increased lymphatic endothelial cell apoptosis and reduced proliferation. This suggests that nuclear IL-33 may impair lymphatic angiogenesis not only through a direct transcriptional mechanism but also indirectly by impairing cellular function: the loss of LECs via apoptosis and their diminished proliferative capacity would secondarily limit the formation

and maintenance of lymphatic capillaries. Thus, the reduction in lymphatic capillary density in aged hearts may reflect this cell-intrinsic loss-of-fitness phenotype driven by nuclear IL-33, while a more direct mechanistic contribution of nuclear IL-33 cannot yet be excluded.

In line with this, our newly generated bulk RNA sequencing data from IL33 overexpressing HDLECs demonstrate reduced expression of genes that are associated with mitosis and cell proliferation (**Figure 2.2a, b for the editors**). As such, the expression of the cell cycle inhibitor p21 is increased upon IL33 overexpression (**Figure 2.1c for the editors**).

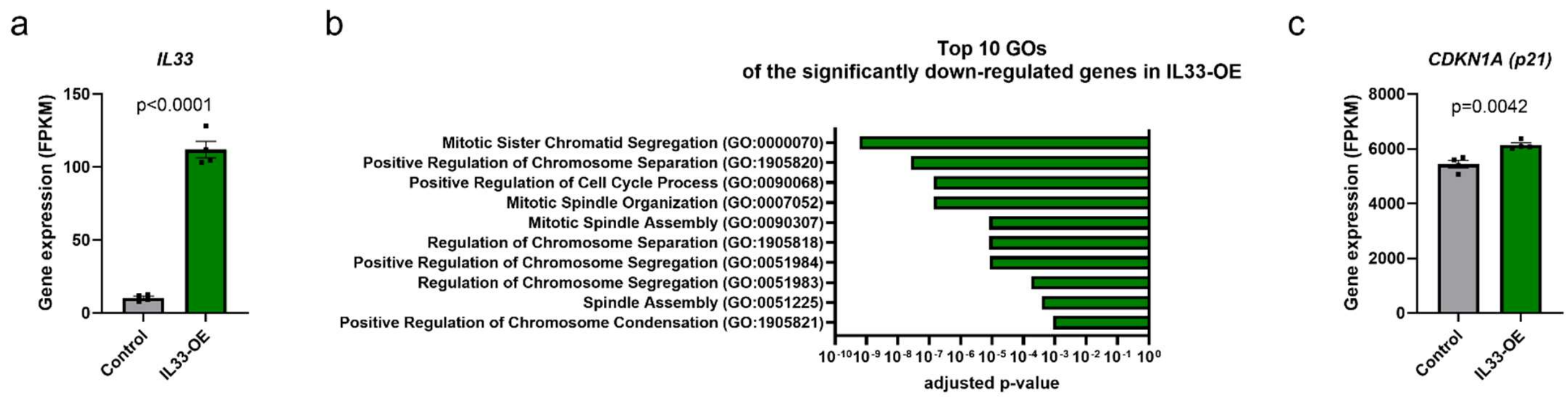


**Figure for the editors 2.2:** *IL33* overexpression reduces expression of genes that are associated with proliferation. *IL33* was overexpressed via lentiviruses in HDLECs. A control virus served as control (n=4 per group). RNA was extracted and mRNA sequencing was performed **a.** *IL33* expression in control and IL33 overexpressing HDLECs (n=4). **b.** Gene ontology analyses of genes that are significantly down-regulated upon *IL33* overexpression. **a.** *CDKN1A* expression in control and IL33 overexpressing HDLECs (n=4). P-value was calculated using the unpaired, student's t-test.

3. Please include some assessments of recognized cellular aging mechanisms, such as telomere shortening, genomic instability, mitochondrial dysfunction, oxidative damage, autophagy, or epigenetic alterations.

**Answer:** To address this concern, we performed bulk RNA sequencing on human dermal lymphatic endothelial cells (HDLECs) with and without IL33 overexpression, complemented by targeted functional assays for key aging hallmarks.

The assessment of bulk RNA sequencing data showed no profound changes in senescence core marker gene expressions. Only CDKN1A (encoding for p21) was slightly upregulated in IL33-overexpressing HDLECs (**Figure for the editors 3a**). In line with this, hallmarks of cellular senescence such as telomer length and senescence-associated β-galactosidase activity remained unchanged, too (**Figure for the editors 3b, c**). DNA damage as assessed by TUNEL staining was also not induced by IL33 induction (**Figure for the editors 3d**).

To assess metabolic changes in IL33-overexpressing cells, we quantified mitochondria via TOMM20 staining which were not altered between control and IL33-overexpressing cells (**Figure for the editor 3e**). We further assessed whether IL33 may affect autophagy. Autophagy-related genes showed no differential regulation on RNA level (**Figure for the editors 3f**). Immunofluorescence for p62 and LC3B confirmed unaltered autophagic flux, both in full medium and starvation conditions (**Figure for the editors 3g**).

Together, these data demonstrate that IL33 does neither induce cellular senescence, nor regulates mitochondrial abundance or autophagy.

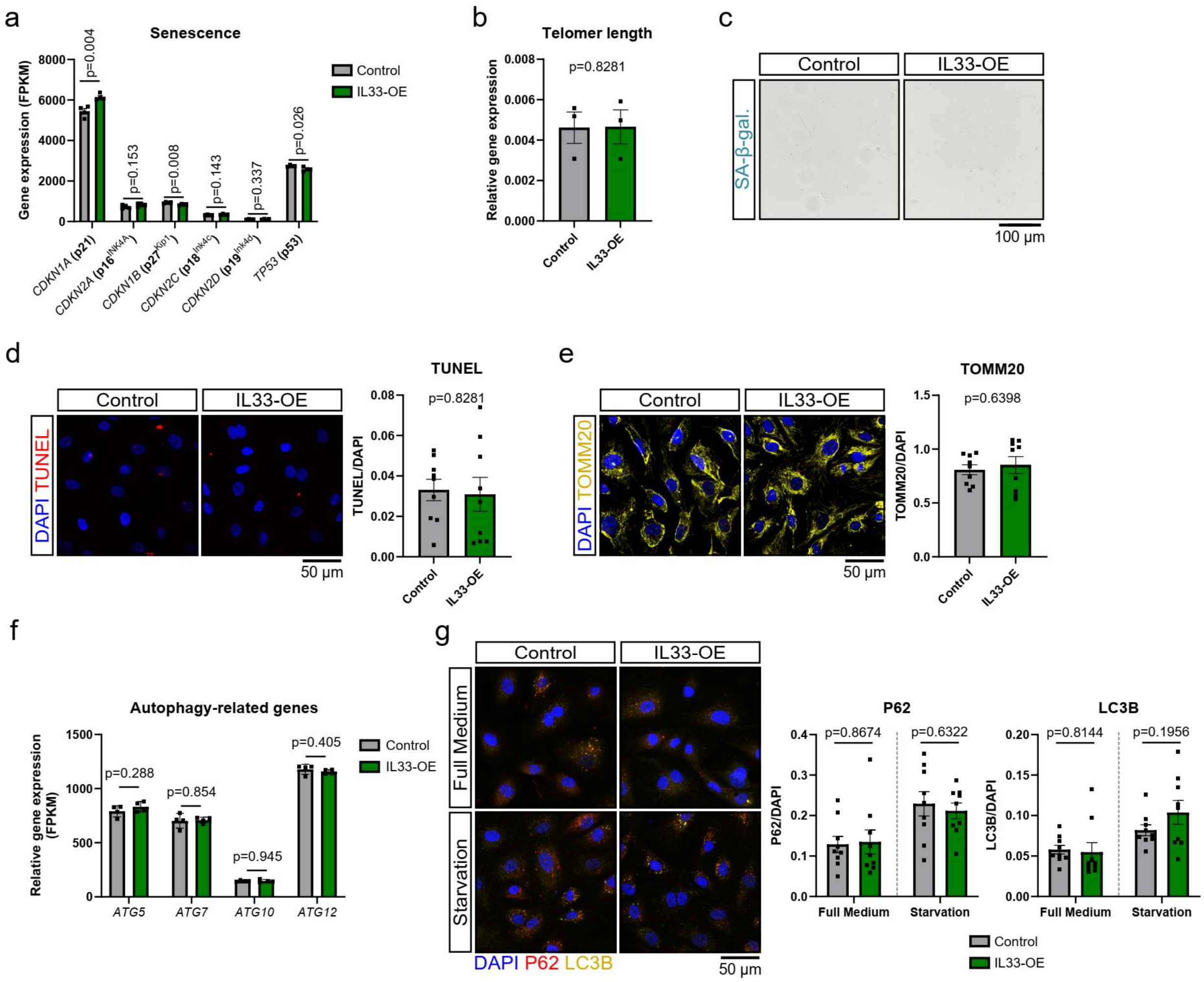


**Figure for the editors 3: Assessing hallmarks of ageing in IL33-OE HDLECs. a.** Bulk RNA sequencing of IL33-overexpressing (OE) vs. control HDLECs (n=4) showing the expression of senescence marker genes. **b.** Quantification of telomer length by quantitative PCR in IL33-overexpressing (OE) vs. control HDLECs (n=3). **c.** Representative images of senescence-associated β-galactosidase staining in control vs. IL33-OE HDLECs (n=3). **d.** TUNEL staining in IL33-overexpressing (OE) vs. control HDLECs (n=9). TUNEL area was quantified and normalized to DAPI area. **e.** TOMM20 staining in IL33-overexpressing (OE) vs. control HDLECs (n=9). TOMM20 area was quantified and normalized to DAPI area. **f.** Bulk RNA sequencing of IL33-overexpressing (OE) vs. control HDLECs (n=4) showing the expression of autophagy-related genes. **g.** P62 and LC3B stainings in IL33-overexpressing (OE) vs. control HDLECs in fully supplemented medium and starvation medium (n=9). P62 and LC3B area was quantified and normalized to DAPI area respectively. Data normally distributed and p-values were calculated using an unpaired, two-sided t-test.

4. Please increase the number of mice included in the study.

**Answer:** In our study, we analyzed lymphatic changes (and the potential consequences) across several age groups, resulting in a cumulative sample size that ensures solid statistical validity. Furthermore, we employed two independent models to mimic lymphatic impairment in young mice, both yielding consistent results that confirm the robustness of our findings.

In accordance with the principles of animal welfare and the 3Rs (Replacement, Reduction, and Refinement), we carefully balanced scientific rigor with ethical considerations to minimize the total number of animals used.

Nevertheless, as requested by the reviewers, we have increased the number of mice for the lymphatic size assessment (**Figure 1e of the revised manuscript**), for the basic aged heart (**Figure 2m-p of the revised manuscript**) and in the VEGFC rescue experiments (**Figure 5l, m, n, o of the revised manuscript**). The additional data fully corroborate our initial conclusions and further substantiate the reproducibility of our results.

**Response to reviewers' comments:**

**Reviewer #1:** This study demonstrates that a loss of lymphatic vessels can be observed during aging in mice, associated also with changes in lymphatic vessel architecture. These findings correlated with an increase in immune cells, including T cells and macrophages in the heart, an increase in water retention, fibrinogen accumulation, and fibrosis. Deletion of VEGFR3 in LECS in young mice reduced lymphatic vessel counts, and was again associated with an increase in macrophages and fibrinogen, and similar findings were observed upon overexpression of sFLT4, confirming that VEGFR3 plays a critical role in lymphatic vessel formation and in the heart has a role in clearing inflammation and edema. The authors then identify upregulation of IL-33 in aged LECs, and in vitro show that IL-33 regulates LEC proliferation and apoptosis, and can be induced by IFNg, expressed by T cells in the heart. VEGFC treatment was shown to reduce IL-33 expression. Finally, the AAV-induced VEGFC overexpression increased lymphatic vessel counts and reduced macrophage accumulation in aged mice.

The loss of lymphatic vessels, which may contribute to age-associated deterioration in cardiac function, and findings related to IL-33, are novel and interesting.

1. In sections through the heart (Fig. 1b-d, 2a), LYVE1 staining may have also comprised Lyve1+ macrophages. Co-staining of macrophages should be performed to exclude macrophage "contamination" of lymphatic analyses.

**Answer:** The reviewer addressed a well-taken point. To circumvent this issue, we used whole mount light sheet microscopy imaging of LYVE1-stained hearts, which showed vascular LYVE1 structures. We further used PDPN staining and VEGFR3 Western Blot analyses to strengthen our observation. As the reviewer is correct and tissue-resident macrophages do express LYVE1, we additionally performed LYVE1 and CD68 fluorescence staining on n=4 young and old mouse heart sections (**Fig. for reviewer 1.1**). To exclude LYVE1+ macrophages, we quantified CD68-negative LYVE1+ cells, which were less abundant in the aged heart section, thus confirming our findings.

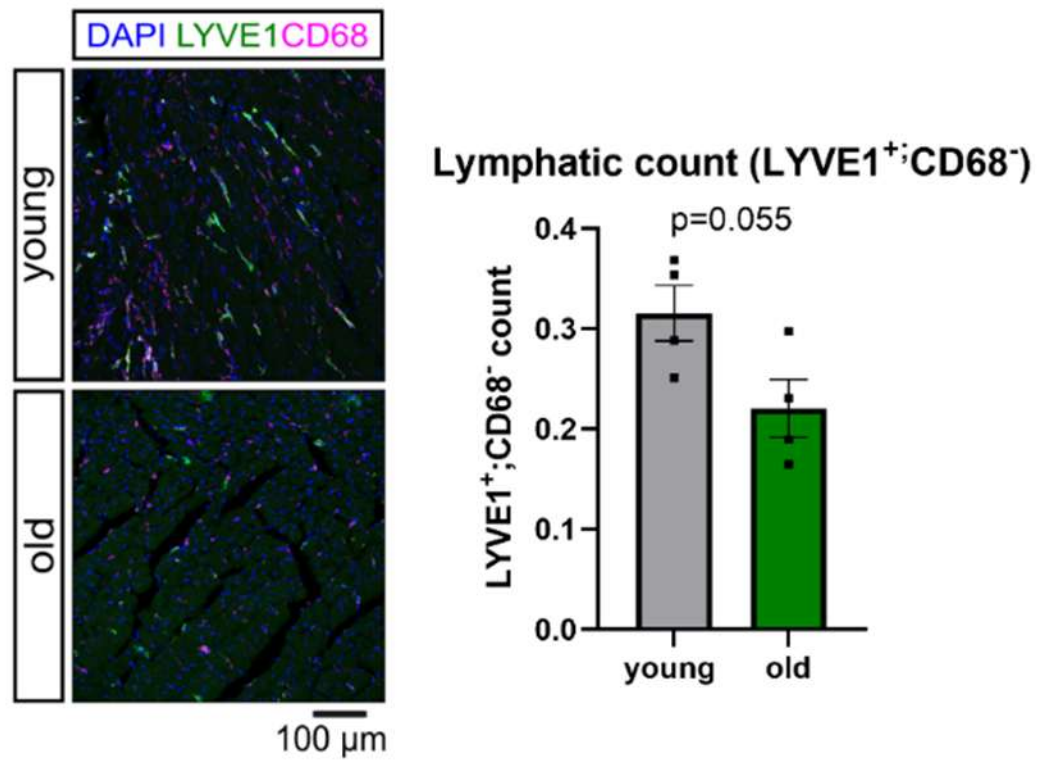


**Figure for the reviewers 1.1:** LYVE1, CD68 and DAPI staining in heart section from young versus old mice (n=4). Data are normally distributed and p-value was calculated using an unpaired, two-sided t-test.

2. It is not entirely clear how sections of the sub-endocardial area were generated; also, the exact location of sections in the heart in Figure 1c is unclear.

**Answer:** We apologize for being unclear. To specify the imaging strategy, we provide a schematic illustration in **Figure for the reviewers 1.2**. We cut the fixed whole heart transversally as indicated in the figure below. The black dashed lines indicate the area of the heart that was used. We generated 50-µm thick cross-sections and took five randomly chosen images (red boxes) in the myocardium close to the endocardium.

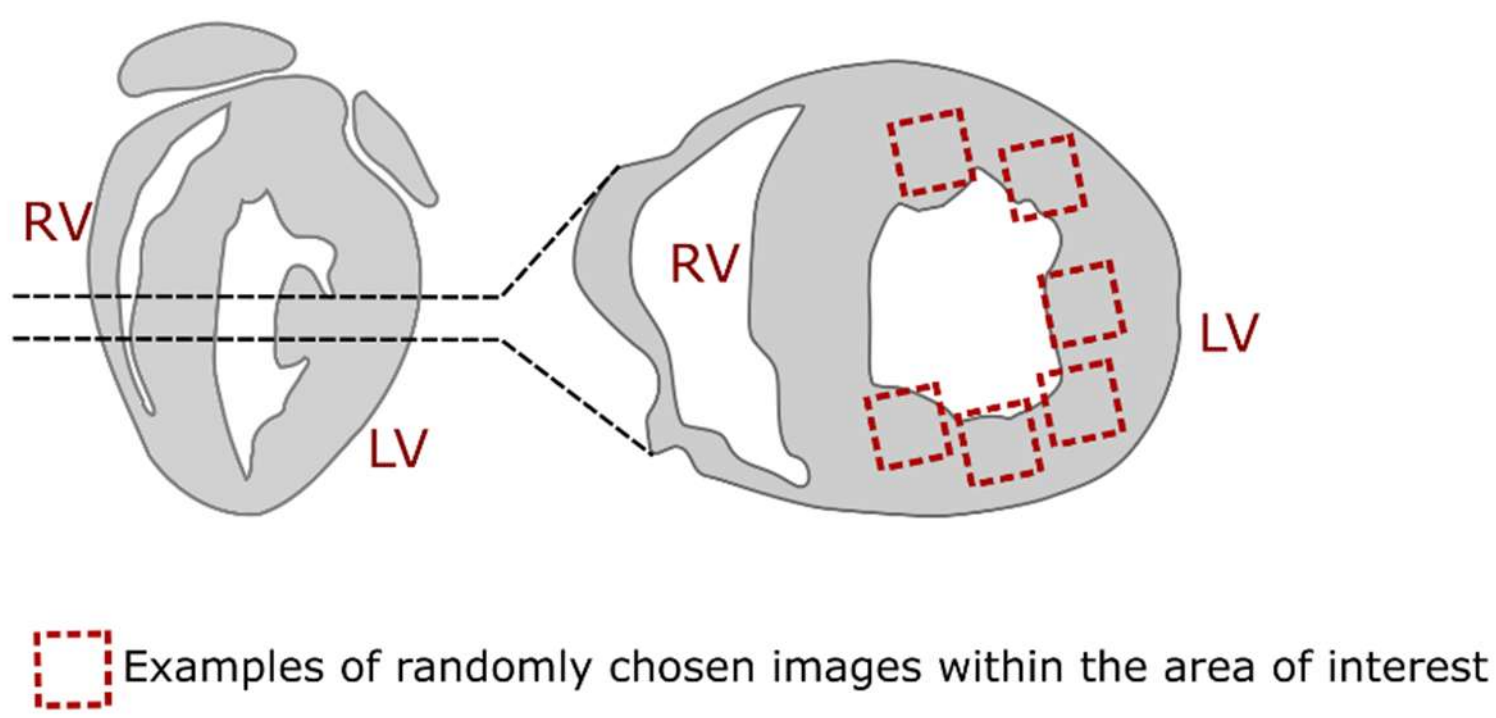


**Figure for the reviewers 1.2:** Schematic representation of the imaging strategy to quantify lymphatic capillaries in the sub-endocardial area.

3. Zipper and Button-junctions were counted per image; it may be more meaningful to assess these relative to the lymphatic vessel area.

**Answer:** In the initial draft of the manuscript the comparison was made between button vs zipper count in each of the cohort to report the difference between their counts, which doesn't require normalization. However, the reviewer raises an important point. Hence, we normalized the junction counts to the respective LYVE1+ area. As shown in **Figure for the reviewers 1.3**, normalizing the junction count to the vessel area does not change our initial observation.

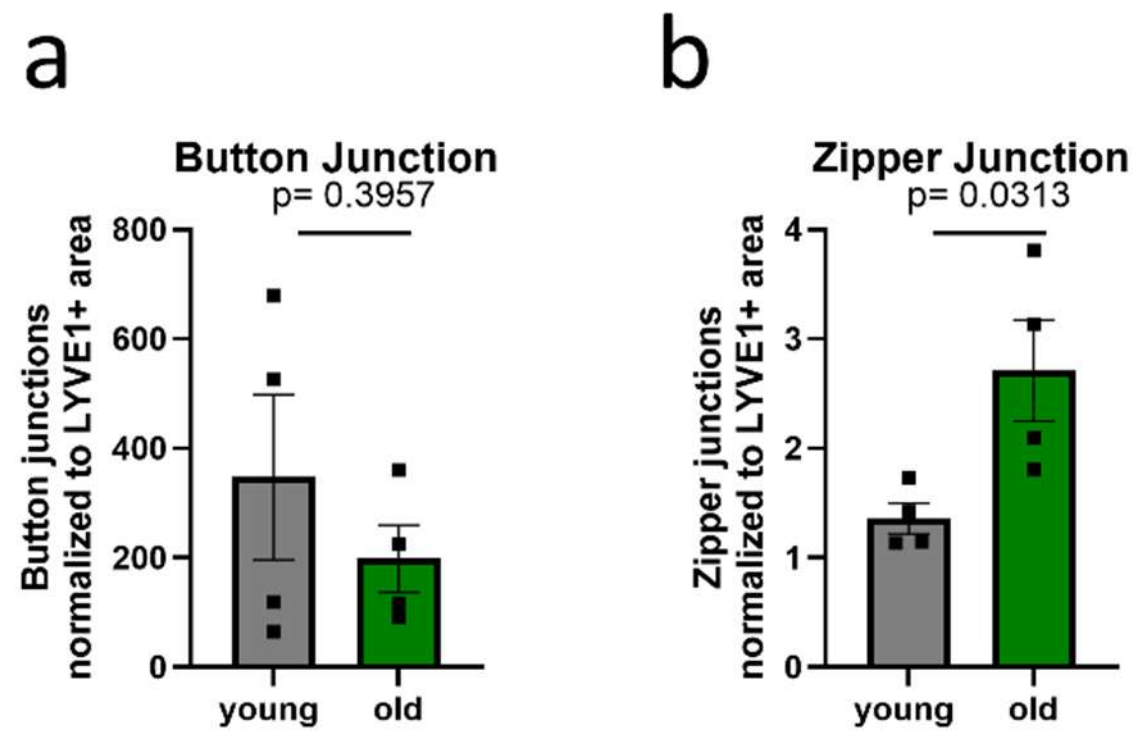


**Figure for the reviewers 1.3:** Junction counts for button (a) and zipper (b) junctions in young and old mouse hearts were normalized to the respective vessel area (n=4). Data are Gaussian distributed and p-values were calculated using the paired, student's T-test.

4. In general, it has previously been shown that only LYVE-1 + precollector-like vessels (> 20 µm in diameter) express podoplanin. Lymphatic podoplanin expression is less frequent in midmyocardial areas, and the vast majority of LYVE-1+ lymphatic capillaries (>80%) are small (diameter <5 µm) and negative for podoplanin. The location of the lymphatics measured in this study and their coexpression with PDPN require clarification, in particular, as sometimes vessels are only quantified using LYVE1 or PDPN.

**Answer:** We agree with the reviewer that LYVE1 and PDPN expression is dependent on the type of lymphatics. In the present manuscript, we have not shown LYVE1 and PDPN double-positive areas predominantly labeling lymphatics of within the epicardial regions, and show that they are also decline with age. Those results were shown in separate stainings. We apologize for a writing mistake where we accidently wrote PDPN+LYVE1+ vessels. We meant PDPN+ and LYVE1+ vessels separately. This mistake has been corrected in the revised manuscript.

5. Also, the topography of the other changes described needs to be described in more detail. E.g., for quantification of macrophages, which region of the heart has been analyzed.

**Answer:** We images the macrophage density in the myocardium of the left ventricular wall. To specify the imaging strategy, we provide a schematic illustration in **Figure for the reviewers 1.5**. We cut the fixed whole heart transversally as indicated in the figure below. The black dashed lines indicate the area of the heart that was used. We generated 50-µm thick cross-sections and took five randomly chosen images (yellow boxes) within the left ventricular wall as indicated by the red dashed line.

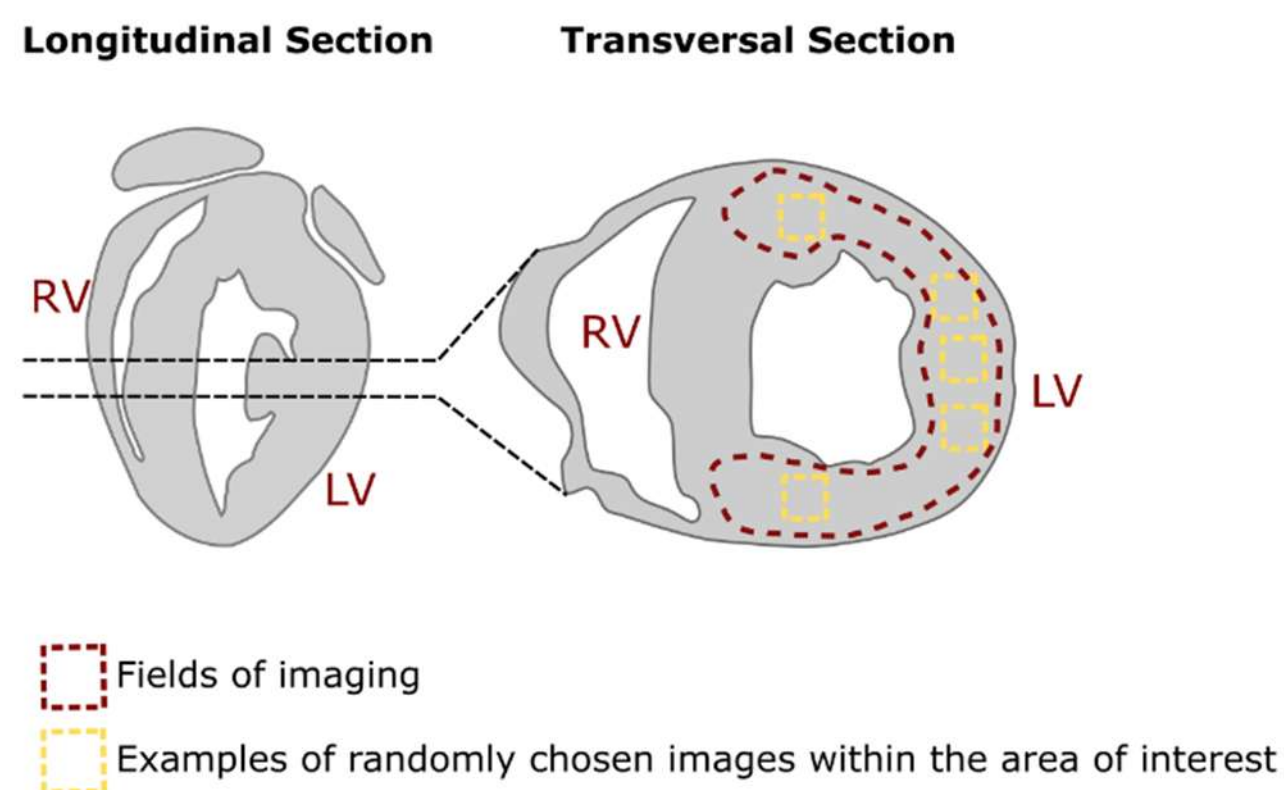


**Figure for the reviewers 1.5:** Schematic representation of the imaging strategy to quantify macrophages.

6. Macrophages are represented relative to the DAPI+ area, whereas T cells and DC were quantified per mg LV tissue. Please clarify.

**Answer:** We apologize for the lack of clarity in the initial manuscript. To quantify macrophages in young and aged mouse hearts, we performed CD68 immunofluorescence staining and normalized the $CD68^+$ area to the total $DAPI^+$ area (the respective area of assessment is indicated in Figure 1.5 for Reviewer 1). Histological detection of T cells proved difficult due to their overall low abundance. In Figure 5e of the initial version, $CD3^+$ T cells were primarily observed adjacent to larger lymphatic vessels in aged hearts, whereas almost no T cells were detected in young hearts. Therefore, we used flow cytometry (FACS) (normalized to mg LV) to quantify T cells, neutrophils, and dendritic cells, as shown in Figure 2.

We used the left ventricles from mice at 3, 16 and 22 months of age, weighted each tissue and homogenized them. Cardiac immune cells were defined as: $Ly6C^+$ monocytes ($CD45^{high}$, $CD11b^{high}$, $Ly6G^{low}$, $F4/80^{low}$, $Ly6C^{high}$), neutrophils ($CD45^{high}$, $CD11b^{high}$, $Ly6G^{high}$), T cells ($CD45^{high}$, $CD11b^{low}$, $CD90.2^{high}$), dendritic cells ($CD45^{high}$, $CD11b^{high}$, $Ly6G^{low}$, $F4/80^{low}$, $Ly6C^{low}$, $CD11c^{high}$, $MHC\text{-}II^{high}$).

To determine absolute cell numbers, cell suspensions were transferred to BD Trucount™ Tubes (Cat. No. 340334; BD Biosciences) prior to flow cytometric analysis. These tubes contain a predefined number of fluorescent beads, enabling calculation of absolute cell counts per sample and normalization to tissue weight (cells per mg tissue). This methodological detail has now been explicitly included in the revised Methods section.

7. Representative images of Fibrinogen should be shown (Fig. 2i).

**Answer:** Please excuse the omission. Representative images are shown in the revised manuscript.

8. Cardiac hypertrophy is mentioned in the text but not shown.

**Answer:** We apologize for being unclear. We referred to Tamiato et al. Circ Res 2024 and hence did not show cardiac hypertrophy in the present study. However, we now included data on cardiac hypertrophy in the revised manuscript (Fig. 2m, n).

9. It would be informative to also include evaluations of the heart function of mice during aging and in $Vegfr3^{\Delta LEC}$ mice.

**Answer:** We agree that these pieces of information are missing. We included echocardiography data of young versus aged mice (3 vs. 18 months) in Fig. 2n-p of the revised manuscript.

In the *Vegfr3*$^{i\Delta LEC}$ mouse model, we performed MRI before and 8 weeks after tamoxifen injection (**Figure for the reviewers 1.9a, b**). in this study, because of technical reasons (mice were kept in another facility), we were not able to perform echocardiography on these animals. Longitudinal MRI showed no changes in systolic function (ejection fraction and end-systolic mass) but suggested impaired diastolic function as shown by elevated end-diastolic mass (**Figure for the reviewers 1.9c, d**). As seen by visual inspection of the MRI scans, the left atrium is enlarged after lymphatic *Vegfr3*-deletion (**Figure for the reviewers 1.9c**). Together with the elevated end-diastolic mass, this might be indicative for diastolic dysfunction. These data are consistent with the echocardiography data from the sFlt4-AAV9 mice and aged mice and overall demonstrate an age-like diastolic dysfunction in adult mice after impairing the cardiac lymphatic system.

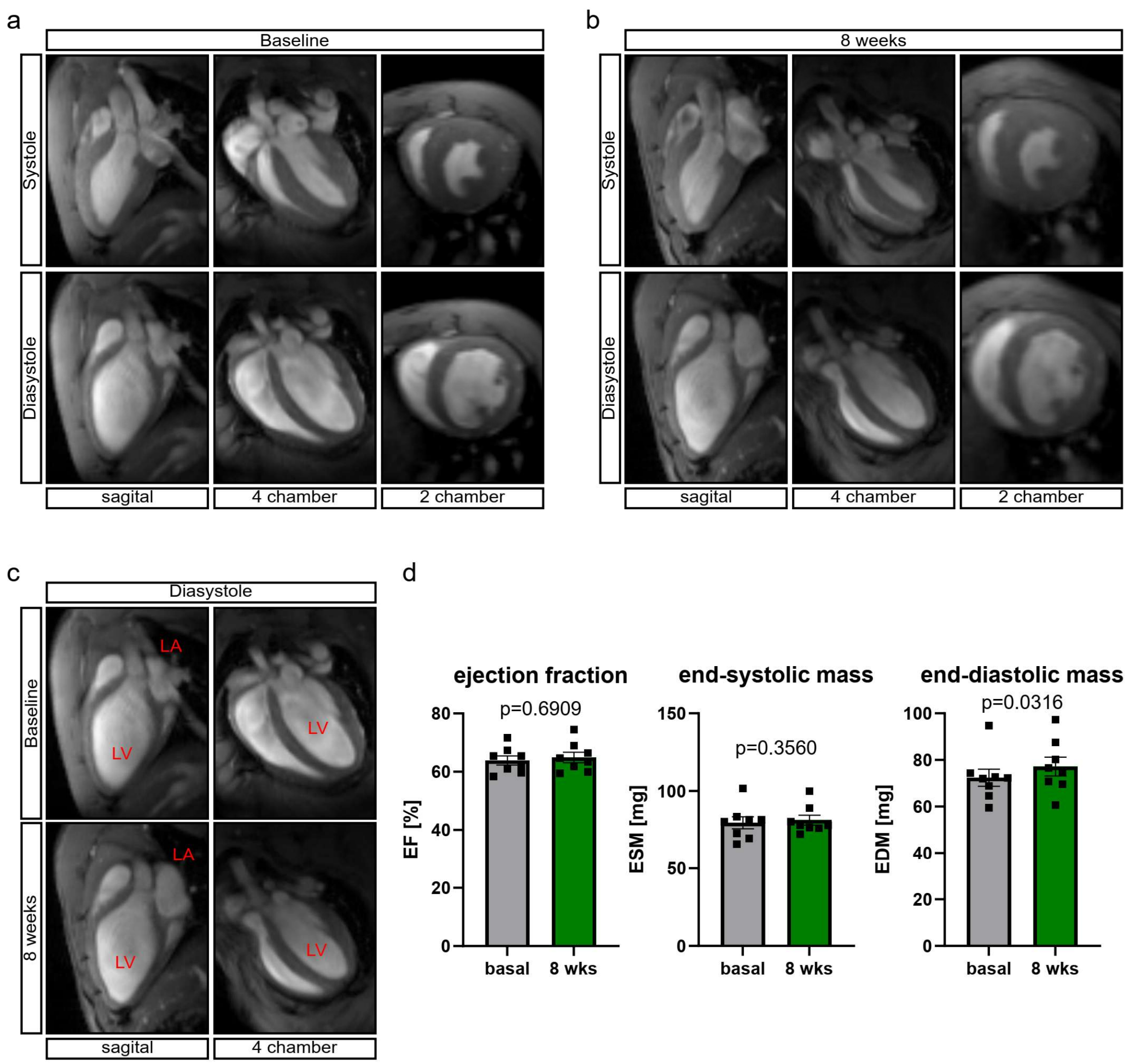


**Figure for the reviewers 1.9: Lymphatic-specific *Vegfr3*-deletion impairs diastolic function.** Magnet resonance imaging (MRI) of Vegfr3iΔLEC mice before (**a**) and eight weeks after tamoxifen injection (**b**). Shown are scans at systole and diastole in sagittal, 4-chamber and 2-chamber view. Comparison between baseline

and eight weeks post tamoxifen are shown in (**c**) while d shows the quantification of ejection fraction, end-systolic and end-diastolic mass (n=8). Data are Gaussian distributed and p-values were calculated using the paired, student's T-test.

10. The increase in diastolic function in hearts after sFlt4 overexpression is interesting; what could be the underlying reason for this finding?

Answer: The elevation of the E/E' ratio reflects diastolic dysfunction and indeed, the reviewer raised an important point. Neither cardiac fibrosis nor cardiac hypertrophy were observed in these mice. However, inflammation (as assessed by elevated macrophage levels) and edema (fibrinogen was used as surrogate) might impair diastolic function. One additionally may raise the hypothesis that paracrine signals from the lymphatics ("lymphangiocrines") might be required to maintain cardiomyocyte function. However, this has not been reported so far and would deserve further in-depth studies.

11. Data on cardiomyocyte size in sFlt4 overexpression is duplicated (Fig. 3m, and Supp. Fig. 3).

Answer: We thank the reviewer for the thorough reading. Indeed, the data were duplicated. Because of space issues, we did not show the representative WGA fluorescence staining in main figure 3. Hence, we shifted these to suppl figure 3 and included the quantification in sake of completeness.

As this might be confusing to the reader, we only referred to Suppl. Fig. 3 and removed the data from Fig. 3m in the revised manuscript.

12. It should be demonstrated that the nuclear isoform of IL33 protein is induced in lymphatics in vitro upon OE and siIL33 treatment.

Answer: We agree with the reviewer and performed a PCR to detect the exon 3 of IL33, which codes for the nuclear localization signal (NLS). As shown by **Figure for the reviewers 1.12a, b**, IL33-overexpression does increase the expression of the nuclear form, while siIL33 transfection reduces the expression. We further included an immune fluorescence staining demonstrating the nuclear expression of IL33 after lentiviral overexpression in **Figure for the reviewers 1.12c** (yellow arrow heads indicate examples of nuclear IL33).

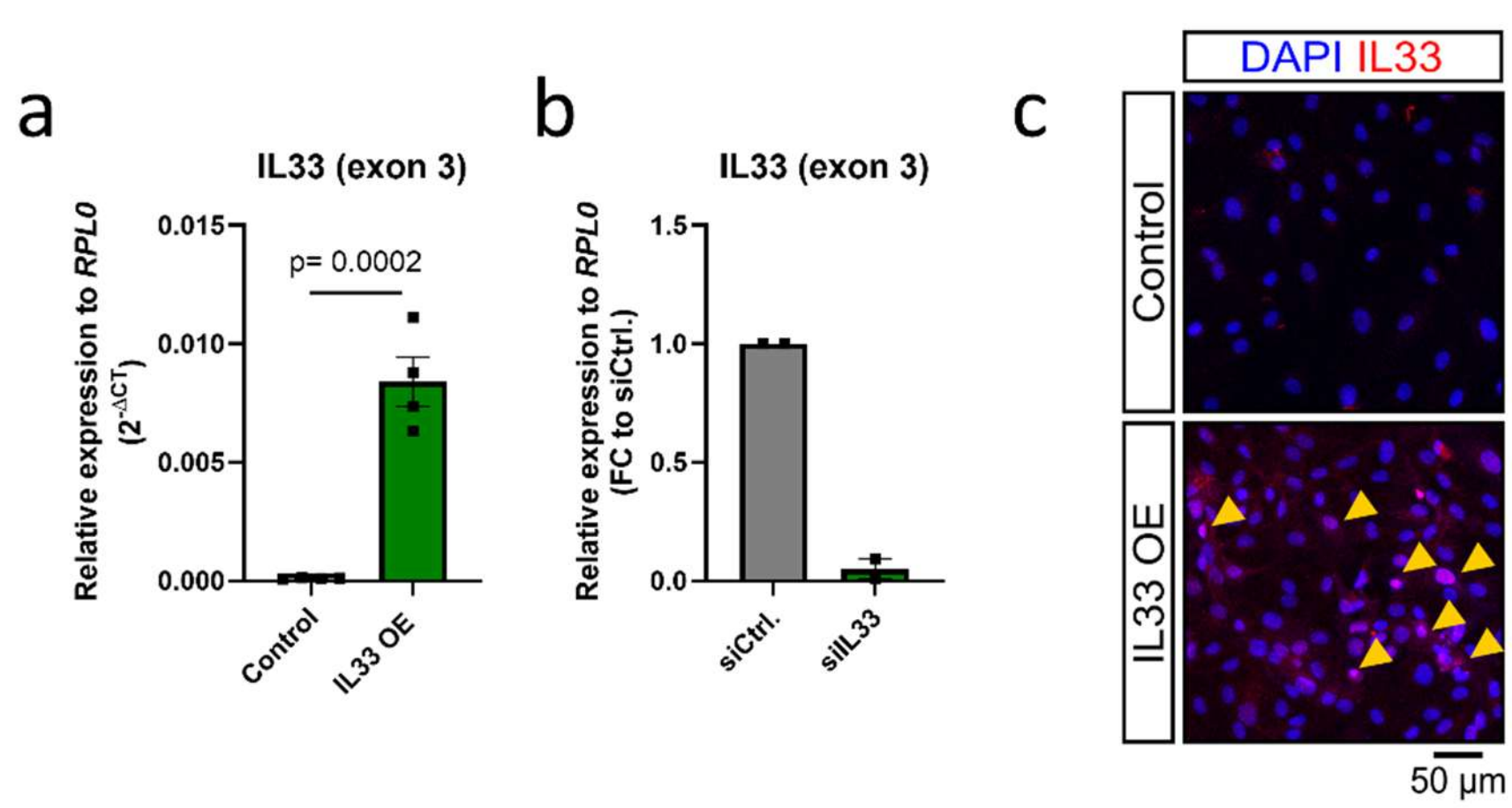


**Figure for the reviewers 1.12: Manipulation of the nuclear form of IL33. a** Shown is the expression of exon 3 of IL33 that codes for the nuclear localization signal. Lentiviral IL33-overexpression of control cells were used (n=4). **b** Expression of exon 3 in HDLEC upon siIL33 versus siCtrl. Transfection (n=2). **c.** Representative staining of IL33 after IL33-overexpression. Arrow heads indicate the nuclear localization. P-value was calculated using the unpaired, student's t-test.

13. Are levels of IL-33 altered in mice treated with AAV9-Vegfc FL?

**Answer:** Indeed, overexpressing *Vegfc* in aged mice reduces the expression of IL33 in cardiac lymphatics as shown by **Figure for the reviewers 1.13**. These data are now included in the revised manuscript in **Fig. 5l**.

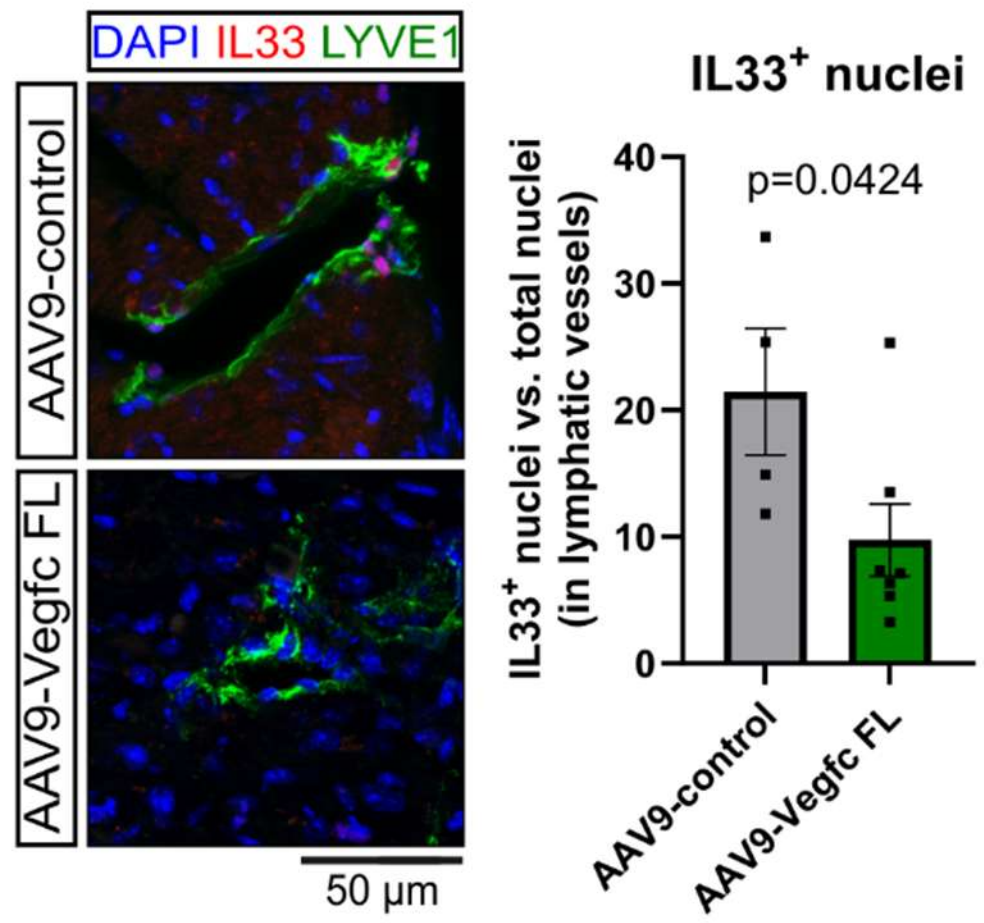


**Figure for the reviewers 1.13: *Vegfc* overexpression reduces IL33 expression in aged cardiac lymphatic.** AAV9-Vegfc FL (n=7) or AAV9-control (n=4) were i.p. injected to 18-month-old mice. Eight weeks after injection, hearts were histologically assessed for lymphatic IL33 expression. Mean IL33+ nuclei were quantified (%) per lymphatic vessel. Data are not normally distributed and p-value was calculated using the unpaired, Mann-Whitney test.

14. Effects of IL-33 have been studied in vitro using human dermal lymphatic endothelial cells; whether lymphatic vessel rarefaction in vivo is connected to IL-33 remains unclear. This is of particular relevance, as VEGF-C levels seem to decrease in aging hearts, and overexpression of this cytokine increases lymphatic vessel counts also in old mice, a response expected from previous studies, but possibly unrelated to IL-33.

**Answer:** To support the hypothesis that IL33-mediates age-related lymphatic regression, we now demonstrate that VEGFC FL overexpression in aged mice reduce the IL33 expression in cardiac lymphatics (**main Figure 5l of the revised manuscript**). We further generated an endothelial-targeted AAV9 system to repress IL33 expression by overexpressed shRNAs under the endoglin promotor targeting IL33. We demonstrate that IL33 expression is reduced in cardiac lymphatics of aged mice (**Figure for the reviewers 1.14a**). Silencing IL33 resulted in elevated lymphatic capillary density and reduces macrophage abundance (**Figure for the reviewers 1.14b, c**).

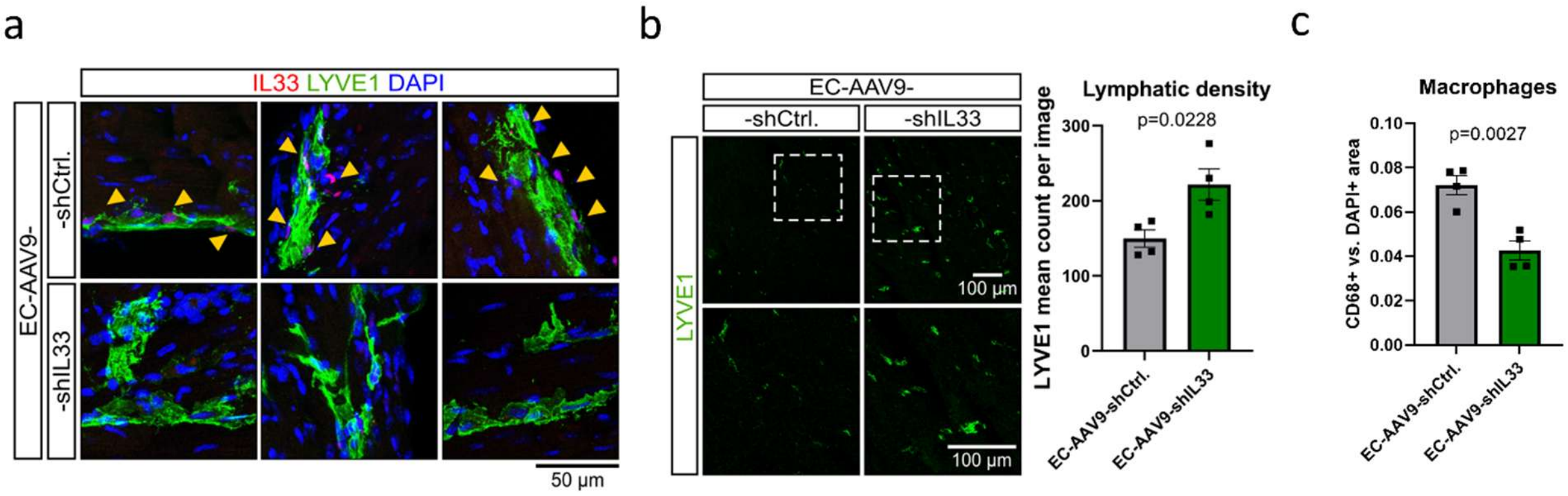


**Figure for the reviewers 1.14:** Repressing IL33 rescues lymphatic density in aged hearts. Modified AAV9 were i.v. injected to 18-month-old male mice to induce the expression of shRNA targeting Il33 (EC-AAV9-shIL33). A non-targeting shRNA served as control (EC-AAV9-shCtrl.). Four weeks after injection, hearts were assessed histologically (n=4). Panel a shows representative images of IL33 staining in cardiac lymphatics. DAPI was used as counter stain. Panel b shows LYVE1 staining to quantify lymphatic density. Panel c shows the quantification of macrophages in the heart (n=4). P-value was calculated using the unpaired, student's t-test.

## 15. Is the decline in macrophages in the mediastinal lymph node specific, or are similar shifts observed in other lymph nodes, as well?

**Answer:** The reviewer raised an important point. To clarify this, we assessed cervical and inguinal lymph nodes from young (3 months) and old (22 months) mice. Interestingly, both lymph nodes revealed a tendency towards more CD68+ macrophages (**Figure for the reviewers 1.15**).

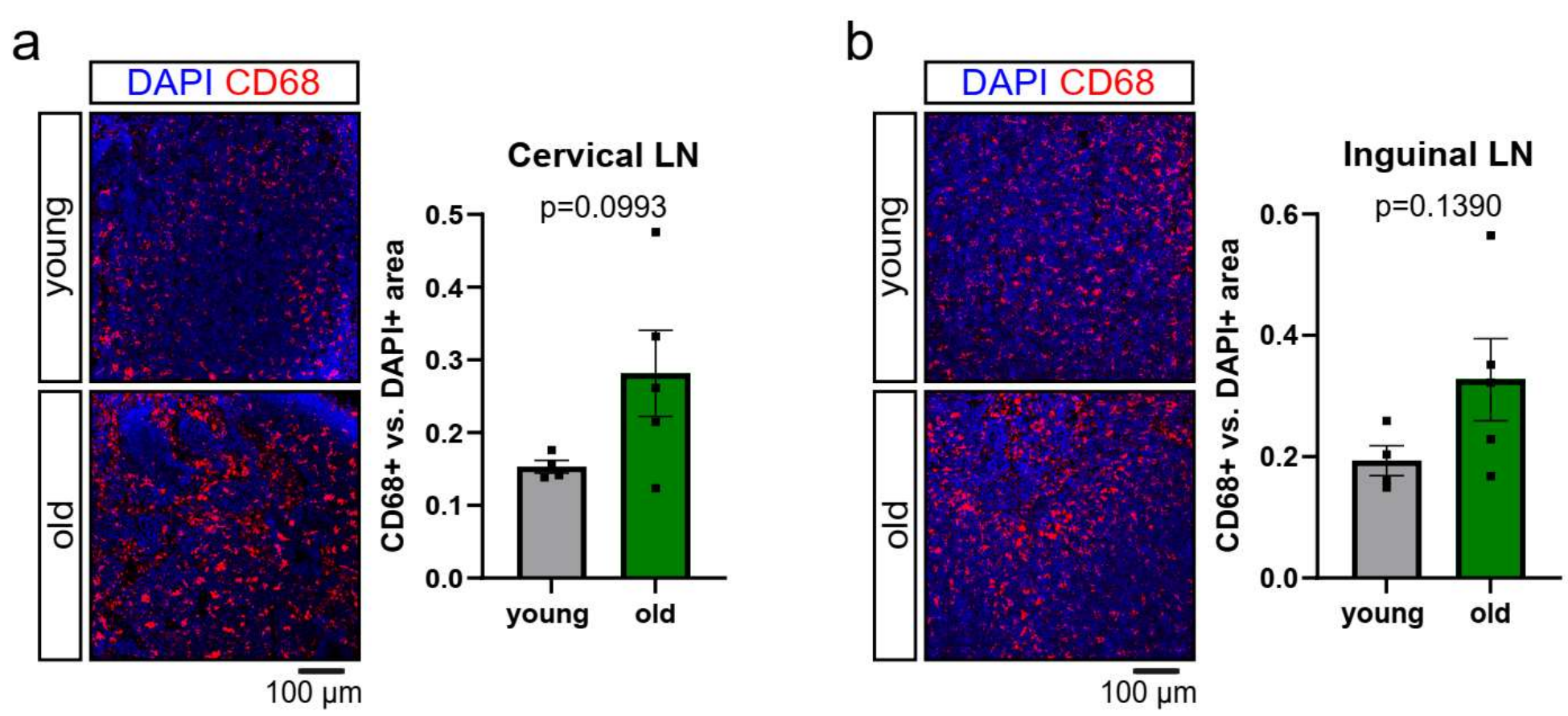


**Figure for the reviewers 1.15: Macrophage density does not decline in aged cervical and inguinal lymph nodes.** Lymph nodes (LN) were isolated from young (3 months, n=4) and old (22 months, n=5) mice and stained for CD68 and DAPI. Panel **a.** shows the cervical LN while **b.** shows inguinal LN. P-value was calculated using the unpaired, student's t-test.

## 16. The number of mice is mostly very low.

**Answer:** We thank the reviewer for the valuable suggestion to increase the number of animals. In our study, we analyzed lymphatic changes (and the potential consequences) across several age groups, resulting in a cumulative sample size that ensures solid statistical validity. Furthermore, we employed two independent models to mimic lymphatic impairment in young mice, both yielding consistent results that confirm the robustness of our findings.

In accordance with the principles of animal welfare and the 3Rs (Replacement, Reduction, and Refinement), we carefully balanced scientific rigor with ethical considerations to minimize the total number of animals used.

Nevertheless, as requested, we have increased the number of mice for the lymphatic size assessment (**Figure 1e of the revised manuscript**), the general aged mouse heart characterization (**Figure 2m-p of the revised manuscript**) and in the VEGFC rescue experiments (**Figure 5 of the revised manuscript**). The additional data fully corroborate our initial conclusions and further substantiate the reproducibility of our results.

**Reviewer #2:** Wagner et al. have addressed an important and clinically-relevant question of whether and how aging influences cardiac lymphatic rarefaction and immune cells interactions. Using cohorts of young and old mice, the authors show reduced lymphatic density with aging which is accompanied by increased tissue immune cell infiltration and myocardial edema. The most novel finding of the study is a selective upregulation of nuclear IL33 which they show using in vitro cell culture studies, stimulates lymphatic endothelial cell death and junctional remodeling. As expected, VegfC overexpression in animal models drives cardiac lymphatic growth, thereby restoring the aging effects on cardiac lymphatics, but the ultimate effects on nIL33 or cardiac function are not investigated. Overall, this is a timely study with compelling preliminary findings and a plausible mechanistic linking of cardiac lymphatic rarefaction with inflammation and aging. Addressing the following comments would improve the impact of the study.

1. An overall comment and area for improvement is related to poor image quality. Many of the images rely on visualizing a dim green signal on a black background. Even with optical zoom, the staining is barely visible.

**Answer:** We apologize for the quality. As changing the color did not improve the visibility, we decided to add zoom-in images to every overview image. Please see the revised manuscript.

2. The finding of increased lymphatic diameter, Fig 1e, is difficult to interpret and based on the limited data presented an over-interpretation. In the representative "old" image, what do the authors define as the lumen and wall of the labeled structures? Do the data points in the graph represent a single vessel measurement, in 3X different animals? It appears that there are only 2X data points for the "young" analysis, which is too low to make rigorous conclusions. In addition, the finding of 15% increased diameters in old hearts is not evident across other images and data.

**Answer:** We apologize for being unclear in the initial manuscript. We initially showed three biological replicates (three animals) per group. Each data point was the mean of analysing multiple lymphatic tubes per animal. We added three further biological replicates to enhance the statistical power and indicated the lumen that was measured in the representative image. Please see main **Figure 1e** of the revised manuscript.

3. The data in Figure 1h-k on button versus zipper junctions is unconvincing. The representative image of a young lymphatic, which is presumably one of the better selected images, is uninterpretable.

**Answer:** The reviewer addressed an important point. We included further representative images in **Suppl. Figure 1d of the revised manuscript**.

4. Fig 1.c,d shows sex differences in aging phenotypes, with males losing more Lyve1+ cells. Unless I missed it, the sex of the animals for analyses in the remainder of the study are not defined. This would be particularly relevant for analyses and methods that rely on Lyve1 positivity, like Figs. 2 and 3. Were sexes combined?

**Answer:** The reviewer raised an important point. Indeed, we demonstrated that cardiac lymphatics regress in both males and females. As the aged females are not constantly available, we used male mice throughout all AAV9 experiments. The lymphatic deletion of Vegfr3 was performed in female mice. We indicated the sex in the respective figure legends.

5. The Congo red data, Fig. 2j,k are not convincing. The staining image does not appear to reflect the quantified data. Please provide representative images and images for Sirius red.

**Answer:** We inserted zoom-in images and indicated plaque accumulation by black arrow heads to provide more appropriate images.

6. A confounding and technical limitation of the study is the isolation and analysis of Cdh5+ endothelial cells to perform gene expression analysis (Fig. 4), from which much of the mechanistic data is derived. The authors computationally enriched for lymphatic clusters based on prior datasets, but cardiac LECs should be isolated separately to provide more rigorous gene expression analysis of this population of cells. As currently written, the authors cannot claim these data or identified genes are derived from lymphatic endothelial cells.

**Answer:** We agree that isolating and profiling bona fide cardiac LECs represents the conceptual gold standard for mechanistic inference. Indeed, we initially tried to isolate cardiac LECs by using a Prox1-eGFP line. However, the low abundance of LECs in the adult mouse heart, together with the requirement for substantial cell numbers for robust bulk/single-cell RNA sequencing, posed a major technical limitation and made our initial attempts unpractical. As shown by flow cytometry of mouse heart homogenates in **Figure for the reviewers 2.6a**, the count of LYVE1+ cells (lymphatic endothelial cells and tissue-resident macrophages) are below 0.1%. Even enriching for non-immune cells (CD45-negative cells), LYVE1+ lymphatic endothelial cells make 0.22% of all cells.

Apart from this aspect, using a single marker for LECs might result in the loss of important LEC subpopulations. Within Cdh5+ compartment, we used established lymphatic marker signatures (e.g., *Prox1*, *Lyve1*, *Pdpn*, *Flt4*) to computationally define lymphatic (sub)clusters and derive lymphatic-biased transcriptional signatures. Indeed, we identified a clean lymphatic cluster (**Figure for the reviewers 2.6b**). Sub-clustering the lymphatic cluster allowed us to identify four distinct lymphatic clusters with uneven expression levels of *Lyve1* and *Pdpn* (**Figure for the reviewers 2.6c**). By using one specific lymphatic marker such as LYVE1, one would miss the $LYVE1^{low}$ or $LYVE1^{negative}$ lymphatic endothelial cells.

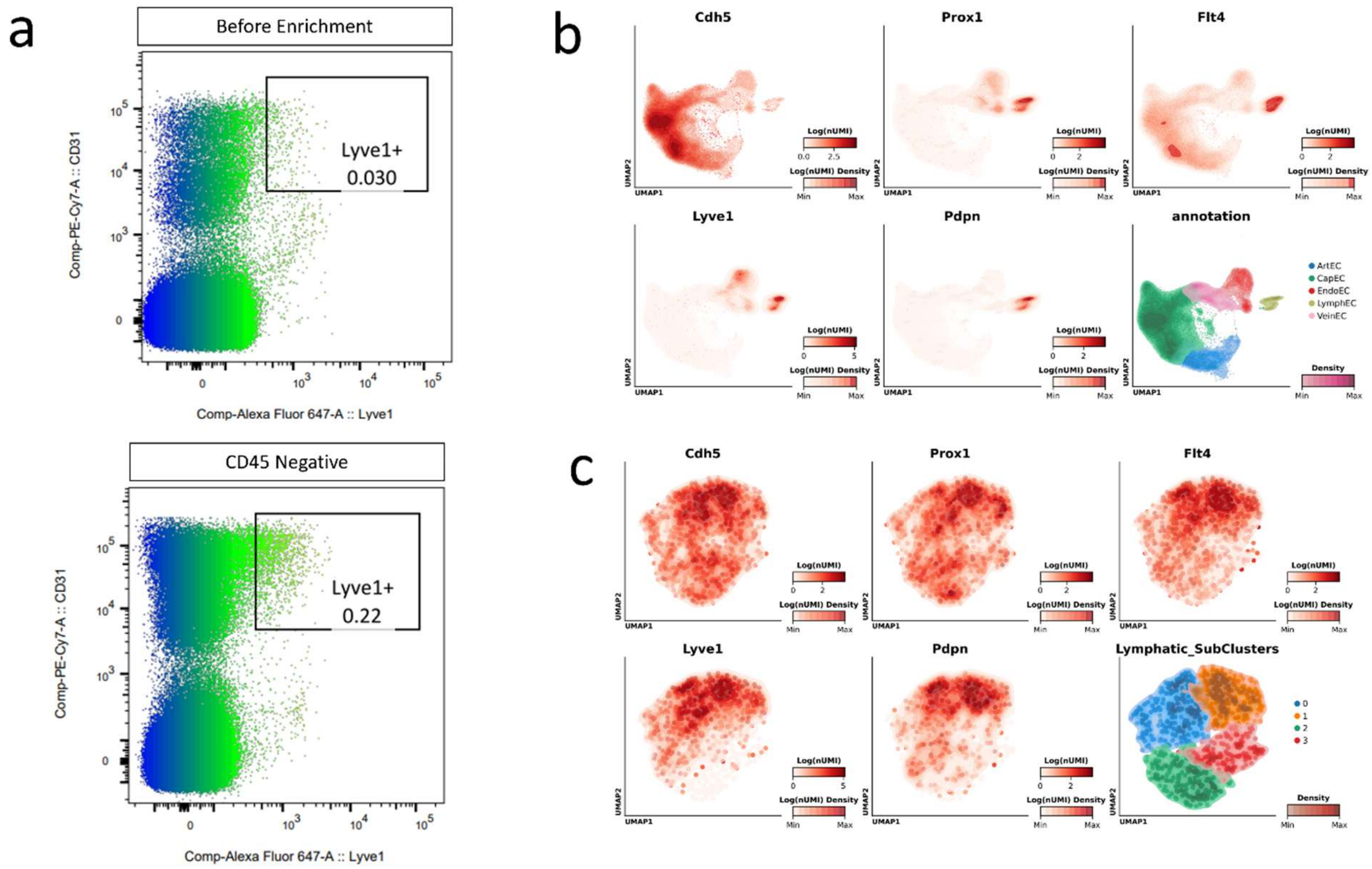


**Figure for the reviewers 2.6: Assessing lymphatic endothelial cells in the murine heart. a.** Flow cytometry of mouse heart homogenates. Shown are CD31, LYVE1 double positive cells before (upper panel) and after negative (bottom panel) selection for CD45-negative cells. **b-c.** Single-cell RNA sequencing of CDH5+ cells from young and old mouse hearts (n=3). Panel b shows the expression of the pan-endothelial marker *Cdh5* and the lymphatic marker gernes *Prox1*, *Flt4*, *Lyve1* and *Pdpn*. Panel c shows the sub-clustering of the lymphatic cluster and the expression of the aforementioned marker genes.

7. The mechanistic significance of nIL33 in aged cardiac lymphatics is suggested but not validated in vivo. In this context, the authors should use a lymphatic endothelial cell–specific IL–33 knockout model to directly demonstrate the role of IL 33 in aged cardiac lymphatics.

**Answer:** The reviewer raises an important aspect. However, the generation of an aged lymphatic-specific *Il33*-deletion line would exceed the time-frame. To study the role of IL33 in vivo, we generated adeno-associated virus, serotype 9 (AAV9) encoding for a short-hairpin RNA (shRNA) targeting the *Il33* mRNA. To enhance the endothelial tropism, we used the Endoglin promotor and coated the AAV9 with an endothelial-targeted peptide (PAMAM-G2$^{CNN}$). The AAV9 were i.v. injected to 18-month-old mice to repress *Il33* mRNA expression in aged lymphatics.

Four weeks after injection, *Il33* expression was reduced on protein level in cardiac lymphatic vessels (**Figure for the reviewers 2.7a,**). Silencing IL33 in aged cardiac lymphatics rescued lymphatic and macrophage densities in the ageing heart (**Figure for the reviewers 2.7b, c**).

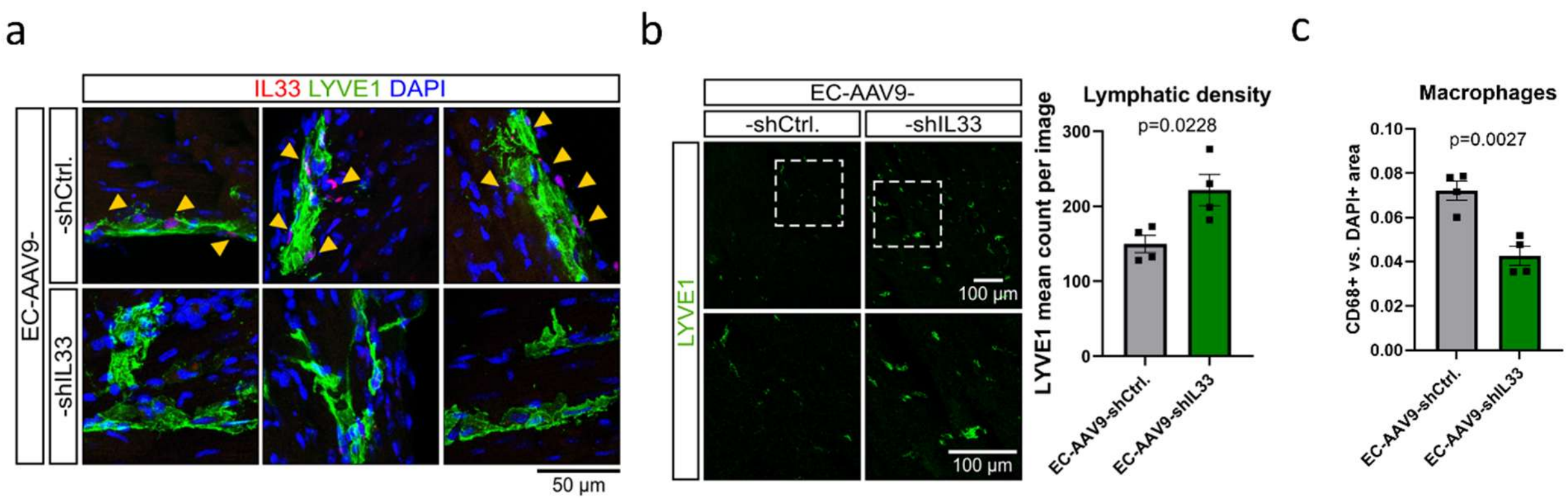


**Figure for the reviewers 2.7:** Repressing IL33 rescues lymphatic density in aged hearts. Modified AAV9 were i.v. injected to 18-month-old male mice to induce the expression of shRNA targeting Il33 (EC-AAV9-shIL33). A non-targeting shRNA served as control (EC-AAV9-shCtrl.). Four weeks after injection, hearts were assessed histologically (n=4). Panel a *shows* representative images of IL33 staining in cardiac lymphatics. DAPI was used as counter stain. Panel *b* shows LYVE1 staining to quantify lymphatic density. *Panel c shows the quantification of macrophages in the heart (n=4).* P-value was calculated using the unpaired, student's t-test.

7a. The authors should include some assessments of recognized cellular aging mechanisms, such as telomere shortening, genomic instability, mitochondrial dysfunction, oxidative damage, autophagy, or epigenetic alterations, to strengthen the rigor of their conclusions.

**Answer:** To address this concern, we performed bulk RNA sequencing on human dermal lymphatic endothelial cells (HDLECs) with and without IL33 overexpression, complemented by targeted functional assays for key aging hallmarks.

The assessment of bulk RNA sequencing data showed no profound changes in senescence core marker gene expressions. Only CDKN1A (encoding for p21) was slightly upregulated in IL33-overexpressing HDLECs (**Figure for the reviewers 2.7.2 a**). In line with this, hallmarks of cellular senescence such as telomer length and senescence-associated β-galactosidase activity remained unchanged, too (**Figure for the reviewers 2.7.2 b, c**). DNA damage as assessed by TUNEL staining was also not induced by IL33 induction (**Figure for the reviewers 2.7.2 d**).

To assess metabolic changes in IL33-overexpressing cells, we quantified mitochondria via TOMM20 staining which were not altered between control and IL33-overexpressing cells (**Figure for the reviewers 2.7.2 e**). We further assessed whether IL33 may affect autophagy. Autophagy-related genes showed no differential regulation (**Figure for the reviewers 2.7.2 f**). Immunofluorescence for p62 and LC3B confirmed unaltered autophagic flux, both in full medium and starvation conditions (**Figure for the reviewers 2.7.2 g**).

Together, these data demonstrate that IL33 does neither induce cellular senescence, nor regulates mitochondrial abundance or autophagy.

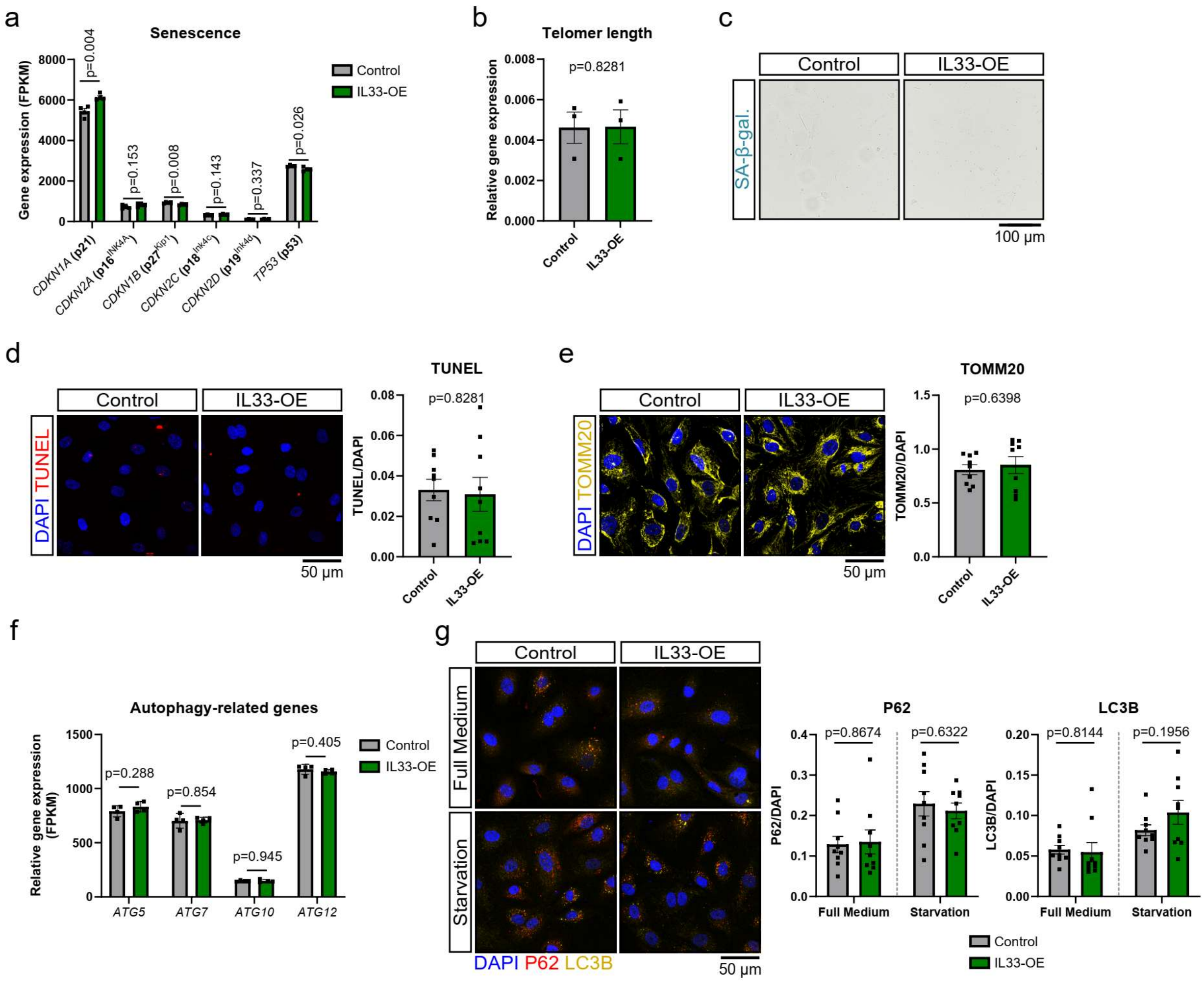


**Figure for the reviewers 2.7.2: Assessing hallmarks of ageing in IL33-OE HDLECs. a.** Bulk RNA sequencing of IL33-overexpressing (OE) vs. control HDLECs (n=4) showing the expression of senescence marker genes. **b.** Quantification of telomer length by quantitative PCR in IL33-overexpressing (OE) vs. control HDLECs (n=3). **c.** Representative images of senescence-associated β-galactosidase staining in control vs. IL33-OE HDLECs (n=3). **d.** TUNEL staining in IL33-overexpressing (OE) vs. control HDLECs (n=9). TUNEL area was quantified and normalized to DAPI area. **e.** TOMM20 staining in IL33-overexpressing (OE) vs. control HDLECs (n=9). TOMM20 area was quantified and normalized to DAPI area. **f.** Bulk RNA sequencing of IL33-overexpressing (OE) vs. control HDLECs (n=4) showing the expression of autophagy-related genes. **g.** P62 and LC3B stainings in IL33-overexpressing (OE) vs. control HDLECs in fully supplemented medium and starvation medium (n=9). P62 and LC3B area was quantified and normalized to DAPI area respectively. Data normally distributed and p-values were calculated using an unpaired, two-sided t-test.

8. Please provide images to support conclusion of Fig. 4n.

**Answer:** We apologize for the omission and included representative images in **Figure 4n of the revised manuscript**.

9. Lines 217-220: The authors should discuss why the lymphatic-secreted factor reelin was not decreased in aged hearts when the main finding of the study is reduced cardiac lymphatic density.

**Answer:** The reviewer mentioned an important aspect. Indeed, Reelin is expressed by lymphatic vessels and a decreased lymphatic density would imply a decline in Reelin. However, in our initial manuscript we

assessed Reelin expression in cardiomyocytes, endothelial cells and fibroblasts of young versus aged mice which revealed no transcriptional change. We confirmed these data by RT-qPCR on whole heart samples. As cardiomyocytes provide the majority of the cardiac RNA content, any lymph-derived changes might be masked.

10. In vivo studies in Fig. 5k-n are underpowered.

**Answer:** We have increased the number of mice as requested by the reviewer.

11. What were the in vivo levels of IL33 and nIL33 in the VegfC overexpression model?

**Answer:** We apologize for the omission. Overexpressing *Vegfc* in aged mice reduces the expression of nuclear IL33 in cardiac lymphatics as shown by **Figure for the reviewers 2.11**. These data are now included in the revised manuscript in **Fig. 5l**. As it is challenging to assesses secreted cytokines per immunofluorescence staining, we cannot make any statements about the cytokine form of IL33.

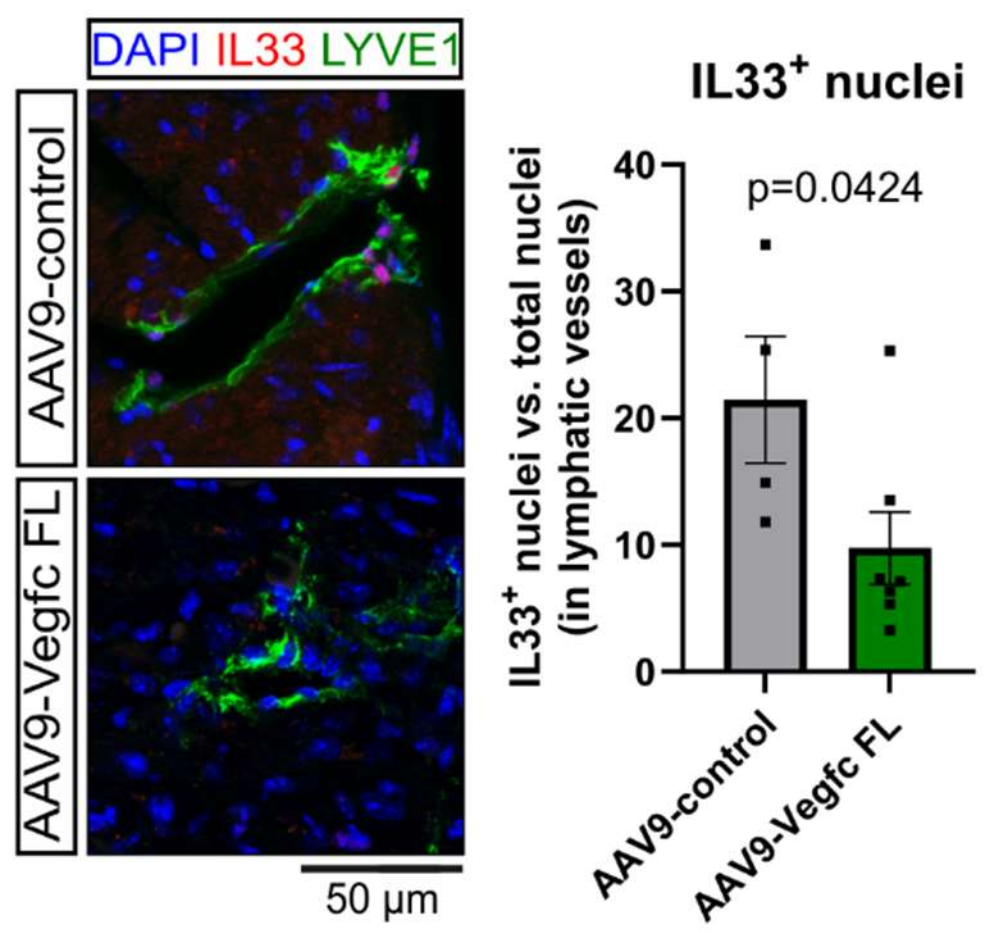


**Figure for the reviewers 2.11: *Vegfc* overexpression reduces IL33 expression in aged cardiac lymphatic.** AAV9-Vegfc FL or AAV9-control were i.p. injected to 18-month-old mice. Eight weeks after injection, hearts were histologically assessed for lymphatic IL33 expression. Mean IL33+ nuclei were quantified (%) per lymphatic vessel. Data are not normally distributed and p-value was calculated using the unpaired, Mann-Whitney test.

12. Line 206 refers to AAV9-Vegfc treatment effects on cardiac diastolic function. Where is the cardiac functional data?

**Answer:** We apologize for the omission. VEGFC overexpression did not affect cardiac function in aged mice. We included these data in **Fig. 5o** of the revised manuscript.

13. A graphical abstract or mechanism figure may be helpful for readers.

**Answer:** We agree with this well-taken point and included a graphical summary.

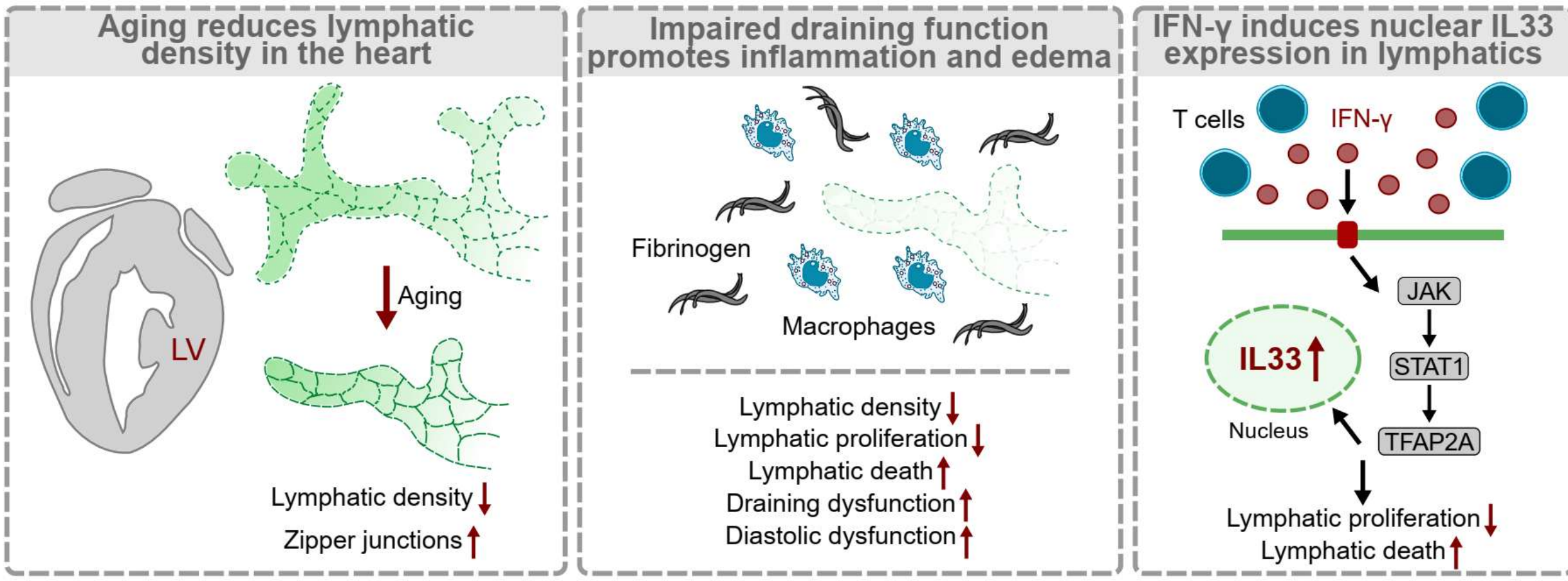


**Graphical abstract.**

14. Line 255-257: "…however, the impact of a chronic reduction of lymphatics and lymphatic cell-derived factors on cardiomyocyte contraction and relaxation properties has not been explored." See PMID: 34789016, PMID: 34403369

**Answer:** We thank the reviewer for providing the literature. We have corrected this statement in the discussion section of the revised manuscript (L289-291) as follows:

[…] however, although chronic depletion of lymphatic vessels and lymphatic-derived factors does not appear to affect systolic function[30,31] […]

**Reviewer #3:** This study by Wagner et al. investigated age-associated changes in cardiac lymphatic vessels. The authors report that cardiac lymphatics are reduced with aging, which is associated with increased inflammation and fibrosis. Using lymphatic-specific Vegfr3 knockout mice or AAV9-mediated overexpression of soluble Flt4 in young mice, they show that reduction of cardiac lymphatics leads to cardiac inflammation and dysfunction, but not fibrosis. They further identify nuclear IL-33 as a potential mechanism underlying age-related loss of cardiac lymphatics and cardiac dysfunction. Specifically, nuclear IL-33 is upregulated in lymphatic endothelial cells in aged hearts. Its expression is increased by IFN-γ but reduced by VEGF-C. The use of in vivo genetic and pharmacological interventions combined with single-cell RNA sequencing and spatial transcriptomics is impressive. Overall, the findings support the concept that enhanced lymphangiogenesis is cardioprotective, particularly in the aged heart. However, several concerns need to be addressed:

1. Experimental design of the Vegfr3 deletion model: The experimental design of the lymphatic-specific Vegfr3 deletion model is not clearly described. Were the mice injected with tamoxifen at day of birth (P0)? The Methods section states that mice were "injected at day 0 and 1... and cardiac function was determined before and 4 and 8 weeks after the first injection," which is confusing. In addition, cardiac functional data from these mice are not shown. Does Vegfr3 deletion also lead to diastolic dysfunction, similar to the AAV9-sFlt4 treatment? In addition, were there any changes in body weight following Vegfr3 deletion or AAV-sFlt4 treatment?

**Answer:** We deeply apologize for this mistake and for being unclear in the method section. We induced *Vegfr3* deletion in lymphatic endothelial cells of adult mice (3 months of age) by injecting tamoxifen on five consecutive days (day 0-4). As echocardiography was not available in the department where the mice were kept, we used MRI instead and assessed heart function before tamoxifen and eight weeks after tamoxifen injection. We corrected these methodological details in the supplementary material and method section. MRI suggests that Vegfr3 deletion leads to diastolic dysfunction which is congruent to the echocardiography data of young mice after AAV9-sFlt4 treatment (**Figure for the reviewers 3.1-1**).

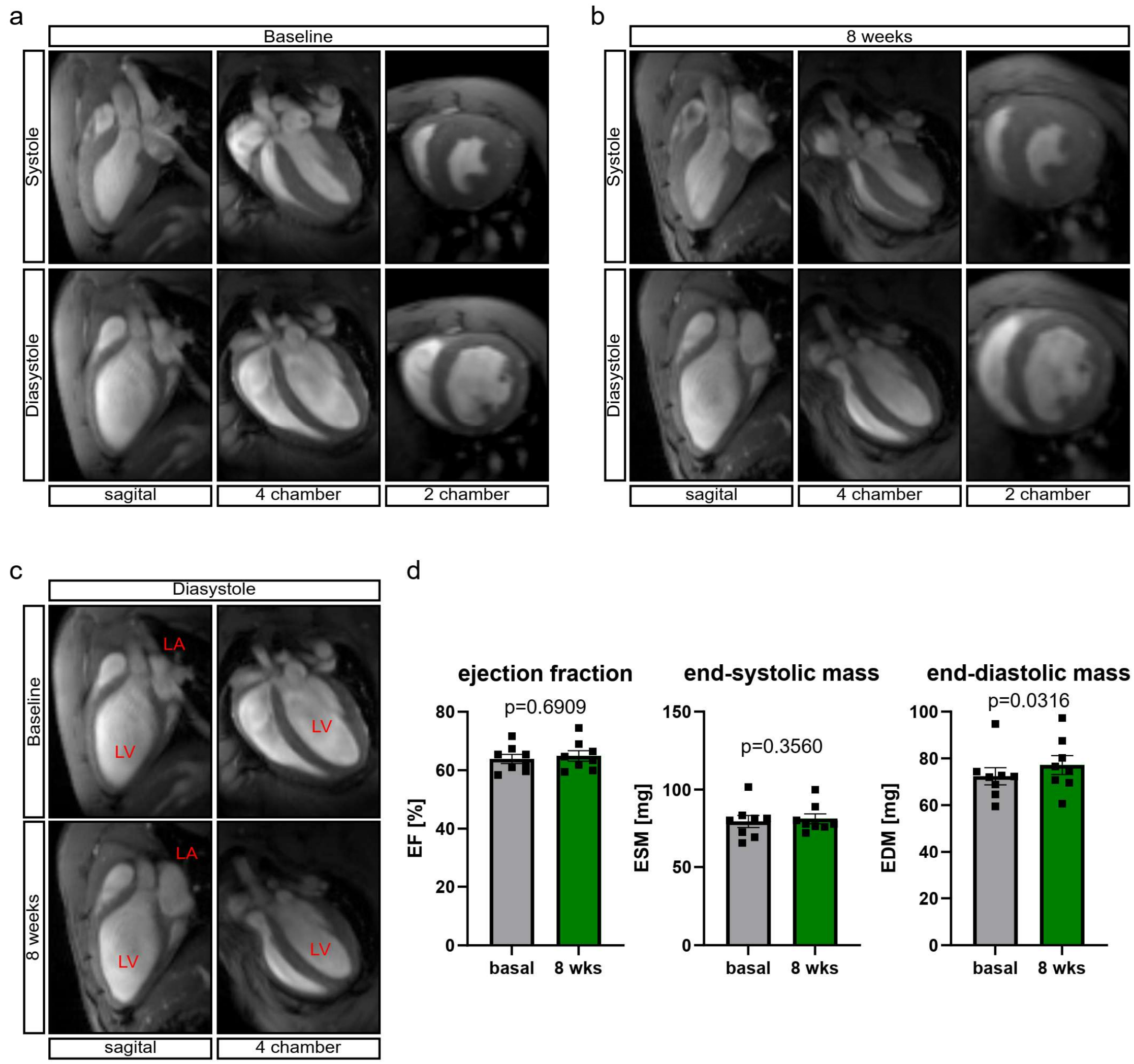


**Figure for the reviewers 3.1-1: Lymphatic-specific *Vegfr3*-deletion impairs diastolic function.** Magnet resonance imaging (MRI) of Vegfr3iΔLEC mice before (**a**) and eight weeks after tamoxifen injection (**b**). Shown are scans at systole and diastole in sagittal, 4-chamber and 2-chamber view. Comparison between baseline and eight weeks post tamoxifen are shown in (**c**) while d shows the quantification of ejection fraction, end-systolic and end-diastolic mass (n=8). Data are Gaussian distributed and p-values were calculated using the paired, student's T-test.

We further determined the body weight of each mouse model and observed no changes in none of the respective models (**Figure for the reviewers 3.1-2**).

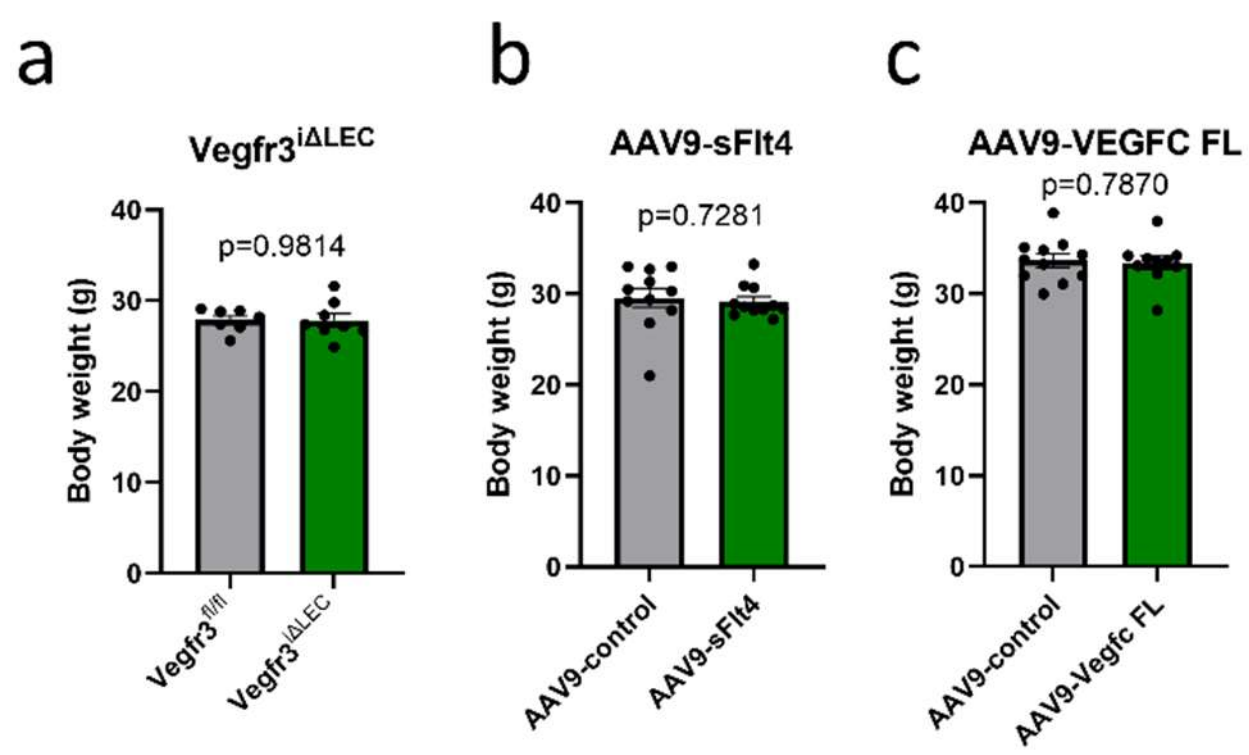


**Figure for the reviewers 3.1-2: Manipulation of VEGFC-VEGFR3 signaling does not affect body weight.** (**a**) Body weight of Vegfr3$^{fl/fl}$ vs. Vegfr3$^{i\Delta LEC}$, eight weeks after tamoxifen injection (n=7 vs. n=8). (**b**) Body weight of 5-month-old mice, eight weeks after AAV9-control vs. AAV9-sFlt4 treatment (n=11). (**c**) Body weight of 20-month-old mice, eight weeks after AAV9-control vs. AAV9-VEGFC FL treatment (n=11 vs. n=10). Data are Gaussian distributed and p-values were calculated using the paired, student's T-test.

2. Identification of nuclear IL-33 as a key mechanism is intriguing. However, the expression of nuclear IL-33 is primarily based on single-cell RNA sequencing of isolated endothelial cells. Although IL-33 is among the top differentially expressed genes in lymphatic endothelial cells, nuclear IL-33 is also known to be expressed in other cardiac cell types, such as fibroblasts. Does nuclear IL-33 expression also change in other cardiac cell populations during aging?

**Answer:** To answer this comment, we leveraged snRNA-seq data from young and aged mouse hearts (Vidal et al., JCI Insight 2019) and plotted the *Il33* expression in **Figure 3.2a for the reviewers**. As the reviewer assumed, *Il33* is expressed by other cardiac cells such as epicardial cells and fibroblast / fibroblast-like cell clusters. However, immunofluorescence labeling demonstrated that the IL33 is predominantly expressed by lymphatic vessels and only barely in non-lymphatic cells (**Figure 3.2b for the reviewers**).

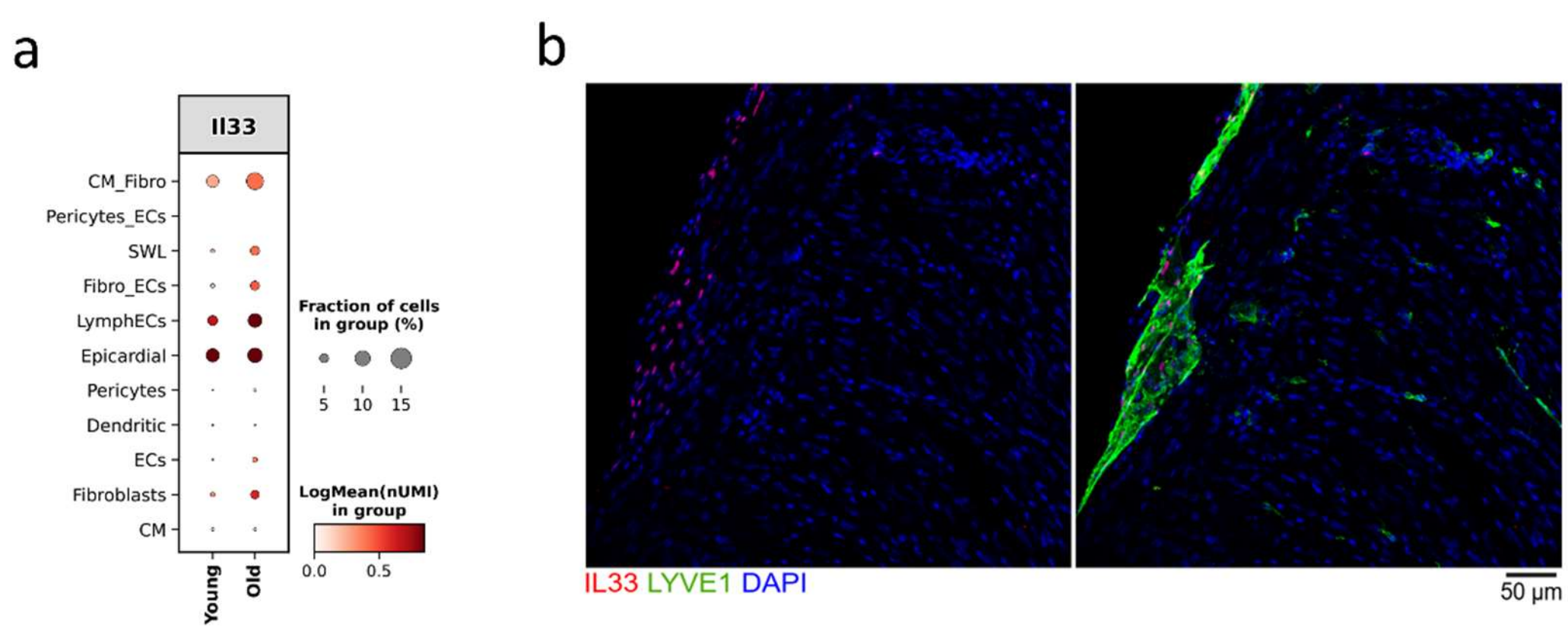


**Figure for the reviewers 3.2:** IL33 expression in the aging heart. **a.** shows the Il33 expression in all cardiac cells using snRNA-seq data from Vidal et al., JCI Insight 2019. **b.** IL33 staining in old mouse hearts. LYVE1 and DAPI staining served as counter stain.

3. Functional studies of nuclear IL-33 are largely performed in vitro. Given that nuclear IL-33 increases beginning at ~16 months of age, does inhibition of nuclear IL-33 in vivo affect cardiac inflammation and

function in aged mice? While the in vitro data clearly show that nuclear IL-33 overexpression inhibits lymphangiogenesis, are there insights into the molecular mechanisms by which nuclear IL-33a exerts this effect?

**Answer:** We thank the reviewer and apologize for the omission. To study the role of IL33 in vivo, we generated adeno-associated virus, serotype 9 (AAV9) encoding for a short-hairpin RNA (shRNA) targeting the *Il33* mRNA. To enhance the endothelial tropism, we used the Endoglin promotor and coated the AAV9 with an endothelial-targeted peptide (PAMAM-G2$^{CNN}$). The AAV9 were i.v. injected to 18-month-old mice to repress *Il33* mRNA expression in aged lymphatics.

Four weeks after injection, *Il33* expression was reduced on protein level in cardiac lymphatic vessels (**Figure for the reviewers 3.3-1a**). Silencing IL33 in aged cardiac lymphatics rescued lymphatic density and reduced macrophage abundance in the ageing heart (**Figure for the reviewers 3.3-1b, c**).

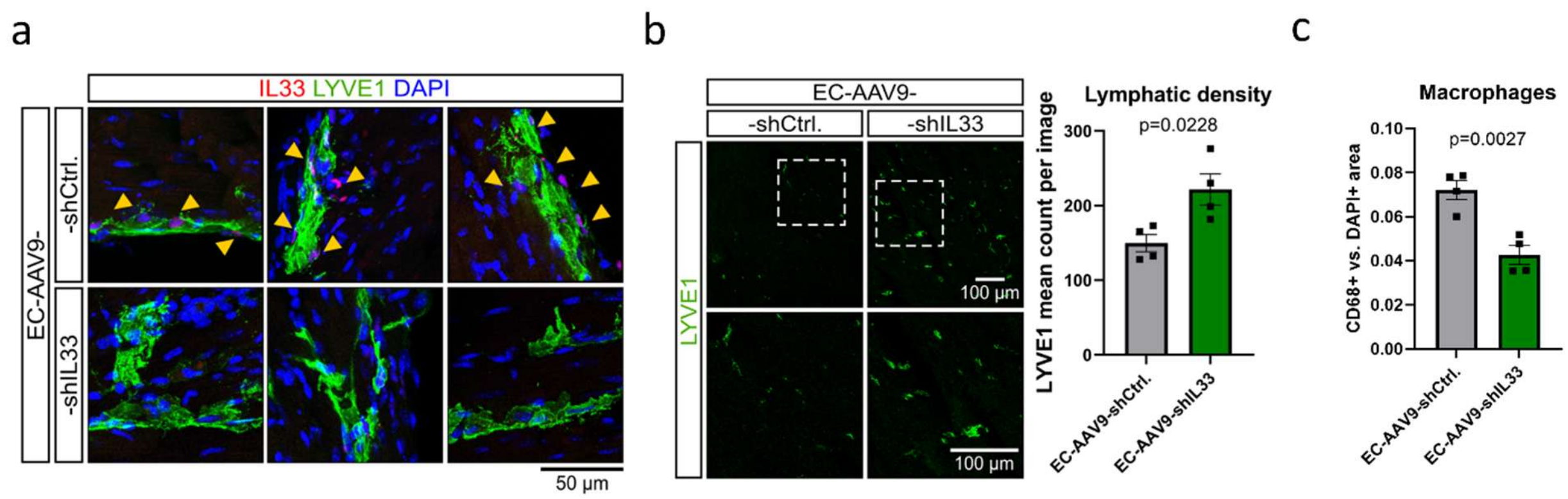


**Figure for the reviewers 3.3-1:** Repressing IL33 rescues lymphatic density in aged hearts. Modified AAV9 were i.v. injected to 18-month-old male mice to induce the expression of shRNA targeting Il33 (EC-AAV9-shIL33). A non-targeting shRNA served as control (EC-AAV9-shCtrl.). Four weeks after injection, hearts were assessed histologically (n=4). Panel a *shows* representative images of IL33 staining in cardiac lymphatics. DAPI was used as counter stain. Panel *b* shows LYVE1 staining to quantify lymphatic density. *Panel c shows the quantification of macrophages in the heart (n=4).* P-value was calculated using the unpaired, student's t-test.

Regarding the comment on how nuclear IL33 may regulate lymphatic angiogenesis, we believe that our data are consistent with the literature showing that nuclear IL-33 is down-regulated during angiogenic activation (PMID: 18787100). In this context, nuclear IL-33 is proposed to function as a chromatin-associated transcriptional regulator that helps maintain a non-proliferative endothelial state, likely suppressing gene programs required for lymphatic proliferation and sprouting. This should be distinguished from extracellular IL-33, which can promote angiogenesis and lymphangiogenesis via ST2-dependent signaling. We therefore interpret our in vitro findings as reflecting the dominant nuclear function of IL33 in HDLECs (PMID: 19661270, PMID: 28878285, PMID: 18787100).

Importantly, our data also show that nuclear IL-33 is associated with increased lymphatic endothelial cell apoptosis and reduced proliferation. This suggests that nuclear IL-33 may impair lymphatic angiogenesis not only through a direct transcriptional mechanism but also indirectly by impairing cellular function: the loss of LECs via apoptosis and their diminished proliferative capacity would secondarily limit the formation and maintenance of lymphatic capillaries. Thus, the reduction in lymphatic capillary density in aged hearts

may reflect this cell-intrinsic loss-of-fitness phenotype driven by nuclear IL-33, while a more direct mechanistic contribution of nuclear IL-33 cannot yet be excluded.

In line with this, our newly generated bulk RNA sequencing data from IL33 overexpressing HDLECs demonstrate reduced expression of genes that are associated with mitosis and cell proliferation (**Figure for the reviewers 3.4-2a, b**). As such, the expression of the cell cycle inhibitor p21 is increased upon IL33 overexpression (**Figure for the reviewers 3.4-2c**).

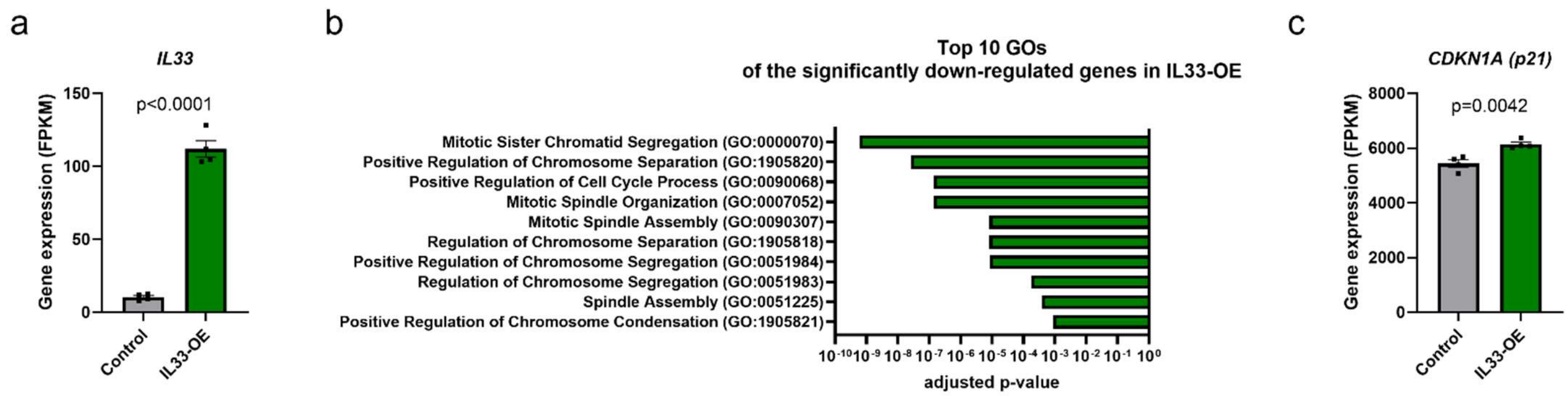


**Figure for the reviewers 3.3-2:** *IL33* overexpression reduces expression of genes that are associated with proliferation. *IL33* was overexpressed via lentiviruses in HDLECs. A control virus served as control (n=4 per group). RNA was extracted and mRNA sequencing was performed **a.** *IL33* expression in control and IL33 overexpressing HDLECs (n=4). **b.** Gene ontology analyses of genes that are significantly down-regulated upon *IL33* overexpression. **a.** *CDKN1A* expression in control and IL33 overexpressing HDLECs (n=4). P-value was calculated using the unpaired, student's t-test.

4. The data indicate that IFN-γ induces, whereas VEGF-C suppresses, nuclear IL-33 expression. Are there mechanistic insights into how these signaling pathways regulate nuclear IL-33 expression?

**Answer:** According to the literature, IFN-γ induces *IL33* expression in epithelial cells via the canonical JAK/STAT1 pathway (PMID: 26986625). To determine whether this pathway also regulates *IL33* expression in lymphatic endothelial cells, we treated human dermal lymphatic endothelial cells (HDLECs) with the JAK inhibitor ruxolitinib (5 µM). JAK inhibition suppressed IFN-γ-induced *IL33* expression (**Figure 3.4a for reviewers**). Likewise, siRNA-mediated *STAT1* silencing reduced IFN-γ-induced *IL33* expression in HDLECs (**Figure 3.4b for reviewers**).

To elucidate how IFN-γ promotes nuclear IL33 expression, we focused on TFAP2A (Transcription Factor AP-2 Alpha). TFAP2A acts downstream of STAT1 (PMID: 16502414) and has been shown to indirectly regulate *IL33* splicing, resulting in the inclusion of exon 3, which contains the nuclear localization signal (PMID: 38456508). Indeed, siRNA-mediated *TFAP2A* silencing decreased *IL33* expression in IFN-γ-treated HDLECs (**Figure 3.4c for reviewers**).

Collectively, these data indicate that IFN-γ activates the JAK/STAT1 pathway to drive *IL33* expression in lymphatic endothelial cells. STAT1 may further induce TFAP2A, thereby promoting the inclusion of exon 3 (nuclear IL33) in the *IL33* mRNA (**Figure 3.4d for reviewers**).

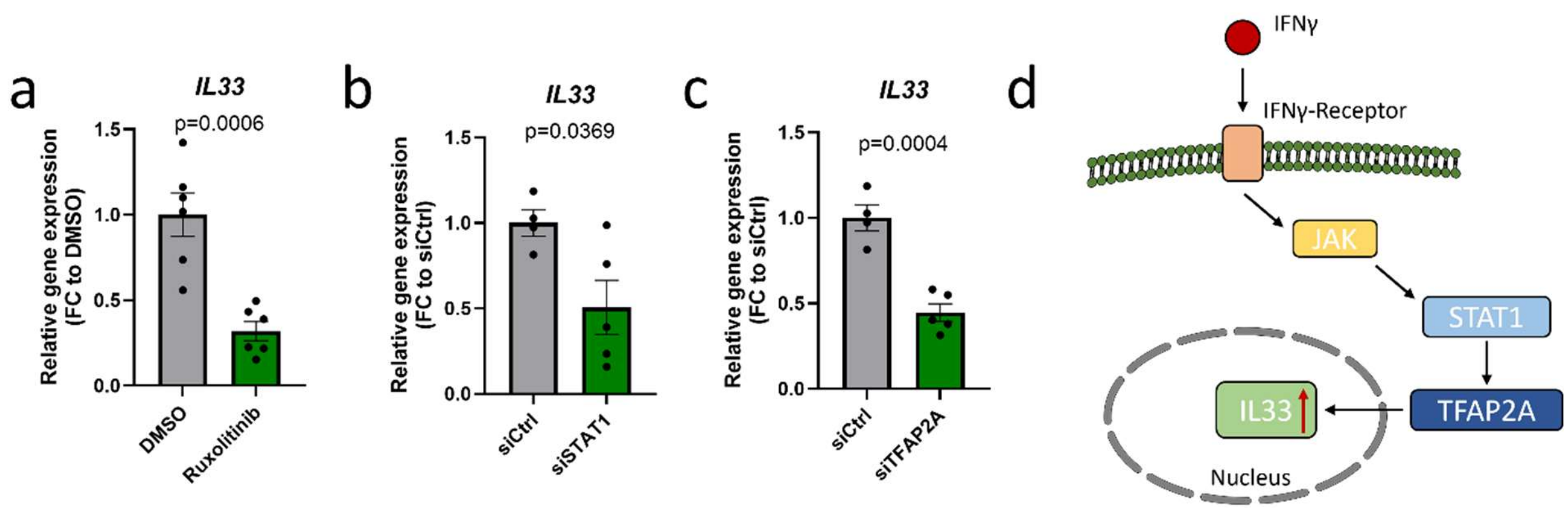


**Figure for the reviewers 3.4:** IFN-γ induces IL33 expression via JAK/STAT1/TFAP2A. **a.** HDLECs were treated with 5 µm ruxolitinib and 100 nM recombinant IFN-γ for 24h. *IL33* expression was determined by RT-qPCR versus RPL0 as housekeeper (n=6). **b.** *STAT1* was silenced by siRNA transfection in HDLECs. Two days after transfection, cells were treated for 24h with recombinant IFN-γ (100 nM). *IL33* expression was determined by RT-qPCR versus RPL0 as housekeeper (n=4 vs. n=5). **c.** *TFAP2A* was silenced by siRNA transfection in HDLECs. Two days after transfection, cells were treated for 24h with recombinant IFN-γ (100 nM). *IL33* expression was determined by RT-qPCR versus RPL0 as housekeeper (n=4 vs. n=5). **d.** Schematic representation of the putative pathway. P-value was calculated using the unpaired, student's t-test.

5. It is somewhat surprising that overexpression of full-length VEGF-C did not alter blood capillary density, given that the mutant form VEGF-C C156S is often used to avoid angiogenic effects on blood vessels. Could the authors comment on this observation?

**Answer:** We thank the reviewer for this important point. Indeed, full-length VEGF-C is known in the literature to be weakly angiogenic, as its proteolytically matured form activates VEGFR-2 on blood endothelium (PMID: 9826710). Unlike the C156S mutant, which blocks this maturation, overexpression of pro-VEGF-C can trigger weak VEGFR-2 activation leading to local vascular changes (dilation, permeability) without inducing sprouting angiogenesis (PMID: 12087065). In our model of AAV9-driven VEGFC overexpression, we observed no significant change in capillary density but rather a tendency towards mild increase of capillary count and perimeter (Supp. Fig. 3), which is consistent with the weak angiogenic potential of full-length VEGF-C (no new sprouts, but potential remodeling effects).

6. The in vitro data show that VEGF-C downregulates nuclear IL-33. In the in vivo VEGF-C overexpression studies, does VEGF-C improve cardiac function in aged mice? Furthermore, is the enhancement of lymphangiogenesis by VEGF-C associated with a reduction in nuclear IL-33 expression in vivo?

**Answer:** We apologize for the omission. Overexpressing *Vegfc* in aged mice reduces the expression of IL33 in cardiac lymphatics as shown by **Figure for the reviewers 3.6**. These data are now included in the revised manuscript in **Fig. 5l**.

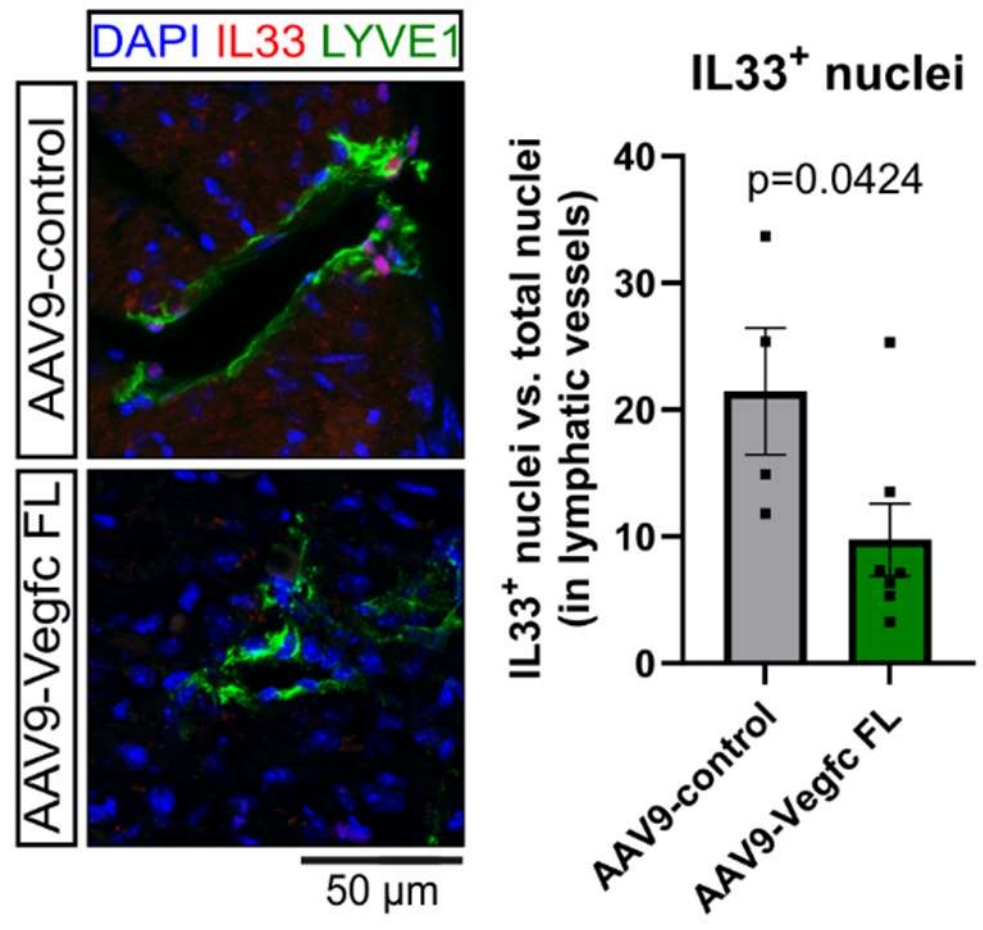


**Figure for the reviewers 3.6: *Vegfc* overexpression reduces IL33 expression in aged cardiac lymphatic.** AAV9-Vegfc FL or AAV9-control were i.p. injected to 18-month-old mice. Eight weeks after injection, hearts were histologically assessed for lymphatic IL33 expression. Mean IL33+ nuclei were quantified (%) per lymphatic vessel. Data are not normally distributed and p-value was calculated using the unpaired, Mann-Whitney test.

We further assessed the heart function of aged mice after AAV9-control vs. AAV9-Vegfc FL treatment. As seen in **Fig. 5o of the revised manuscript**, *Vegfc* overexpression does not improve heart function.

**Second round of revision (Comments from July 23rd 2026)**

**Response to reviewers' comments:**

**Reviewer #1:** The authors have addressed my previous concerns.

**Reviewer #2:** The authors have completed an extensive series of experiments to address most of the suggestions of reviewers. The new data to support a mechanistic pathway for nuclear IL-33 regulation is very valuable, as are expanded references. The in vivo silencing of IL33 to rescue lymphatic density in aged hearts is compelling, convincing and a key data element in support of the overall findings.

**Reviewer #3:** The authors have addressed the comments by providing substantial new data.

Some data were generated solely to address the reviewers' comments and were not incorporated into the revised manuscript. Including some of these key findings in the final version would be informative. For example, the data shown in Figure for Reviewers 1.1 (LYVE1 and CD68 staining) and Figure for Reviewers 1.2 (the strategy for quantifying lymphatic capillaries) would be informative additions

**Answer:** We thank the reviewers for their valuable comments and positive rating. We agree with the suggestions from Reviewer #3 and have included the missing data in the manuscript.

Data from Figure for Reviewer 1.1 has now been included in Extended Data Figure 1c.

The scheme depicting the imaging strategy from Figure for Reviewer 1.2 has now included in Extended Data Figure 1e.

The sequencing data shown in Figure for Reviewer 3.3-2 have now been included in Extended Data Figure 5a-c. The raw sequencing data are available on ArrayExpress (Accession ID: E-MTAB-17485).
