# Supplementary material for: Age-associated loss of lymphatic vessels promotes cardiac inflammation
Source: Nat Cardiovasc Res. 2026 Sep 15;5(10):961–76. doi: 10.1038/s44161-026-00870-y (PMC13658025; doi:10.1038/s44161-026-00870-y)
Supplement: Supplementary file 7 [file 44161_2026_870_MOESM7_ESM.pdf]

c-f

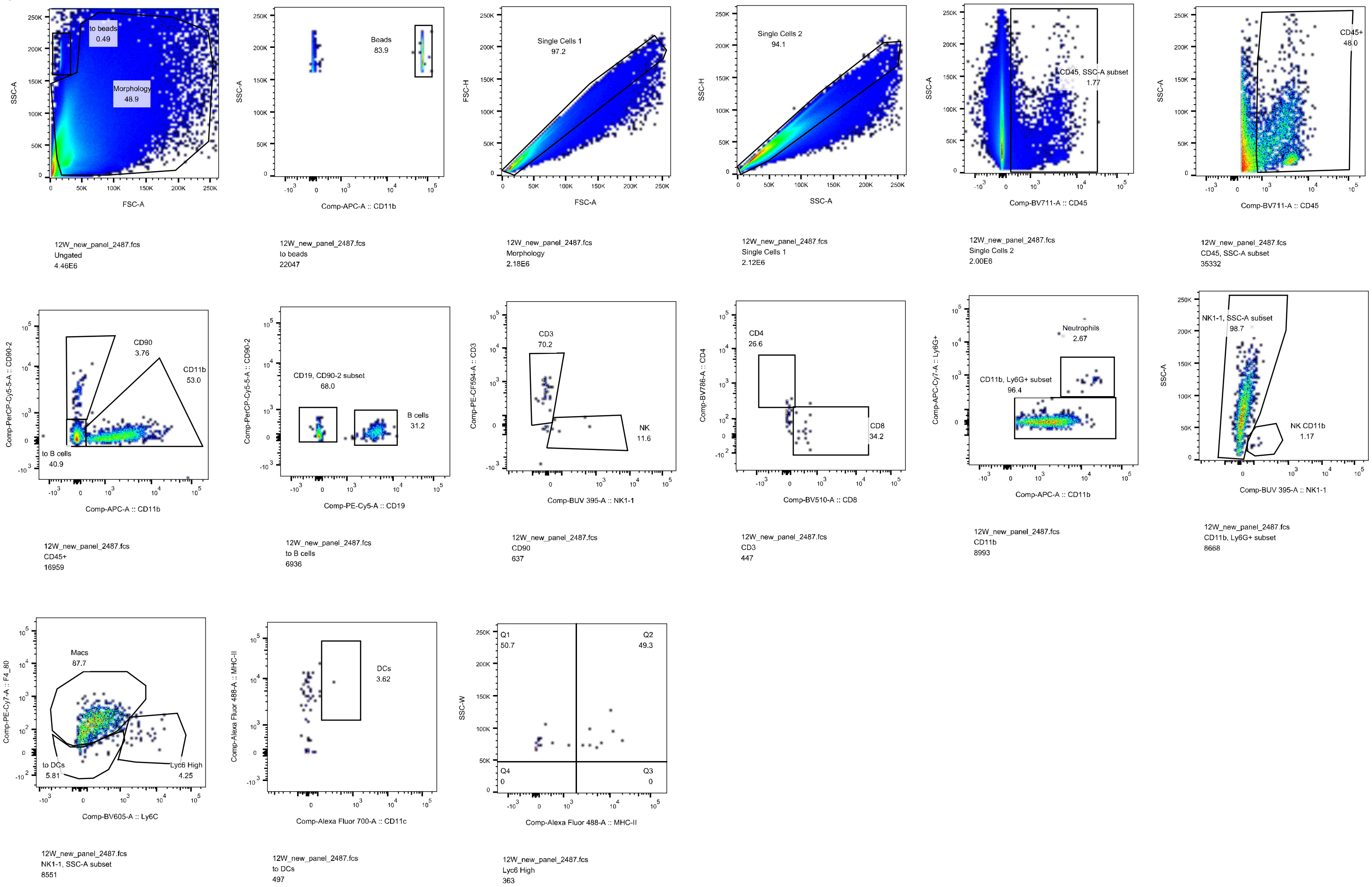

k

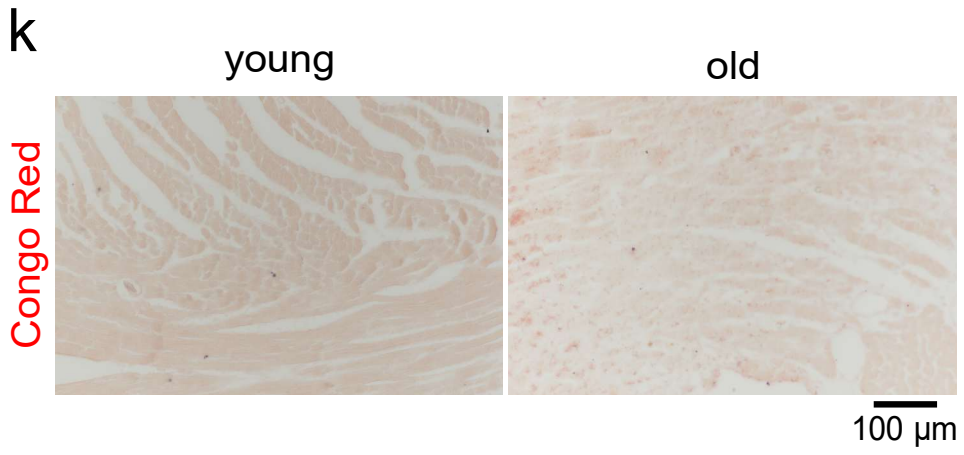

l

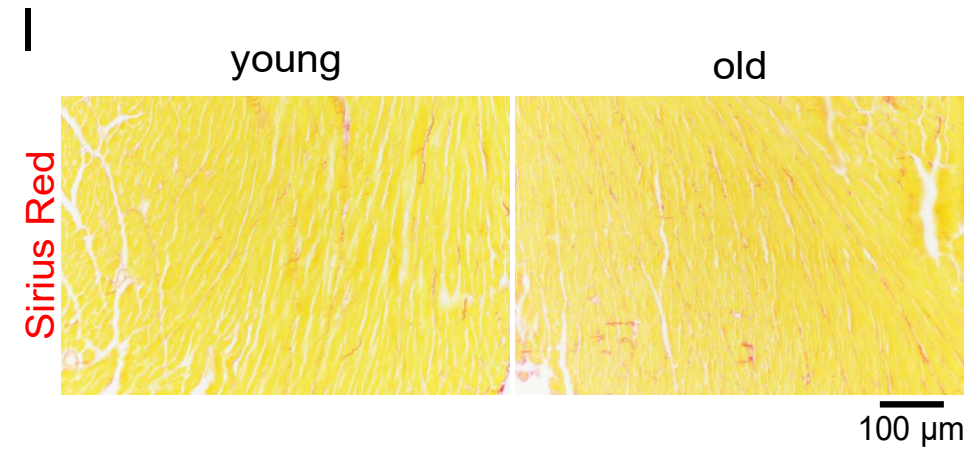

Supplementary Figure 2: Gating strategy to Figure 2c-f and raw uncropped images to Figure 2k, l.
